# Functioning of drug-metabolizing microsomal cytochrome P450s: *In silico* probing of proteins suggests that the distal heme ‘active site’ pocket plays a relatively ‘passive role’ in some enzyme-substrate interactions

**DOI:** 10.1186/s40203-016-0016-7

**Published:** 2016-02-19

**Authors:** Avanthika Venkatachalam, Abhinav Parashar, Kelath Murali Manoj

**Affiliations:** Formerly at PSG Institute of Advanced Studies, Avinashi Road, Peelamedu, Coimbatore, Tamil Nadu 641004 India; Formerly at Hemoproteins Lab, School of Bio Sciences and Technology, VIT University, Vellore, Tamil Nadu India 632014; Satyamjayatu: The Science & Ethics Foundation, Kulappully, Shoranur-2 (PO), Kerala 679122 India

**Keywords:** Cytochrome P450, Heme-enzymes, Mechanism, Substrate binding

## Abstract

**Purpose:**

The currently held mechanistic understanding of microsomal cytochrome P450s (CYPs) seeks that diverse drug molecules bind within the deep-seated distal heme pocket and subsequently react at the heme centre. To explain a bevy of experimental observations and meta-analyses, we indulge a hypothesis that involves a “diffusible radical mediated” mechanism. This new hypothesis posits that many substrates could also bind at alternate loci on/within the enzyme and be reacted without the pertinent moiety accessing a bonding proximity to the purported catalytic Fe-O enzyme intermediate.

**Methods:**

Through blind and heme-distal pocket centered dockings of various substrates and non-substrates (drug molecules of diverse sizes, classes, topographies etc.) of microsomal CYPs, we explored the possibility of access of substrates via the distal channels, its binding energies, docking orientations, distance of reactive moieties (or molecule per se) to/from the heme centre, etc. We investigated specific cases like- (a) large drug molecules as substrates, (b) classical marker drug substrates, (c) class of drugs as substrates (Sartans, Statins etc.), (d) substrate preferences between related and unrelated CYPs, (e) man-made site-directed mutants’ and naturally occurring mutants’ reactivity and metabolic disposition, (f) drug-drug interactions, (g) overall affinities of drug substrate versus oxidized product, (h) meta-analysis of *in silico* versus experimental binding constants and reaction/residence times etc.

**Results:**

It was found that heme-centered dockings of the substrate/modulator drug molecules with the available CYP crystal structures gave poor docking geometries and distances from Fe-heme centre. In conjunction with several other arguments, the findings discount the relevance of erstwhile hypothesis in many CYP systems. Consequently, the newly proposed hypothesis is deemed a viable alternate, as it satisfies Occam’s razor.

**Conclusions:**

The new proposal affords expanded scope for explaining the mechanism, kinetics and overall phenomenology of CYP mediated drug metabolism. It is now understood that the heme-iron and the hydrophobic distal pocket of CYPs serve primarily to stabilize the reactive intermediate (diffusible radical) and the surface or crypts of the apoprotein bind to the xenobiotic substrate (and in some cases, the heme distal pocket could also serve the latter function). Thus, CYPs enhance reaction rates and selectivity/specificity via a hitherto unrecognized modality.

**Electronic supplementary material:**

The online version of this article (doi:10.1186/s40203-016-0016-7) contains supplementary material, which is available to authorized users.

## Background

The cytochrome P450 (CYP) family of enzymes possesses the heme-thiolate functionality and they mediate the phase I metabolism of a vast majority of drugs and xenobiotics in most animals, including man (Testa 1995). Many of these reactions are known to be regiospecific and some of them are even enantioselective (Martinez and Stewart 2000). Since the hydroxylation of several non-activated substrates are not noted with the more commonly found heme-histidylate proteins, CYPs’ catalytic mechanism called for a defined and selective process, which the protein’s "active site" could afford. Therefore, the fundamental step in the catalytic mechanism invoked the formation of high potential intermediate(s) centered at the heme-thiolate moiety, involving an iron - oxygen species (Ortiz de Montellano 2015; Meunier et al. 2004; Denisov et al. 2005; Volz et al. 2002). This proposal was along the lines of a two-electron deficient catalytic species identified as Compound I observed in heme peroxidases (Raven and Dunford 2015). Thereafter, the substrate’s interaction with this enzyme active intermediate is understood to occur by an ‘oxygen-rebound’ mechanism (Groves 1985). As per the prevailing understanding, the formation of this catalytic CYP intermediate solicits a highly fastidious, multi-step, ordered process involving termolecular complexations of CYPs with cytochrome P450 reductase (CPR), cytochrome *b*_5_ (Cyt. *b*_5_), substrate, molecular oxygen etc. (Guengerich and Isin 2008). Such a catalytic cycle requires that the diverse substrates (herein considered as the final oxygen atom acceptor) bind to a given distal heme pocket of a CYP at the very first step (and stay bound till the very end), to induce a redox potential change of the heme-iron for the overall cycle (for protein-protein electron transfer, substrate hydroxylation and superoxide/peroxide/water production) to be feasible (Guengerich and Isin 2008). Therefore, the prevailing CYP catalytic mechanism obligatorily espouses a high-affinity binding and positioning of the diverse substrates at a favorable locus within the distal heme pocket (also known as the “active site”). When the substrate gets converted to the product, the latter is supposed to lose affinity for the enzyme and hence, it detaches and diffuses out of the ‘active site’.

“Lock & Key” (Fischer 1894) and “Induced Fit” (Koshland 1958) models are routinely used to explain enzyme activity. The currently conceived CYP reaction model employs a version of the latter scheme to explain the substrate selectivity and the former scheme is invoked to explain reaction specificity. It is suggested that the F and G loops/helices are considerably flexible across all CYPs (Poulos and Johnson 2005; Narasimhulu 2010). There is also crystallographic evidence to suggest that CYPs have “closed” and “open” conformations (Poulos 2014). This finding is taken to support the suggestion that a CYP can open up for a substrate and then close itself upon the substrate after its presentation, thereby “committing it to catalysis” (Lu 1998). The erstwhile hypothetical paradigm is challenged by the fact that CYPs are typically characterized by broad selectivity/specificity. That is, a given CYP enzyme might catalyze the metabolism of a diverse array of substrates of various topographies and dimensions (containing functionally distinct moieties), at multiple loci of the given drug molecule (Sugimoto and Shiro 2012; Ekins et al. 1999; Lewis and Dickins 2002). The crystal structures of major CYPs are known today, complexed with some of their known substrates (Williams et al. 2003; Wester et al. 2004; Ekroos and Sjögren 2006; Sevrioukova and Poulos 2012). In many of these systems, the substrate is positioned too far from the heme-centre for a direct attack at the reactive moiety (Ekroos and Sjögren 2006). Also, it is difficult to visualize why/how a constrained active site of CYP would not give an enantioselective or regioselective reaction for some large molecules. Further, it is intriguing how some of the most sterically obstructed sites within a given substrate is hydroxylated when there are other favorable loci available within the very substrate molecule (given the understanding that Compound I, the enzymic reactive intermediate, is supposedly a high potential species). Therefore, some spatial considerations apparently challenge the hitherto available ‘high affinity substrate binding at active site’ theory.

A few years back, we had proposed alternative modalities of substrate interactions for the heme-thiolate (extracellular, fungal) enzyme, chloroperoxidase (Manoj 2006; Manoj and Hager 2008). Recently, we have also solved several aspects of heme-enzyme activations, inhibitions and other kinetic observations using appropriate biochemical experimental controls and logical deductions (Gideon et al. 2012; Manoj et al. 2010a; Manoj et al. 2010b; Parashar et al. 2014; Parashar and Manoj 2012). In these works, we went beyond the purely classical substrate-binding based Michaelis-Menten kinetics. We wondered if these ideas would be relevant in CYP-drug metabolism mechanistic chemistry. Molecular docking and theoretical predictions (based on dataset training/modeling/dynamic simulations) are an efficient and accepted way of understanding structure-function correlations (Yuriev et al. 2015; Cross and Cruciani 2010; Scotti et al. 2015; Hlavica 2015; Olsen et al. 2015; Cruciani et al. 2005; Mudra et al. 2011; Kirchmair et al. 2012; Mendieta-Wejebe et al. 2011; Lewis and Ito 2010). Therefore, we undertook an *in silico* exploration study of the available crystal structures of human microsomal CYPs and probed its “static” docking interactions with diverse “flexible” substrate drug molecules.

## Methods

### Dimensions of small molecules

Dimensions of the substrates were calculated using MarvinSketch 6.2.0 (http://www.chemaxon.com). The option of geometrical descriptors was used for this purpose.

### Crystal structures of proteins employed

Table 1 details the names and references for the pdb files employed in the current study.

**Table 1:** Names and references for enzyme crystal structures explored in the current study

S. No.	Enzyme	PDB identity	Reference(s)
1.	CYP1A2	2HI4	(Sansen et al. 2007)
2.	CYP2A6	1Z11	(Yano et al. 2005)
3.	CYP2C9	1R9O, 1OG2, 4NZ2	(Wester et al. 2004; Williams et al. 2003; Brändén et al. 2014)
4.	CYP2C19	4GQS	(Reynald et al. 2012)
5.	CYP2D6	2F9Q	(Rowland et al. 2006)
6.	CYP2E1	3E6I	(Porubsky et al. 2008)
7.	CYP3A4	1TQN, 3UA1, 2J0D, 2V0M, 4K9T, 3TJS	(Yano et al. 2004; Sevrioukova and Poulos 2012; 2013; Ekroos and Sjögren 2006)
8.	CPO	2CPO	(Sundaramoorthy et al. 1995)
9.	P450_cam_	2CPP	(Poulos et al. 1987)
10.	FAB	1GAF	(Patten et al. 1996)
11.	Estrogen receptor	3ERT	(Shiau et al. 1998)
12.	Cellobiohydrolase	1DY4	(Ståhlberg et al. 2001)
13.	Avidin	2AVI	(Livnah et al. 1993)
14.	Glucokinase	3IDH	(Petit et al. 2011)

### Cavity analysis

Crystal structures of CYPs obtained from RCSB PDB were visualized using PyMol 1.3 (DeLano 2002) and CAVER 3.0.1 (as a plugin in PyMol) (Chovancova et al. 2012) was used to calculate and visualize the tunnels. All molecules were analysed with default parameters except for the minimum probe radius (cut off diameter) which is mentioned against the corresponding entry (Table 2). From the output files, length, curvature and bottleneck data of the proteins were obtained. Substrates from substrate bound protein PDB structure were deleted using Chimera 1.7 (Pettersen et al. 2004) to obtain open and free tunnels.

**Table 2:** Cavity analysis of CYPs by PyMol/CAVER

Enzyme (PDB)	Channels by Pymol [with one H_2_O radius]	Channels by CAVER (cut-off diam. in Å)	Length^a^ (Å)	Vol. (Å^3^)	Curvature ^a^	Bottleneck Dia.^a^ (Å)	Classical substrate dimensions (Å/Å^3^)	Amino acids lining the bottleneck^a^
CYP 1A2 (2HI4)	Nil	3 (1.8)	21.6	406	1.19	1.9	Theophylin 4.81, 4.44; 175	F256, N257, Q258, L116, I117, T118, D119, F260, L261
CYP 2A6 (1Z11)	1 + 1 (proximal)	7 (1.8), 1 (2)	18.0	222	1.54	1.99	Coumarin 4.96, 3.68; 127	G102, E103, F118, Q104, D108, R101, A371, A105
CYP 2C9 (1R9O)	2 + 1 (proximal)	15 (1.8), 9 (2), 6 (2.2), 3 (2.6), 1 (3.4)	13.0	1457	1.25	3.4	Flurbiprofen 6.99, 3.81; 223	S209, I205, V479, E300, A477, E206, S478, T304, L208
CYP 2D6 (2F9Q)	1	18 (1.8)	7.8	797	1.16	4.3	Bufuralol 7.36, 4.91; 267	F120, T309, V370, A305, L484, R374, p373, V308, S304, G306, C443
CYP 2E1 (3E6I)	Nil + 1 (Proximal)	10 (1.8), 3 (2), 2(2.2)	21.9	267 (190 + 77)	1.31	2.28	Chlorzoxazone 5.42, 3.71; 144.02	L368, V364, F478, N367, F207, G479, L363
CYP 3A4 (1TQN)	3	14 (1.8), 4(2), 2 (2.4)	12.7	1508	1.21	2.42	Testosterone 6.67, 4.15; 292.55	Q484, L482, E308, R212, S312, L211
CPO (2CPO)	1	2(1.8), 1 (2.6)	8.7	na	1.14	3.16	CBMS 5.15, 3.12; 118.2	F186, F103, E183, A71, O179, V182
P450_cam_ (2CPP)	Nil	5 (1.8)	25.8	na	1.34	1.98	Camphor 4.19, 3.93; 160.86	V247, T181, M184, L180, L200, D182, F98, G243, T185

^a^Details of highest ranked tunnel in terms of priority. Minimum probe diameter−1.8 Å. Dim./vol. - Maximal projection radius (Å), Minimal projection radius (Å); Van der Waals volume (Å^3^) na- not available; Curvature = length/distance, where length is the length of the tunnel (distance from the calculation starting point to the tunnel ending point calculated along the tunnel axis) and distance is the shortest possible distance between the calculation starting point and the tunnel ending point

### Docking

Crystal structures of CYPs were obtained from RCSB PDB and used as rigid large molecule receptors. Structures of all the substrates used were obtained from PubChem and energy minimized using Chimera 1.7. Protein and ligand molecules were primed for docking using AutoDock tools (MGL Tools 1.5.4) and docked by AutoDock 4.2 (Morris et al. 2009) to explore the binding sites of ligands on the protein. Blind docking was carried out with a grid covering the whole protein for 100 runs to identify putative and unorthodox binding sites inside and outside the active site (represented as Blind Docking or GridB). Refined docking was carried out within well-demarcated grid on the heme active site region (the hydrophobic pocket above heme) of a given protein with each ligand for 100 runs (represented as Centred Docking or GridC). PyMol 1.3 and MarvinView 6.2.0 was used to visualize the output. The conformers or clusters with the lowest binding energy for a given ligand were determined. In the two ligand scenarios of drug interactions study, we used CYPs pre-docked with a ligand as a rigid protein (competitive inhibition for the Enzyme, E; since we thought it was unlikely that a bevy of molecules could have the lesser probable ES + I binding) and docked it against a flexible substrate. RMSD values were calculated using Chimera 1.7 and it was noted that ProFit also gave similar results.

### Homology modelling

For genetic predisposition of drugs study, three dimensional models of single amino acid substitution mutation containing CYPs were generated using SWISS – MODEL (Arnold et al. 2006) against the respective wild type protein structures as templates. The amino acid sequence containing the mutated amino acid was given as input and searched for templates. From the results, the respective wild type protein structure with maximum coverage and identity was chosen for modelling. The output was saved as a PDB file and used for docking, as described earlier.

## Results

### Distal heme active site cavities/tunnels of some CYPs and interactions of the enzyme with substrates

Table 2 details the physical dimensions of- active site cavity, classical substrates and the tunnels from distal heme centre leading to the solvent continuum. Investigation with PyMol showed that the highly versatile CYP3A4 has three channels leading to the distal active site cavity (followed by 2 channels for CYP2C9, another versatile liver microsomal CYP), more than any of the other CYPs. With CAVER, all major CYPs (with relatively higher substrate diversities and greater roles in overall contributions towards drug metabolism, as exemplified by 3A4, 2C9, 2D6 and 2E1) gave 10–18 channels (with a water molecule’s diameter as the limiting constraint). Many of these tunnels were relatively long, twisted or too narrow. One would imagine that such channels would not serve significant roles in the movement of a bulky substrate molecule to the distal heme centre. The amino acids lining the bottleneck of the active site channels were seen to vary significantly across the diverse CYPs. This might suggest little commonality in mechanisms relating to opening/closing of the channels or F/G helices or loops.

Table 3 shows the data for the influence of substrate molecule on the tunnels leading to the distal active site in two prominent CYPs. That is- we probed to see what happens to the channels in the “induced fit” substrate-bound CYPs. In CYP 3A4, when the substrate or inhibitor is bound, no channels were available (for a water molecule to enter the distal active site) (Sevrioukova and Poulos 2012). Comparison of the crystal structures of CYPs without substrates/inhibitor with (i) crystal structure of CYP bound with substrate/inhibitor and (ii) substrate deleted from substrate-bound CYPs gave almost the same data as that of the former. Similar amino acids are still seen and the overall conformation is more or less retained. So, the presence of the substrate does not overtly alter the native structure of the protein for the two prominent microsomal CYPs, as seen from the analysis above. This finding does not imply significant changes in tertiary structure via an induced fit mechanism, as the erstwhile hypothesis would necessitate to explain outcomes.

**Table 3:** Effect of substrate on active site channels of CYPs

Enzyme	Free enzyme							Substrate bound enzyme						Substrate bound enzyme with deleted substrate				
	PDB	N	Length^a^(Å)	Curvature^a^	Bottleneck Dia.^a^ (Å)	Amino acids lining the bottleneck^a^	PDB	Substrate	N	Length^a^(Å)	Curvature^a^	Bottleneck Dia.^a^ (Å)	Amino acids lining the bottleneck^a^	N	Length^a^(Å)	Curvature^a^	Bottleneck Dia.^a^ (Å)	Amino acids lining the bottleneck^a^
CYP 3A4	1TQN	13	24.2	1.24	2.24	A305, R212, C442, G306, T309, F304, I301, Heme.	3UA1	Bromo ergocrytine	0	-	-	-	-	14	20.4	1.21	1.86	A305, T309, G306, C442, Heme
							2V0M	Ketoconazole	0	-	-	-	-	18	25.0	1.37	4.08	A305, L482, S119, T309, C442, I301, I369, A370, F304
							3TJS	@	0	-	-	-	-	16	13.2	1.10	3.4	Heme, A305, I301, T309, S119, F304
							4K9T	Desoxy-ritonavir analog	0	-	-	-	-	24	17.1	1.17	3.8	Heme, A305, T309, F304, I369, C442, I301, G306
CYP 2C9	1OG2	17	15.2	1.34	2.76	T301, A297, L362, G298, G296, C435, Heme	4NZ2	$	6	22.1	1.29	1.15	D293, N107, F114, V113	9	14.5	1.22	1.03	Heme, A297, L366, T301, L362, C435, V113

*N -* Number of predicted tunnels; a - details given for channel with highest ranked priority

@ - Desthiazolylmethyloxycarbonyl ritonavir, $ - (2R)-N-{4-[(3-bromophenyl)sulfonyl]-2-chlorophenyl}- 3,3,3-trifluoro-2-hydroxy-2-methylpropanamide

Table 4 shows the data for blind docking and "active-site" grid-centred docking of several large drug molecule substrates with their CYP counterparts. (The details of molecular structure and reaction schema are given in Additional file 1A, Figure A1A 1 &2.) The blind docking and heme-pocket grid-centred docking gave different results. The first two entries, Trabectedin and Vinorelbine are molecules that are metabolized by more than one CYP. In both these cases, binding at the heme distal pocket is not energetically favorable for CYP2E1. Also, the substrate has a much higher volume than the heme distal pocket of this CYP (Table 2). In heme distal site centred docking, CYP2C9 has very little favorable binding energy, poor orientation and heme-Fe to reactive moiety distance for Trabectedin. For both these molecules, the binding energies were more favorable at alternate locations on the protein for the CYPs (2E1 and 2C9), as evident with the data for blind-docking. For the maverick CYP3A4, at least four substrates (Trabectedin, Benzoxamino-rifamycin, Tacrolimus and Cyclosporin) did not show a favorable binding energy with heme distal pocket grid-centred docking, but the same substrates showed spontaneous binding ability at other locations on the protein. For CYP3A4, comparable (or slightly better) binding energy was seen outside the heme distal pocket for Erythromycin, Teniposide and Itraconazole (in comparison to the active-site grid-centred docking). These substrates gave a favorable binding at several loci on the protein surface, as shown with the blind-docking data. In most of CYP3A4 examples, the heme-pocket grid-centred docking gave poor substrate presentation geometries with heme-Fe to reactive moiety distance ranging ~ 5 to 22 Angstroms. Considering the molecular dimensions of these substrates vis a vis the heme-distal pocket dimensions (as shown in Table 2), it is difficult to envision the parameters of spatial/topographical recognition that subsequently lead to the binding or positioning of such diverse large molecules in the constrained heme distal pockets of the CYPs. A simple analysis of the large substrate molecules (as exemplified in 1, 4, 11, 12, 16 etc. of Table 4) shows that the reaction locus is many times on rather occluded positions (towards the middle and not the ends/tips). Such reactive loci may be accessed by the heme Fe-O species with a major opening up of the protein or inversion of the active pocket, accompanied by significant conformational flexibility on the substrate drug molecules. This would be a low probability event when considering the experimental observation (noted in protein-solution state) that a methyl substitution on an adjacent carbon renders a heterocyclic nitrogen lone pair ineffective from Type II coordination at the heme centre (Jones et al. 2011).

**Table 4:** Docking of very large substrates to CYPs

S. No.	Substrate	Enz.	Reaction	Dimension /Vol. (Å/Å^3^)	Blind Docking			Centred Docking				Ref
					Lowest Energy (kcal/mol)	Distance (Å)	Interactions	Lowest Energy (kcal/mol)	Distance (Å)	Interactions	Presentation	
1	Ecteinascidin/Trabectedin	3A4	N-dealkylation	9.35, 6.23; 664	−3.90	21.1	ASP217 CYS239	+29.90	5.8	LEU438	+	(Vermeir et al. 2009; Reid et al. 2002)
		2C9			−3.24	25.7	ASP49 LEU43	−0.63	19.9	LYS206 LEU195	-	
		2E1			−2.23	25.9	GLN401 GLU402 LYS422	+1699.52	3.0	THR303	+	
2	Vinorelbine	3A4	Deacetylation	9.70, 6.72; 761	−2.48	20.8	LEU211 LYS209 VAL240	−3.10	22.1	LEU211 LYS209 VAL240	-	(Kajita et al. 2000; Topletz et al. 2013)
		2E1			−3.06	30.1	LEU50	+369.73	13.2	THR303	-	(Beulz‐Riché et al. 2005)
3	Tacrolimus	3A4	demethylation	9.48, 8.06; 787	−2.33	24.6	PRO107^a^	+38.21	8.8	ARG105 ARG212 SER119	-	(Lampen et al. 1995)
4	Benzoxazino-rifamycin (Rifalazil)	3A4	hydroxylation	9.22, 8.82; 913	−3.16	32.8	ILE473 THR471	+99.79	9.0	GLY481 SER312 LEU438 ARG212	-	(Mae et al. 1996)
	Cyclosporin	3A4	Hydroxylation	12.28, 7.91; 1218	−1.54	22.6	ARG212 ASP214	+4.61	18.9	LYS173 PRO485 GLU486	-	(Kelly et al. 1999; Ohta et al. 2005)
5	Erythromycin	3A4	N- Demethylation	8.91, 6.49; 729	−2.27	20.8	VAL240 ASP217	−1.06	20.7	GLN484 LYS173 ASP174	-	(Wang et al. 2000)
6	Teniposide	3A4	O-Demethylation	10.67, 7.02; 541	−3.75	13.3	SER437 TYR430 TYR432 PHE435 ASN361	−3.44	8.9	ARG212 SER119 ARG105	(+)	(Relling et al. 1994; Julsing et al. 2008)
7	Itraconazole	3A4	Hydroxylation	11.32, 6.73; 529	−5.31	27.6	PRO227	−4.18	21.1	Val240 LYS173 TYR307 SER311	-	(Isoherranen et al. 2004; Templeton et al. 2008)
8	Bosentan	3A4	Hydroxylation	7.60, 7.22; 478	−2.99	26.5	ARG243 CYS239 VAL240 GLU244	−8.14	10.1	ARG212 GLU374 ARG375 ARG105	-	(Dingemanse et al. 2002; Chen et al. 2014)
9	Zafirlukast	3A4	Hydroxylation	8.85, 8.36; 510	−4.50	25.3	ILE120 LYS115	−9.20	14.8	PHE213 ARG212	-	(Kassahun et al. 2005; Katial et al. 1998)
10	Haloperidol	3A4	Reduction	7.77, 6.05; 342	−4.37	11.9	ARG106 ARG212	−6.98	5.9	ALA305 ARG212	(+)	(Kudo and Odomi 1998)
	Alfentanil	3A4	Dealkylation	6.94, 5.26; 401	−3.05	20.7	VAL240 LEU211	−6.05	9.6	SER119 ARG212	-	(Klees et al. 2005; Kharasch et al. 1997)
11	Pranidipine	3A4	De-alkylation	9.27, 5.62; 401	−5.32	24.0	CYS239 ARG243	−9.47	8.3	ARG105 ARG375 GLU374 ARG212 ALA370	-	(Kudo et al. 1999)
12	Bromoergocriptine	3A4	Hydroxylation	9.96, 6.10; 550	−4.77	28.3	VAL240^a^	−8.04	10.9	SER119 GLU374 THR224 PHE215	-	(Wynalda and Wienkers 1997; Sevrioukova and Poulos 2012)
13	Troleandomycin	3A4	N-demethylation	10.70, 8.04; 781	−2.50	20.9	SER222 ARG243	−2.89	18.0	LYS208 VAL489 GLN484	-	(Yamazaki et al. 1996)
14	Ritonavir	3A4	Demethylation	10.44, 7.54; 585	−1.26	26.9	LYS390	−3.58	19.5	SER312 GLN484 LEU483	-	(Kumar et al. 1996)

Dimensions/volume - maximal projection radius (Å), minimal projection radius (Å); van der Waals volume (Å^2^)

Tacrolimus – 2C9 – GridC – 100^th^ (last) ranked is positioned fully inside hydrophobic pocket with energy → +46.14, Distance – 9.2 Å. (proved with repeat)

Itraconazole – 3A4 – GridB – The 3^rd^ ranked (in the 2nd cluster has better presentation) → 4.18 (10.2 Å)

^a^No direct interactions (neighboring amino acids provided), − bad presentation, + good presentation, (+) moderate presentation (not optimal but not facing the opposite end either)

### Classical marker substrates and their interactions with CYPs

**Fig. 1:**
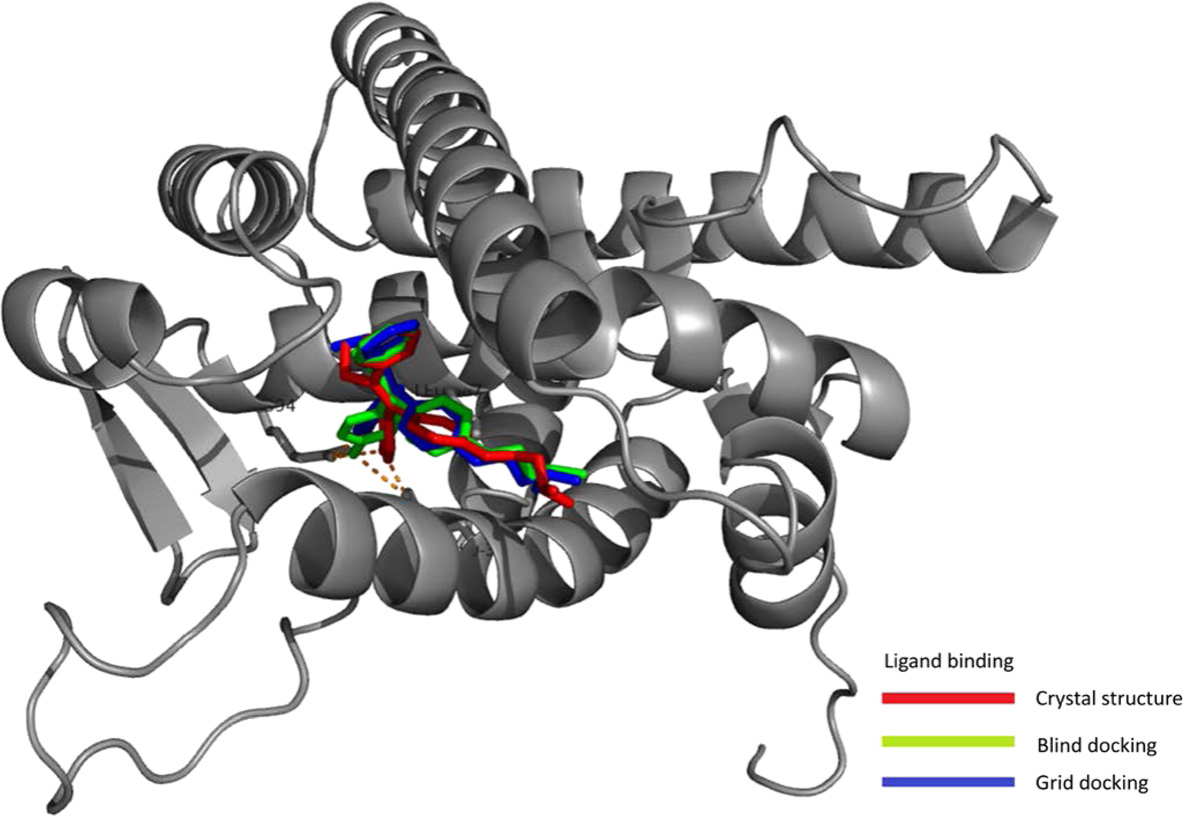
Docking (blind and heme-distal pocket centred) of hydroxytamoxifen to estrogen receptor and comparison with the crystal structure. The RMSD values are given in Table 5

We selected a few well-known high affinity protein-small molecule binding examples as control models of enzyme-substrate binding. The results for these controls are given in Table 5 (and the docked images are available in Additional file 1A, Figure A1B 1–8). Only 3/8 of the blind or centred docks showed RMSD values ≤ 2.5 Angstroms (with respect to the crystal structure). However, a visual examination shows that in the majority of the cases, both blind and centred docking identified the same crypt on the protein as the ligation port (Additional file 1A, Figure A1B). The small molecules bound in similar fashion, albeit the binding energy being a higher value (that is, a higher negative number) in the centred docking. A salient example is presented in estrogen receptor binding to hydroxytamoxifen (Fig. 1). Therefore, the blind docking approach may be considered as a valid methodology for finding out putative binding sites on the protein (Hetényi and van der Spoel 2006) other than the supposed “active site of heme distal pocket”. (This consideration, definitely, does not address the ‘dimensional constraints’ aspect of channels being available for the substrate to access the distal pocket and the dynamics of protein “opening up” or “breathing” in solution state.)

**Table 5:** Controls for blind/centred docking

S. No.	Enzyme-Substrate	Interactions in crystal structure	Docking with the same substrate						PDB ID of protein	Overall RMSD^e^(Å)
			Blind Docking			Centred Docking				
			Lowest Energy (kcal/mol)	Interactions	RMSD (Å)	Lowest Energy (kcal/mol)	Interactions	RMSD (Å)		
1	FAB – Hapten^a^	TYR100 ARG96 HIS35	−3.45^c^	TYR100 ARG50 HIS35 TYR33	5.76	−6.05	TYR100 ARG96 HIS35 ARG50 TYR33	5.06	1GAF	5.91
2	Estrogen receptor - Hydroxytamoxifen	GLU353 ARG394	−7.19	GLU353 ARG394	6.61	−8.08	GLU353 ARG394	1.70	3ERT	1.19
3	Cellobiohydrolase – INP^b^	GLU212 GLN175 GLU217	−3.49	TYR145 TYR171 SER365	1.40	−4.66	TYR145 ARG107	7.82	1DY4	0.99
4	Avidin - Biotin	SER16 SER73 SER75 THR35 THR38 THR40 ASN12 ASN118	−6.30	SER16 SER73 SER75 THR35 THR38 THR40 ASN12 ASN118 ALA39	1.36	−7.25	SER16 SER73 SER75 THR35 THR38 ASN12 ASN118 ALA39	1.14	2AVI	1.26
5	Glucokinase - Glucose	ASN204 ASN231 ASP205 GLU290 GLU256 GLN287 THR168 LYS169	−1.66	GLU27 GLU28 LYS31	31.07	−5.46	ASN204 ASN231 ASP205 GLU290 GLU256 GLN287 THR168 LYS169 SER151	2.82	3IDH	22.11
6	P450_cam_ - Camphor	TYR96	−6.51	TYR96	2.42	−6.93	TYR96	2.50	2CPP	2.42
7	CYP2C9 - Flurbiprophen	ASN204 ARG108	−5.85^d^	ARG108	16.13	−6.59	ASN204 ARG108	5.06	1R9O	11.95
8	CYP3A4 - Erythromycin	SER119	−4.48	SER119	7.02	−6.43	SER119 PHE304 ARG106 GLU374	5.98	2J0D	4.97

^a^5-(Para-nitrophenyl phosphonate)-Pentanoic acid

^b^1-(Isopropylamino)-3-(1-Naphthyloxy)-2-Propanol

^c^Data of 2^nd^ cluster provided. Free energy of first cluster is−3.56 kcal/mol (0.11 kcal/mol higher)

^d^Data of 4^th^ ranked binding in the 1st ranked cluster. Lowest free energy is−5.86 kcal/mol (0.01 kcal/mol higher)

^e^Value obtained by comparing all the three- crystal structure, blind-docked and centred-docks

It is known that many CYPs have marker substrates. (The details of molecular structure and reaction schema are given in Additional file 1A, Figure A1C.) Of the four substrates and CYPs studied for active-site grid-centred docking (Table 6), only two CYP-substrate combinations (2C9-Flurbiprofen & 2D6-Bufuralol) afforded favorable presentation and heme-Fe to substrate reactive moiety distance. This is when many CYPs gave better binding energies or presentation at the active site with non-marker substrates. For example, CYP3A4 gave better values for Flurbiprofen, than its natural metabolizer, CYP2C9. Also, both CYP3A4 and CYP2D6 gave better orientations with Chlorzoxazone (at comparable binding energies) than its natural catalyst enzyme, CYP2E1. These findings indicate that the selection or reaction process need not obligatorily involve binding/catalysis centred in the heme distal pocket alone.

**Table 6:** Marker Substrates and CYPs – Distal heme pocket-centred docking

Substrate →		Flurbiprofen	Bufuralol	Chlorzoxazone	Testosterone
Dim;Vol (Å/Å^3^) →		6.99, 3.81; 223	7.36, 4.91; 267	5.42, 3.71; 144	6.67, 4.15; 293
2C9 (1R9O)	X	4.5	15.2	3.1	9.2
	E	−6.64	−5.73	−4.63	−7.88
	I	ARG204 ARG108	LEU208	ALA297	LEU208
	P	+	-	+	-
2D6 (2F9Q)	X	4.8	4.3	3.2	10.2
	E	−5.81	−6.13	−5.37	−7.33
	I	PHE483 LEU484	GLU216^a^	ALA305	ALA305 LEU213
	P	+	+	+	-
2E1 (3E6I)	X	9.3	6.8	8.2	8.4
	E	−2.13	−3.52	−5.49	+11.67
	I	PHE478	PHE116^a^	ALA299	ALA299 THR303
	P	-	+	(+)	-
3A4 (1TQN)	X	3.3	6.5	5.0	10.0
	E	−8.03	−6.61	−5.64	−6.61
	I	GLU374 ARG375 ARG440	SER119	SER119	ARG212
	P	+	+	+	-

Dimensions/volume - maximal projection radius (Å), minimal projection radius (Å); van der Waals volume (Å^3^); ^a^neighboring amino acids provided, X – distance between heme centre and reaction site (Å). E - lowest binding energy (kcal/mol), I – Interactions. - bad presentation, + good presentation, (+) moderate presentation (Not optimal but not facing the opposite end either)

Therefore, a detailed blind docking study was carried out for eight substrates across six major CYPs. In blind docking (Table 7), most of the classical substrates did not afford a highly fruitful binding at the heme centre. Several drug molecules afforded better binding or presentation at a non-specific CYP heme-distal pocket. Therefore, with the erstwhile understanding, we are at a disadvantage to account for the non-reactivity of these substrates with the “non-specific CYPs”. Further, interactions with key amino acids (which could be an argument for specific molecular triggers involved in an induced fit type of process) were not seen to be critical either, in making or marking a molecule as potential substrate. *In silico* binding of Warfarin to 2C9, testosterone to 3A4, etc. afforded results that disagreed with crystal structures whereas docking of Flurbiprofen to 2C9, Bufuralol to 2D6, etc. agreed with the crystal structures. (These findings are in agreement with our earlier works, Parashar et al. 2014). Of the combinations tested, only CYP2C9 and CYP3A4 (both possessing large distal pockets of ~1500 cubic Angstroms; rendering the heme distal pocket less consequential with respect to spatial constraints) gave similar ranking results for both blind and heme-distal pocket grid-based dockings for their marker substrates.

**Table 7:** Marker Substrates and CYPs – Blind docking

Substrate(CYP preference)→		Theophylin (1A2)	Diclofenac (2C9)	Warfarin (2C9)	Flurbiprofen (2C9)	Mephenytoin (2C19)	Bufuralol (2D6)	Chlorzoxazone (2E1)	Testosterone (3A4)
Dim;Vol (Å/Å^3^) →		4.93, 4.35; 147	6.00, 4.82; 240	6.54, 5.28; 277	6.99, 3.81; 223	5.66, 4.46; 201	7.36, 4.91; 267	5.42, 3.71; 144	6.67, 4.15; 293
1A2 (2HI4)	X	7.0	32.3	24.1	21.1	32.0	32.4	4.7	20.0
	E	−4.16	−4.52	−4.47	−5.39	−3.14	−2.55	−4.55	−4.78
	I	THR124	LYS277 LYS292 LYS293	PRO84 CYS406 LYS404 SER389 ASP110	TYR495 LYS59 ASN60	LEU51 ILE241	LEU242^a^	THR124	TRP466 ARG362
2C9 (1R9O)	X	19.8	18.8	23.7	17.3	11.0	15.2	7.7	20.2
	E	−3.34	−6.15	−5.72	−5.73	−4.31	−4.43	−3.96	−6.48
	I	PHE100	LYS72	THR364	LYS72 PHE100	ALA297^a^	SER209^a^LEU208^a^	THR301^a^LEU366^a^	PHE100 THR364
2C19 (4GQS)	X	6.0	20.7	4.6	4.1	8.8	6.0	5.2	7.7
	E	−3.65	−4.38	−5.44	−4.87	−5.11	−4.82	−4.37	−6.90
	I	GLY296	LYS270	ILE205^a^	THR301^a^	GLY296	LEU202^a^	LEU366^a^GLY296^a^	VAL113^a^
2D6 (2F9Q)	X	7.4	17.3	12.8	20.6	10.5	6.6	7.0	19.6
	E	−4.17	−5.59	−5.17	−5.25	−4.57	−4.28	−5.06	−6.48
	I	ALA305	HIS178 TYR56	SER217^a^	HIS478 GLY479	LEU213^a^	LEU484^a^LEU213^a^	ALA305	HIS478
2E1 (3E6I)	X	6.5	28.3	4.9	17.8	30.6	29.3	27.6	18.2
	E	−3.51	−4.79	+4.52	−4.83	−3.81	−3.86	−4.12	−5.11
	I	PHE298^a^	ARG379 LYS84	LEU210^a^	LYS408	LYS486	ASP470 ARG484	LYS486 LEU463	THR58 ILE361
3A4 (1TQN)	X	5.3	3.5	10.8	10.6	8.3	4.2	4.7	12.8
	E	−4.51	−5.77	−4.79	−5.27	−4.10	−4.45	−3.96	−6.10
	I	ALA370^a^	ARG105	ARG105 ARG212	ARG212	ARG105 ARG212	ARG105	SER119 ARG212	GLY436 PHE435

X – distance between Fe and reaction center (Å), E – lowest energy (kcal/mol), I – Interactions

Dimensions/volume - maximal projection radius (Å), minimal projection radius (Å); van der Waals volume (Å^3^)

Diclofenac – 2C19 the 2nd cluster (9^th^ ranked) is inside active site (−4.35, 4.8 Å)

Testosterone – 2C9 the 3rd cluster (77^th^ ranked) is inside active site (−5.90, 5.9 Å)

Testosterone – 3A4 the 2nd cluster (39^th^ ranked) is inside active site (−5.82, 9.3 Å)

Flurbiprofen – 2C9 the 3rd cluster (56^th^ ranked) is favorably inside active site (−5.33, 4.1 Å)

Theophylin – 1A2 the 2nd cluster (31^st^ ranked) has favorably inside the active site (5.1 Å, −4.04)

^a^no direct interactions (neighboring amino acids provided)

### Docking of select classes of drug molecules (Sartans, Statins & Triptans) with CYP2C9 and CYP3A4

Table 8 shows the docking of different Sartans (The details of molecular structure and reaction schema are given in Additional file 1A, Figure A1D.) to the CYP2C9 pdb files 1R9O and 1GO2 respectively. It is known that Irbesartan and Losartan are efficient substrates, Candesartan and Valsartan are poor substrates and Tasosartan and Olmesartan are not substrates of CYP2C9 (Kamiyama et al. 2007; Berellini et al. 2005; Perrier et al. 1994; Sica et al. 2005; Stearns et al. 1995). Binding of Sartans (that possess a relatively large and well presentable pharmacophore, which contributed to ≥ 1/2 the surface area/volume of the whole drug molecule) with 1R9O were seen to be at different loci in blind and heme-distal site centred dockings. In blind docking, the Sartans always bound with better energy terms outside the heme distal pocket (Additional file 1A, Figure A1E). When considering centred binding at the distal heme pocket, all Sartans (substrates or non-substrates) bind in a similar fashion, with comparable energies and orientation of reaction sites. (The image is shown in Additional file 1A, Figure A1E) In these cases (and also in blind docking), the heme-Fe to reaction centre on the substrate distance is not conducive for direct oxygen transfer. The Sartans and/or its derivative possessing a carboxy moiety on the R-group were either inefficient substrates or efficient inhibitors of CYP2C9 mediated metabolism. Since these molecules bind at identical loci (interacting with the same amino acids) within the heme pocket, the difference in substrate reaction or inhibition potency is difficult to be explained merely by an active site binding hypothesis. More importantly, rather than the active site positioning, substrate reactivity per se and also the interfacial ROS (reactive oxygen species) modulation by such molecules could explain the outcomes (Parashar et al. 2014). With 1GO2, 6/8 of the blind docking and centred docking gave similar clusters. Though binding energies are more favorable in the heme distal pocket centred docking, the distances are still too high to explain for activities. Once again, distal heme-pocket binding mechanism is inadequate to explain the reactivity or specificity of Sartans with this structure also.

**Table 8:** Sartans-CYP2C9

No.	Substrate	Reaction	Dimension /Vol. (Å/Å^3^)	CYP2C9-1R9O top row; CYP2C9-1GO2 bottom row						
				Blind Docking			Centred Docking			
				Lowest Energy (kcal/mol)	Distance (Å)	Interactions	Lowest Energy (kcal/mol)	Distance (Å)	Interactions	Presentation
1	Irbesartan	Hydroxylation	8.11, 6.21; 397	−5.60	24.0	LYS72^a^	−8.62	4.3	ARG108	+
				−6.86	5.2	LEU208 ASN474 GLN214 PHE476	−7.63	3	GLY98 PHE100	+
2	Losartan	Oxidation	7.77, 5.43; 372	−4.59	24.3	PHE69^a^PRO221^a^	−7.98	10.0	GLU104^a^	-
				−5.57	10.8	GLY98, GLY296 PHE100	−7.00	9.9	GLY98 PHE100	-
3	Exp – 3179	Oxidation	8.22, 5.67; 322	−6.92	17.1	LYS72^a^	−8.35	10.0	ARG108^a^	-
				−6.01	10.7	ASN474 GLN214 LEU208 GLY296	−6.88	10.1	PHE476 THR301	-
4	Exp – 3174	Inhibitor	8.14, 5.61; 329	−6.97	26.1	PHE100 PHE69 LYS72	−8.64	10.8	LEU208 ARG108	-
				−5.73	13.8	PHE100	−5.78	7.7	THR301 LEU208 ASN474 GLN214	(+)
5	Candesartan	O- Deethylation	8.24, 5.39; 383	−7.22	16.6	PRO221^a^	−8.40	10.5	LEU208	-
				−5.09	7.0	GLY98 ASN107 GLY296 PHE100	−6.34	8.3	GLY98	(+)
6	Valsartan	Hydroxylation	8.43, 5.94; 410	−5.65	20.7	LYS72	−7.31	8.4	ARG108	(+)
				−4.79	23.7	LYS273	−6.47	7.3	GLY98 PHE100	(+)
7	Tasosartan	(CYP3A4 substrate)	8.59, 5.32; 362	−8.00	nk	PRO221	−9.25	nk	LEU208	nk
				−6.65	nk	PHE100	−8.53	nk	PHE100	nk
8	Olmesartan	Not substrate	8.47, 5.96; 404	−5.26	nk	LYS72 PHE69	−8.15	nk	LEU208	nk
					nk	LEU208 THR301 ASN474	−6.35	nk	LEU208 THR301 ASN474 GLN214	nk

Dimensions/Volume - maximal projection radius (Å), minimal projection radius (Å); van der Waals volume (Å^3^); For 7 & 8, reaction site not known. - bad presentation, + good presentation, (+) moderate presentation (not optimal but not facing the opposite end either); a - no direct interaction, neighborring amino acids provided

The six chosen Statins had two different small pharmacophore groups, as shown in Additional file 1A, Figure A1F. It is known that Fluvastatin and Pravastatin are efficient substrates, Lovastatin is poor substrate whereas Mevastatin, Simvastatin and Atorvastatin are not metabolized by CYP2C9 (Neuvonen et al. 2008; Bellosta et al. 2004; Chapman and McTaggart 2002). The pharmacophore posed ≤ 1/2 area/volume contribution to the whole molecule. The results of docking these molecules to CYP2C9 (1R9O) are shown in Table 9 (and the centred docking image is shown in Additional file 1A, Figure A1G). Two drugs with the common flurophenyl pharmacophore, Fluvastatin (substrate) and Atorvastatin (not a substrate), bind at similar locus in heme-pocket centered docking (though presentation of reaction centre varies), when the most energy-minimized conformations are taken. The second most preferred binding conformer (w.r.t. binding energy) of Atorvastatin presents favorably near the reaction centre, at a comparable energy term (as required by Fluvastatin to achieve the same distance from reaction centre). Therefore, the reactivity of these two statins is not explained by heme-pocket substrate bound reaction model. On the other hand, blind docking gave very different energy values for these two Statins (with much lesser affinity for Atorvastatin in comparison to Fluvastatin) and they were found to bind at different loci on the protein. The four Statins with bicyclohexenyl pharmacophores could be graded into three classes- Pravastatin (good substrate), Lovastatin (weak substrate) and Mevastatin/Simvastatin (not substrates). The hydrophobicity of these Statins increase in the order 2 < 4 < 3 < 5. The binding energies decrease (as expected) and distance of reactive moiety from the iron centre become more favorable for these four statins in the same corresponding order. Of these, three interact with the same amino acid(s) at the active site, although with similar orientation of the pharmacophore and presentation of the reaction centre. Simvastatin (the most hydrophobic of the Statins, not a substrate), afforded the most proximal orientation of the reaction centre (4.8 Å), when compared to the value of 10.6 Å for the substrate Pravastatin (the least hydrophobic of the statins). Mevastatin, though more hydrophobic than Pravastatin, can approach the heme centre at 4.7 Å (for the 10th ranked conformer, with a binding energy of ~ −7.5 kcal/mol), but yet, it is not a substrate. Therefore, once again, “the substrate binding the heme-distal pocket followed by reaction” model falls short at explaining the reactivity of the Statins. In blind docking, the four Statins were found to bind at different loci on the protein (as shown in image Additional file 1A, Figure A1H).

**Table 9:** Statins-CYP2C9

No.	Substrate	Reaction	Dimension /Vol.(Å/Å^3^)	CYP2C9-1R9O						
				Blind Docking			Centred Docking			
				Lowest Energy (kcal/mol)	Distance (Å)	Interactions	Lowest Energy (kcal/mol)	Distance (Å)	Interactions	Presentation
1	Fluvastatin	Hydroxylation, C5-, C6-	6.94, 6.24; 383	−5.05	19.6	PRO221 LYS72	−6.80	7.4	ARG108 ASN204	(+)
2	Pravastatin	Hydroxylation, C3’-	7.65, 6.25; 415	−5.39	20.0	SER53	−7.35	10.6	ARG108 ASN204 GLY296 ALA297	-
3	Lovastatin	Hydroxylation, 6’beta-, C3’-, C5’-	8.19, 5.64; 405	−6.63	19.6	LYS72	−8.18	10.4	ASN204 VAL292	(+)
4	Mevastatin^a^	Hydroxylation, C3”	7.06, 5.42; 385	−6.65	21.0	LYS72	−7.84	10.4	ARG108	-
5	Simvastatin^a^	Hydroxylation, C3’-, C5’-	8.08, 6.34; 422	−6.68	4.9	SER209 ASN474 THR304	−8.80	4.8	SER209 ASN474 THR304	+
6	Atorvastatin^a^	Hydroxylation, C2-, C4-	7.08, 6.62; 517	−2.55	29.1	LYS206 ASN474 PHE482	−5.28	17.9	ARG108	-

Dimension /Volume- maximal projection radius (Å), minimal projection radius (Å); Van der Waals volume (Å^3^)

- bad presentation, + good presentation, + moderate presentation (not optimal but not facing the opposite end either)

The second preferable binding for Atorvastatin is −4.15 kcal/mol, with 3.8 A from iron center, once again binding to Arg 108. For Fluvastatin to achieve the same distance from the reaction center, a binding energy of −5.78 kcal/mol was noted

^a^Metabolized by CYP3A4; underlined numericals have more structurally similar pharmacophores

The docking studies with Sartans and Statins show that although blind dockings of some substrates gave similar results when compared to heme-distal pocket centred dockings, yet other substrates gave quite different docking sites in the blind docking. Also, for a given CYP, several non-substrates were found to have comparable binding energies (and even better relative orientations) to substrates. Further, it is difficult to visualize how a molecule like Atorvastatin could ever get to be oxidized by CYP3A4 at the ortho (and not para) position on the terminal phenyl ring (Additional file 1A Figure A1F), if the reaction were to occur at the spatially constrained heme centre.

Triptans, possessing a central indolyl pharmacophore, were chosen as a probe for CYP3A4 and the results are shown in Table 10. (The details of molecular structures and reaction schema are given in Additional file 1A, Figure A1I.) Only Eletriptan, Almotriptan and Naratriptan are known to be substrates of CYP3A4 (Evans et al. 2003; Salva et al. 2003; Sternieri et al. 2006; Moore et al. 2002; Wild et al. 1999; Vyas et al. 2000). Blind and centred docking gave different binding locations for the Triptans. In the heme distal pocket-centred docking, a substrate is bound in the same manner as a non-substrate. Presentation is not favorable in the active site, with more than 7 Å distance in each. In blind docking, all Triptans bind to a conserved but different locus within the heme-distal pocket. Neither binding energy analysis nor substrate proximity/orientation (within the heme distal pocket) could afford a convincing explanation for Triptan substrate preferences or reactivity for CYP3A4.

**Table 10:** Triptans-CYP3A4

S. No.	Substrate	Reaction	Dimension /Vol. (Å/Å^3^)	CYP3A4 (1TQN)						
				Blind Docking			Centred Docking			
				Lowest Energy (kcal/mol)	Distance (Å)	Interactions	Lowest Energy (kcal/mol)	Distance (Å)	Interactions	Presentation
1	Eletriptan	N-demethylation	6.05, 5.44; 355	−4.99	17.4	CYS239 ARG243	−7.82	7.6	ARG105 SER119 ALA305	-
2	Almotriptan	N-demethylation	6.03, 5.56; 313	−4.00	17.5	CYS239 ARG243 PHE241	−6.66	7.4	ARG372 ARG105	-
3	Naratriptan	N-Demethylation^a^	7.70, 5.52; 312	−4.66	17.3	CYS239 ARG243	−5.52	10.4	GLU374 ARG106	-
4	Sumatriptan	N-Demethylation^a^	6.25, 5.30; 232	--4.39	17.7	CYS239 ARG243 PHE241	−3.77	8.1	ARG106 ARG212	-
5	Zolmitriptan	N-Demethylation (CYP1A2)	5.59, 5.05; 273	−4.28	17.9	CYS239 ARG243	−6.24	10.8	ILE369 GLU374 ARG375 ARG105	-
6	Rizatriptan	N-Demethylation^a^	6.17, 5.56; 259	−3.64	16.5	CYS239	−5.62	7.9	ARG372 SER119	-

Dimensions/Volume - maximal projection radius (Å), minimal projection radius (Å); van der Waals volume (Å^3^). - bad presentation

^a^N-demethylation is assumed as the reaction centre

### Demarcating substrate preferences between related and unrelated CYPs

Table 11 shows the blind and centred docking results for Omeprazole binding with two highly related CYPs- 2C9 and 2C19. Omeprazole is a substrate for CYP2C19, but not of CYP2C9 (Äbelö et al. 2000). In the heme-pocket centred docking, both 2C9 and 2C19 gave similarly bound substrate molecules, with comparable binding energies and orientations. [The docking results (images) are given in Additional file 1A, Figure A1J 1 & 2.] The blind docking gave different Omeprazole binding loci on the proteins (which showed a greater proximity of Omeprazole to the heme centre in CYP2C19), which could tentatively explain the preference of CYP2C19 for the substrate.

**Table 11:** Omeprazole – 2C9 and 2C19

Substrate	Enz.	Blind docking			Centred docking			
		Lowest Energy (kcal/mol)	Distance (Å)	Interactions	Lowest Energy (kcal/mol)	Distance (Å)	Interactions	Presentation
Omeprazole	2C9 (1R9O)	−5.31	19.9	PRO37	−6.99	17.1 (2.5)	LEU208	-
	2C19 (4GQS)	−4.43	12.8	ALA206	−6.51	10.5 (2.6)	ASN107	-

- bad presentation

Table 12 shows the result for the *in silico* binding of various oxyresorufins with two relatively unrelated CYPs- 1A2 & 3A4. For 1A2, alkyloxyresorufin is the preferred substrate, with -C_2_H_5_ being the optimal side-chain and much lower activities being observed with larger or smaller substitutions. On the other hand, 3A4 shows preference for an aryl substitution and little activity with smaller/linear chain substituent (Kenworthy et al. 1999). The heme-pocket centred docking results indicate that enhancement of substitution bulk affords better binding energy terms without any significant alteration of the substrate binding locus and presenting modalities, for all substrates, in both 1A2 & 3A4. The centred docking shows that all four substrates are poorly presented to 1A2. In comparison, 3A4 is seen to bind all four substrates at the active site in relatively favorable modes [Images of these docking results are given in Additional file 1A, Figure A1K 1–4]. Therefore, the heme-distal pocket binding-based reactivity cannot adequately explain the preference of ethoxyresorufin by 1A2 and benzyloxyresorufin by 3A4. In contrast, changing the substituent alters the binding locus and modalities in both 1A2 and 3A4 for blind dockings, thereby offering scope for explanation of kinetic preference of substrates.

**Table 12:** Oxyresorufins- 1A2 and 3A4

Enz. (pdb)	Substrate	Blind docking			Centred docking			
		Lowest Energy (kcal/mol)	Distance (Å)	Interactions	Lowest Energy (kcal/mol)	Distance (Å)	Interactions	Presentation
1A2 (2HI4)	Methoxy-resorufin	−6.02	4.5	THR124	−6.86	11.3	THR124	-
	Ethoxy-resorufin	−5.96	11.3	THR124	−7.15	11.3	THR124	-
	Pentyloxy-resorufin	−5.91	11.6	THR124	−8.05	11.5	THR124	-
	Benzyloxy-resorufin	−4.28	34.0	PHE239^b^	−8.20	11.5	THR124	-
3A4 (1TQN)	Methoxy-resorufin	−6.86	18.0	PHE435 GLY436	−5.67	4.2	ARG105 GLU374 ARG375	+
	Ethoxy-resorufin	−4.22	9.2	ARG212 ARG105 GLU374	−6.11	4.2	SER312 ILE369 LEU483	+
	Pentyloxy-resorufin	−4.57	9.6	ARG105 GLU374 ARG212	−6.60	4.5	ALA370 GLU374 ARG375 ARG105	+
	Benzyloxy-resorufin	−5.45	6.7^a^	ARG212	−7.44	4.0	SER312 LEU483	+

- bad presentation, + good presentation

^a^- 3.8 Å for phenyl ring; ^b^ - no H-bonds or pi-stacking interactions (neighboring amino acids provided)

### Revisiting the classical mutation experiments of RLP Lindberg & M Negishi with the crystal structures of CYPs- 2A6 & 3A4

Coumarin is the natural substrate for CYP2A6 and testosterone is for CYP3A4 (Yun et al. 1991; Wang et al. 1997). It was seen in a pioneering study in 1989 that mutating a single residue (P209L) in CYP2A6 changed it to a testosterone hydroxylating enzyme, akin to CYP3A4 (Lindberg and Negishi 1989). Table 13 reports the docking data for probing these salient observations.

**Table 13:** Coumarin/Testosterone : CYP2A6 (1Z11) & CYP3A4 (1TQN)

Substrate	Enz.	Blind docking			Centred docking			
		Lowest Energy (kcal/mol)	Distance (Å)	Interactions	Lowest Energy (kcal/mol)	Distance (Å)	Interactions	Presentation
Coumarin	2A6	−5.83	5.4	ASN297	−6.33	5.1 (2.8)	ASN297	+
	m2A6	−5.83	5.6	ASN297	−6.16	6.2 (4.3)	ASN297 LEU296	-
	3A4	−5.42	5.0	ARG212 SER119	−5.70	4.7 (3.5)	ARG212 SER119	+
	m3A4	−5.11	4.8	ARG212 SER119	−5.65	4.7 (3.5)	SER119	+
Testosterone	2A6	−4.83	35.6	LYS32	−5.58	8.0 (3.0)	PHE107	-
	m2A6	−4.68	32.1	PRO231 GLN234	−7.83	8.4 (2.9)	MET205	+
	3A4	−6.11	12.8	GLY436 PHE435	−6.61	10.0 (4.5)	ARG212	-
	m3A4	−5.95	12.8	GLY436 PHE435	−6.56	11.7 (7.8)	ARG105 GLU374 ARG375	-

- bad presentation, + good presentation

m2A6 (P209L) – Test – GridB - 82^nd^ and 96^th^ ranks bind near the mutated residue

2A6 – Test – GridB – 87^th^ to 89^th^ ranks bind near the amino acid under study

m2A6 (P209L) – Cou – GridB - 7.8 Å away in 1^st^ rank

2A6 – Cou – GridB – 4.8 Å from 1^st^ rank

m3A4 (L210P) – Cou – 90^th^ to 98^th^ ~ 11 Å from the mutated residue

m3A4 – Cou – 90^th^ to 100^th^ ~ 11 Å from the mutated residue

m3A4 (L210P) – Test – 42^nd^ to 95th bind around 7 Å away from the mutated residue.

m3A4– Test – 50^th^ to 99^th^ ranks bind with ~ 9 Å away from the mutated residue

Data given in braces – nearest distance between any atom of ligand and heme center

For coumarin, both docking grids gave similar results in the two wildtype and the two mutant CYPs. That is- coumarin bound in the same locus in these enzymes (within the distal pocket) regardless of the modality employed for docking or mutations made (Table 13) [The docking results (images) are given in Additional file 1A, Figure A1L 1–4]. Therefore, the hitherto considered mechanism fails to explain the loss/gain of activity when there is an extremely similar binding of coumarin inside the heme distal pocket in the wild type and mutated 2A6/3A4. If the presentation and binding of substrate at the heme distal pocket were to be crucial, then testosterone should be a good substrate for CYP2A6. In CYP3A4, the high loss of activity observed with mutation of Leu 209 (the crystal structure Leu 210) to Phe 209 cannot be explained by a heme-distal pocket centred binding. Leu 209 is ~14 Å away from the heme (and ~7 Å away from the distal pocket channel at the closest locus) and is more closer to the surface (part of the amino acid residue can be visualized located in a small crypt on the surface; (Additional file 1A, Figure A1L 5 and 6) than to the heme floor. It does not form the entrance of any of the three major channels that lead to the distal cavity. Further, it is difficult to imagine testosterone presenting itself in the heme distal pocket in a suitable manner (to undergo oxygen rebound at the heme centre), unless there is a large-scale opening up of the distal site. The two mutations (Ala 117 to Val 117 & Leu 365 to Met 365) that affect both coumarin and testosterone activities of CYP3A4 are stuffed into the protein core, located at ~4 Å and ~10 Å respectively (at the most proximal loci with respect to the distal pocket/channel). In blind docking, testosterone binds to CYP3A4 at a crypt adjacent to Leu 209, with a binding energy of − 4.7 kcal/mol. If binding of the substrate to the surface was important to catalysis, we could explain the mutation’s outcome for CYP3A4. Significant loss of activity for coumarin is seen in CYP2A6 only with a simultaneous alteration of all three residues, Val 117, Phe 209 & Met 365. These resides are found on different loci within the protein (different helices/loops). Met 368 (perhaps the same as Met 365 in the earlier literature) is far away from the channel that leads to the heme. Heme pocket - centred docking of testosterone with the homology models (with the mutated residues) gave slightly different results, when compared to the wild type. But these slight differences in binding energy and distance also fail to explain the complete reversal of activity of 2A6 and 3A4.

### Genetic predispositions

In the first study, allelic variation of 2C9 activity on Bosentan was studied. The alleles 2C9*13 (L90P) and 2C9*43 (R124W) had very low Bosentan clearance (<1 % of control) whereas 2C9*55 (L361I) has very high activity (Chen et al. 2014). It should be remembered that Bosentan is a very large substrate and it poses little scope for the direct presentation of its reactive moiety at the iron centre of distal heme pocket. Homology models of the proteins were prepared and it was seen that none of the amino acid substitutions caused a conformational change in the heme distal pocket region or the overall structure of the protein. The variant 2C9*13 (with significantly reduced intrinsic clearance values) had a more favorable binding energy and distance in both blind and grid centered docking (Table 14). In the case of 2C9*43, the slight increase in the distance inside the heme distal pocket does not explain the very low activity seen. In 2C9*55 (the allele with highest Bosentan activity), the least favorable binding (in terms of distance and presentation) inside the active site gave the highest activity. This is yet another indication that a heme-pocket binding based reaction mechanism is inadequate to explain the differences in activity seen in any of the alleles studied.

**Table 14:** Genetic predisposition of drug metabolism

No	Enzyme/ Allele	Mutation	% Activity	Substrate	Docking						
					Blind Docking			Centred Docking			
					Lowest Energy (kcal/mol)	Distance (Å)	Interactions	Lowest Energy (kcal/mol)	Distance (Å)	Interactions	Presentation
1	CYP 2C9	Wild type	100	Bosentan	−2.67	27.9	LEU222	−6.40	3.1	ASN217	+
2	2C9*13	L90P	0.92		−4.75	5.1	PHE100	−6.43	3.0	ASN217	+
3	2C9*43	R124W	0.55		−2.52	29.6	LEU222	−6.37	4.0	PHE100	+
4	2C9*55	L361I	488.89		−2.95	13.4	LEU208	−6.37	11.7	THR301	-
5	CYP 2C9	Wild type	100	S - Warfarin	−5.01	11.1	ASN217	−7.28	10.7	PHE100 ASN217	-
6	2C9*3	R125H	7		−4.99	12.6	PHE100	−7.19	10.8	PHE100 ASN217	-
7	2C9*16	T299A	8		−4.48	12.3	THR301	−7.18	10.8	PHE100 ASN217	-

- bad presentation, + good presentation

In the second study (5, 6 & 7 of Table 14), the allelic variation of CYP2C9 activity on the marker substrate Warfarin was studied. The alleles 2C9*3 (R125H) and 2C9*16 (T299A) have very low Warfarin clearance (DeLozier et al. 2005). The heme-pocket grid centred docking of the wild type and the homology modelled alleles gave almost exact bindings in all rudiments. Once again, the hitherto perceived mechanism does not explain these results.

### Drug-drug interactions

In Table 15, Grid 1 refers to blind docking of the substrate with the protein bound to the top-ranked conformer of modulator from its blind docking. Grid 2 refers to blind docking of the substrate with the protein bound to the nth ranked modulator, which coincides with the substrate’s (lone presentation) binding locus. Overall, 20 instances of drug interactions (as reported in literature, with particular reference to the data furnished by the groups of Houston (Kenworthy et al. 1999), Tracy (Wang et al. 2000) and Birkett (Miners and Birkett 1998) were investigated and the pertinent results are shown in Tables 15 and 16. The structures of the concerned molecules (substrates and modulators) are shown in Additional file 1A, Figure A1M. Nine of these led to inhibitions, nine were instances of activations and two cases were concentration-dependent, leading to either inhibitions or activations. Most importantly, it could be seen that a molecule like quinidine could activate an isozyme like CYP3A4 (10, when Meloxicam is the substrate), inhibit the same isozyme (6, when Nifedipine is the substrate) and activate or inhibit the very isozyme depending upon its concentration (8, when testosterone is the substrate). Further, quinidine could show concentration-dependent effects across CYPs (7 for 2C9 & 8 for 3A4). At the outset, such effects are very difficult to be intuitively or logically accounted for by a purely active site or allosteric binding-based phenomenon. Also, Occam’s razor would suggest that such mechanistic processes would have little significance for physiological evolution, for an active site to develop such intricate patterns of activities.

**Table 15:** Drug-drug Interactions

S. No.	Enzyme + bound ligand	Substrate	Blind docking									Centred Docking					
			Two-ligand scenario						Substrate alone								
			Grid1			Grid2						Two-ligand scenario			Substrate alone		
			E (kcal/mol)	X (Å)	Interactions	E (kcal/mol)	X (Å)	Interactions	E (kcal/mol)	X (Å)	Interactions	E (kcal/mol)	X (Å)	Interactions	E (kcal/mol)	X (Å)	Interactions
1	2C9.Fluvastatin	Diclofenac^a^	−4.94	9.5	ARG108 ASN204	NA			−6.15	18.8	LYS72	−6.79	7.4	ARG108 ASN204	−6.39	17.0	ARG108
2	3A4.Clotrimazole	Erythromycin	−2.15	20.2	VAL240	−2.19	17.3	LYS209 ARG212 ASP214	−2.27	20.8	VAL240 ASP217	+16.85	18.0	GLN484 SER312 SER311 SER315 VAL489	−1.06	20.7	GLN484 LYS173 ASP174
3	3A4.Itraconazole	Testosterone	−6.04	12.8	GLY436 PHE435	−5.50	9.2	ARG105	−6.10	12.8	GLY436 PHE435	−5.65	16.1	GLN484 TYR307	−6.61	10.0	ARG212
4	3A4.Ketoconazole	Testosterone	−6.09	12.7	GLY436 PHE435	−5.71	10.3	ARG105	−6.10	12.8	GLY436 PHE435	−6.33	15.9	GLN484 SER312 TYR307	−6.61	10.0	ARG212
5	2C9.Fluconazole	Warfarin	−5.54	24.2	TRP212	−5.56	28.8	LYS72 ILE223	−5.72	23.7	THR364	−8.32	18.0	GLU104 LEU102	−6.34	12.9	THR301 GLY296
6	3A4.Quinidine	Nifedipine^a^	−3.97	24.3	CYS239 PHE241 ARG243 GLU244	NA			−4.83	9.7	SER119	−4.45	11.7	SER119	−6.55	7.9	ARG212
**7**	2C9.Quinidine	Meloxicam	−6.10	14.3	LEU208	−5.92	30.1	THR364	−5.82	14.4	LEU208	−7.19	4.2	ASN204 GLY296	−6.97	15.3	LEU208
**8**	3A4.Quinidine	Testosterone	−6.09	12.8	GLY436 PHE435	−5.92	24.3	THR364	−6.10	12.8	GLY436 PHE435	−6.14	12.2	GLU374 ARG372	−6.61	10.0	ARG212
9.	2C9.Dapsone	Flurbiprofen^a^	−5.67	5.0	ASN204 ARG108	NA			−5.73	17.3	LYS72 PHE100	−7.19	17.2	ARG108 ASN204	−6.64	4.5	ASN204ARG108
10.	3A4.Quinidine	Meloxicam	−5.32	9.7	ARG212 PHE108 GLU374 ARG372	−4.38	21.0	ARG243 PHE241 VAL240 CYS239	−4.45	10.2	ALA370	−8.53	10.2	PHE213 SER119	−6.95	12.7	SER119 ARG212 ARG105
11.	3A4.Hydroquinidine	Meloxicam^a^	−4.38	20.1	CYS239 PHE241 ARG243 VAL240	NA			−4.45	10.2	ALA370	−9.94	10.1	ARG212	−6.95	12.7	SER119 ARG212 ARG105
12	3A4.Budesonide	Dextromethorphan	−4.42	18.6	ASP217	−5.14	23.4	ASP217 ASP214 ARG243	−4.42	19.0	ASP217	−5.83	10.1	ARG212	−5.19	9.2	ARG212
13.	3A4. Testosterone	Dextromethorphan	−4.49	18.8	ASP217	−4.71	17.8	ASP214	−4.42	19.0	ASP217	−8.03	12.5	ALA370	−5.19	9.2	ARG212
14.	3A4. Diazepam	Dextromethorphan	−4.42	19.0	ASP217	−4.36	14.2	PHE435	−4.42	19.0	ASP217	−6.97	12.2	ARG212	−5.19	9.2	ARG212
15	3A4. Piroxicam	Midazolam	−4.50	14.4	PRO429	NA			−4.62	12.1	ARG372	−9.32	9.2	SER119	−7.09	5.2	ARG212
16.	3A4. Piroxicam	Triazolam	−5.76	11.7	GLY436	NA			−6.30	11.4	GLU374	−8.55	9.1	ARG212	−7.75	11.0	ARG372
17.	3A4. Budesonide	BROD	−4.76	23.4	PHE220 THR224	NA			−5.45	6.7	ARG212	−6.64	7.9	PHE215 GLY481 ALA370	−7.44	4.0	SER312 LEU483
18	3A4.Clotrimazole	BROD	−5.24	12.9	PHE215 THR224	−4.80	19.2	THR224	−5.45	6.7	ARG212	−7.95	8.5	GLN484 LEU483	−7.44	4.0	SER312 LEU483
19.	3A4.Terfenadine	BROD	−4.61	17.4	VAL240	NA			−5.45	6.7	ARG212	−5.92	12.8	PHE213	−7.44	4.0	SER312 LEU483
20.	3A4.Diazepam	BROD	−5.27	12.6	THR224	NA			−5.45	6.7	ARG212	−7.46	11.4	SER119	−7.44	4.0	SER312 LEU483

X – Distance between Fe and reaction center (Å), E – lowest energy (kcal/mol)

Grid 1: Firstly, the top-ranked conformer of modulator from blind docking is taken and then, the substrate is also blind docked

Grid 2: The nth rank of blind docking of modulator, which coincides with the substrate’s preferred (lone presentation) binding locus, is taken as the rigid docking template and then the substrate is blind docked next

2C9.Flurbiprofen - the 3^rd^ cluster (56^th^ ranked) is similarly presented inside active site with energy of −5.33 (4.1 Å) as in 2° docking with Dapsone

Key: Underlined S. No. indicates inhibition, punctuated S.No. indicates activation and bold S. No. indicates concentration dependent effects

^a^Substrates which have the same binding site as their corresponding inhibitor/activator in their individual blind docking

**Table 16:** Binding of modulators with CYPs

S. No.	Ligand	Enzyme	Dimension/Vol. (Å/Å^3^)	Blind Docking			Centred Docking			
				Lowest Energy (kcal/mol)	Distance (Å)	Interactions	Lowest Energy (kcal/mol)	Distance (Å)	Interactions	Presentation
1	Clotrimazole	3A4	5.91, 5.66; 311	−3.83	10.1	GLY436^a^	−5.30	10.2	ARG212^a^	-
		1A2		−3.14	36.3	LEU51^a^	nd	nd	nd	
2	Itraconazole	3A4	11.32, 6.73; 529	−5.31	27.6	PRO227	−4.18	21.1	Val240 LYS173 TYR307 SER311	-
		1A2		−3.50	33.7	ILE241	nd	nd	nd	
3	Ketoconazole	3A4	11.55, 5.40; 453	−3.78	29.1	VAL240^a^	−6.64	10.5	ARG212	-
		1A2		−3.69	37.5	VAL54	nd	nd	nd	
4	Hydroquinidine	3A4	6.72, 5.86; 320	−5.35	4.8	ARG212	−6.70	9.8	ARG212	(+)
5	Quinidine	3A4	6.26, 5.70; 314	−4.19	11.2	ARG105^a^ ARG212^a^	−6.56	9.2	ALA305 ARG212	-
6		2C9		−5.33	18.4	TRP212	−7.01	9.0	LEU208	-
7	Fluvastatin	2C9	6.94, 6.24; 383	−5.05	19.6	PRO221 LYS72	−6.80	7.4	ARG108 ASN204	(+)
8	Fluconazole	2C9	5.35, 5.72; 247	−3.88	4.0	ARG108^a^	−4.65	7.6	GLY296 ARG108	-
9	Dapsone	2C9	6.30, 4.41; 211	−4.36	20.9	TRP212	−5.11	3.2	THR301^a^ ALA297^a^	+
10	Diazepam	3A4	6.10, 5.29; 246	−5.64	9.3	ARG105 GLU374	−6.50	5.0	ARG212	+
11	Terfenadine	3A4	8.61, 5.79; 487	−4.98	17.7	ARG372	−8.33	10.6	ARG212	-
12	Budesonide	3A4	7.77¸ 5.98; 406	−5.10	7.7	ARG212 SER119 ALA370	−6.89	7.7	ARG212 SER119 ARG372 GLU374	(+)
13	Gentamicin	3A4	8.36, 5.99; 476	−1.86	16.8	VAL240 CYS239	−4.77	10.8	ARG212 ARG372	-
14	Roxithromycin	3A4	9.16, 7.03; 829	−0.46	24.8	LYS209 ARG243 ASP217	+2.67	6.9	PHE213 ARG212 GLU374	(+)
15	Nimodipine	3A4	7.48, 5.80; 376	−3.79	25.1	VAL240 CYS239 ARG243	−8.45	10.0	ARG212 ARG375 GLU374	-
16	Nitrendipine	3A4	6.90, 5.65; 318	−4.89	13.7	ARG212 ARG106	−7.51	5.8	ARG212 ARG375 GLU374	+
17	Piroxicam	3A4	8.02¸ 4.83; 269	−4.78	10.0	ARG106	−6.71	12.2	ARG212 ARG372	-

Dimension /Volume - maximal projection radius (Å), minimal projection radius (Å); Van der Waals volume (Å^3^)

- bad presentation, + good presentation, (+) moderate presentation (not optimal but not facing the opposite end either)

^a^No H-bonds or pi-stacking interactions (neighboring amino acids provided)

From Table 15, it can be noted that constrained docking of the modulator at the distal pocket does not prevent the secondary binding of any of the substrates at the active site (except the case 2). We see that inhibitions are not explained with the heme-pocket binding hypothesis because in certain cases (as exemplified by 1 and 2), the distance between heme-Fe and the reaction centre decreases upon the presence of modulator. Further, in examples like 1 & 5, binding energy for the substrate becomes more favorable with the presence of modulator. Regarding activations and a concentration-dependent effect, the active site based reaction mechanism does not provide any consistent explanation either. The most celebrated example of 9 shows clearly that the substrate’s reaction centre goes much farther than the instance lacking the modulator. It is difficult to think that activation could also arise because of multiple molecules’ simultaneous presence in the active site. Further, it is difficult to imagine how the presence of a modulator could affect minute changes in docking modalities, which could in turn enhance rates by ~400 % in 10 & 11 (Kenworthy et al. 1999). In 7 & 8, contrasting results are seen for the same experimental effect of concentration dependent activation/inhibition. That is- in 7, the presence of modulator makes binding energy more negative and Fe-substrate reactive moiety distance decreases; whereas in 8, the presence of the very same modulator makes the binding energy more positive and the Fe-substrate reactive moiety distance increases. In at least four cases (1, 9, 10 & 11), the substrate could still interact with the same key amino acid residues within the actives (in spite of major or minor change in presentation modes).

Blind dockings of various substrates/modulators show diverse binding loci on CYPs’ surface or regions adjacent to proximal/distal cavities. The distance of the reaction centres are diverse and so are the binding energy terms. In certain instances, binding loci are conserved across substrates (Diclofenac and Flurbiprofen bind at Lys 72) and modulators (Dapsone and quinidine bind at Trp 212), as exemplified by 1 & 9 for CYP2C9. This type of paradigm was also seen for many molecules from Statins, Sartans and NSAIDs classes for CYP2C9. (The respective images are shown in Additional file 1A, Figure A1N 1.) In blind docking of individual molecules (Table 15- as seen from 1, 6, 9 & 11- combinations of different CYPs, substrates and modulators), the substrate or modulator molecules bind to the same respective loci within CYP3A4. (Additional file 1A, Figure A1N 2) Even in blind docking with respect to Grid 1, while instances like 6 & 9 (inhibition and activation respectively) could be explained by “active site” considerations, cases like 1 & 11 (inhibition and activation respectively) speak against the erstwhile assumptions. This is further consolidated by the Grid 1 data in cases 2, 3, 4, 5, (for inhibitions) 7 & 8 (for concentration dependent inhibitions/activations) and 10 (for activations), where the substrate binding does not get perturbed significantly by the presence of modulator. Even more, Grid 2 blind dockings, it can be seen that in 2, 3 & 4 (for inhibition), 10 (for activation) and 7 & 8 (for concentration dependent activation/inhibition), the outcome cannot be explained by a heme-pocket binding-based phenomena alone. (The binding energy changes remain similar and the Fe-substrate reaction centre distances are unfavorable.)

Budesonide and testosterone have very similar structure but show varying effects on dextromethorphan metabolism. Budesonide acts as an inhibitor whereas testosterone acts as an activator. From the entries in 12 and 13 of Table 15, it can be seen that the presence of both modulators had very similar effects on dextromethorphan binding across all grids, thus failing to explain the activation and inhibition seen. In another case, the same modulator has varying effects on substrates having similar structures (15 and16). Piroxicam inhibits Midazolam whereas it activates Triazolam metabolism. This is when the docking shows that presence of Piroxicam shifts both of the substrates to the same locus (with similar binding energy). Once again, the erstwhile paradigm does not explain the differences in the activity seen.

In the cases of modulators studied 17 through 20, it is known that Clotrimazole fully inhibits BROD metabolism whereas Budesonide, Terfenadine and Diazepam activate the same by several folds (Kenworthy et al. 1999). In active site docking, the presence of activators pushes the reaction site in the substrate from 4 Å to ~8–13 Å respectively (in some cases, even farther than what the “fully inhibiting Clotrimazole” does), making it non-viable for a more effective direct oxygen rebound. The activation of Dextromethorphan metabolism brought about by Diazepam (14) also follows the paradigm seen above.

Most modulators studied had either a heterocyclic or free amine nitrogen atom, possessing a lone pair. Thereby, they could be potentially capable of affecting the heme centre by a direct Type II interaction. Table 16 shows that all modulators’ (or substrates’) forced binding at the distal heme pocket of the concerned CYPs gave Fe-interactive (reactive) moiety distance ranging from 4 to 20 Å, non-conducive for a direct ligation or oxygen rebound. The distal pocket site docking results shown for the modulators (Table 16) indicate that none of the molecules (except perhaps 9, 10 and 16) present themselves in a favorable way, towards this outcome. While the active sites of the concerned CYPs offer space to accommodate a majority of the modulators, some large molecules (like 2) are docked with a significant part interacting freely with bulk solvent. Further, the orientations of the molecules were many times inappropriate for a preferred modality of substrate activation/interaction. The entries 13 through 16 are not discussed in Table 15 but they are important with respect to Houston group’s data with CYP3A4 (Kenworthy et al. 1999). [Gentamycin did not significantly inhibit or activate any of the 10 substrates of CYP3A4 (or CYP1A2 catalyzed metabolism of EROD). Nimodipine and Nitrendipine (like Nifedipine) inhibited all substrates (lowering CYP1A2 activity for EROD marginally). Roxithromycin inhibited some and did not perturb others (leaving CYP1A2 activity unperturbed).] The binding of these molecules at the distal site of CYP3A4 did not afford any insight upon the experimental effects observed. Another important aspect to note was that azoles like Clotrimazole, Itraconazole and Ketoconazole lowered CYP3A4 activities for all substrates, when all of these ‘azole’ modulators did not possess effective coordination abilities (via the heterocyclic nitrogen) with the heme-iron centre (as seen in entries 1 through 3 of Table 16). Only Clotrimazole inhibited EROD activity of 1A2 and we explored blind docking to see if the ‘azoles’ bound differently with the two CYPs. Blind docking of Clotrimazole to CYP3A4 was at a locus quite adjacent to the CYP’s proximal thiolate and only Itraconazole showed favorable binding in the distal heme pocket (Additional file 1A, Figure A1O). In CYP1A2, the ‘azoles’ docked at a superficial crypt. It is highly unlikely that Clotrimazole or the other large azoles (Ketoconazole & Itraconazole) gains access to the heme distal pocket at low concentrations of the enzyme. While the *in silico* binding of CYP3A4 shows that Ketoconazole binds effectively at the surface of CYP3A4, the crystal structure showed two molecules docked within the heme distal pocket (Ekroos and Sjögren 2006). When compared to the drug metabolism data, the crystal structures do not make much sense because Ketoconazole’s primary metabolizer is CYP3A4 (Fitch et al. 2009). Clotrimazole, a facile one-electron redox active molecule, could even alter the redox status within the microenvironment by directly interacting with superoxide/radicals in free solution.

### Docking of methylstyrenes (for reactions involving oxygen insertion across an activated benzylic double bond) with hemeproteins and the issue of enantioselectivity

It is known that the crystal structure allows the explanation of stereoselective reactions in chloroperoxidase, a fungal soluble P450 (Sundaramoorthy et al. 1998). Dynamic movements of certain residues have also been reported to be responsible for the enantiotopic recognition near the heme centre (Morozov et al. 2011). The conformation of the heme distal pocket is known to critically affect the enantioslectivity involved in CPO. The results of docking of *cis*-betamethylstyrene with CPO/P450_cam_ (Table 17) were compared with *para*-methylstyrene’s aziridination using mutants of P450BM3. In CPO, the presentation of the substrate is close to the heme-iron. Experimentally, this reaction gave high enantiomeric excess (ee) with high product yield (and this was in spite of the relatively harsh reaction system with low pH and high peroxide concentration). In P450_cam_ (with a relatively mild reaction condition at neutral pH and reductant), the access to heme centre is not as closer (but binding energy was marginally better) and as a result, relatively lower ee was observed. (There was no information provided on the yield of the product or side-products formed in the reaction. Since P450_cam_ gives side-products even with camphor and its analogs, *cis*-betamethylstyrene should be no different.) This may be an indication that the oxygen transfer is not strictly mediated at the heme centre in P450_cam_. The recent publication from Arnold group (Farwell et al. 2015) reported the bulky tosyl azide moiety insertion across the styrene’s double bond in an aziridination reaction. This reaction is similar to the oxygen insertion across styrene’s double bond (which gives epoxide). It is seen that the efficient mutant (that affords higher ee and product yield) allows proximity of the double bond to the heme centre and also gave better yields. Therefore, docking with the plastic crystal structure does give some qualitative idea about interactions of substrate at the heme floor in CPO, P450_cam_ and P411BM3 (modified P450BM3).

**Table 17:** Heme-distal pocket centred docking of methyl styrenes with some non-microsomal hemeproteins (CYPs)

No.	Substrate^a^	Enzyme	Lowest Energy	Distance	Interactions	Presentation	EE of prdt.	Yield	Rate	Ref.
1	CBMS	CPO	−4.69	3.5	GLU183^b^ PHE103^b^	+	96 %	92 %	na (~10/ min)	(Allain et al. 1993)
2	CBMS	P450_cam_	−5.60	6.9	TYR96^b^ THR101^b^	-	78 %	na	~1/min	(Ortiz de Montellano et al. 1991)
3	PMS	P411BM3-CIS-T438S^$^	nd	nd	nd		25 %	1.1 %	na	(Farwell et al. 2015)
4	PMS	P411BM3-CIS-T438S (I263F)	−4.42	3.0	ALA264		55 %	40 %	na	(Farwell et al. 2015)

^a^ *CBMS* cis-betamethylstyrene, *PMS* para-methylstyrene, Energy and Distance given in kcal/mol & Angstroms respectively. ^b^No hydrogen bonds or pi-stacking but neighboring amino acids provided. Docking of 4 with tosyl azide gave docked molecules within 2.7 A of the heme iron, with ~ −4.15 kcal/mol energy term and it interacted preferably with ALA330. Docking was not done with 3 owing to unavailability of protein structure

### Can detachment of products from the distal heme pocket (‘active site’) serve as the end of catalytic cycle (as sought by the existent hypothesis)?

Table 18 compares the centred and blind docking data for some CYPs with their substrates and primary products. The binding energy terms of the substrates are comparable to the products, and at times (as is seen in case 1), the products have better binding energy terms than the original substrate. The interaction of substrate and product is very similar for a given enzyme-substrate combination. Some products of a given CYP have more favorable binding energy than another substrate of the same CYP. These simple findings question the assumption that hydroxylations prompt the substrate to leave the active site, owing to a lowering of affinity. Quite simply, there is little chemical logic for the “committed substrate” to leave.

**Table 18:** Comparing the docking of substrates and products

Enzyme (PDB ID)	Ligand	Blind docking			Centred docking			
		Lowest Energy (kcal/mol)	Distance (Å)	Interactions	Lowest Energy (kcal/mol)	Distance (Å)	Interactions	Presentation
3A4 (2J0D)	Erythromycin	−4.48	15.9	PHE304	−6.43	8.4	PHE304 GLU374	(+)
	Nor-Erythromycin	−5.37	6.7	PHE304	−7.91	11.8	PHE304 GLU306	-
2C9 (1R9O)	Flurbiprofen	−5.86	17.8	PHE100 LYS72	−6.59	4.6	ARG108 ASN204	+
	4’Hydroxy flurbiprofen	−5.52	15.8	PHE100 LYS72	−6.17	4.1	ARG108 ASN204	+
2C9 (1R9O)	Diclofenac	−6.15	18.8	LYS72	−6.39	17.0	ARG108	-
	4’Hydroxy diclofenac	−5.37	20.2	LYS72	−5.28	9.1	ARG108	+
2E1 (3E6I)	Chlorzoxazone	−4.12	27.6	LYS486 LEU463	−5.49	8.2	ALA299	(+)
	6’Hydroxy chlorzoxazone	−3.94	28.4	LYS486 LEU463 ASP470 LEU471	−4.75	6.4	ALA299 THR303	-

- bad presentation, + good presentation, (+) moderate presentation (not optimal but not facing the opposite end either)

### Meta-analysis of kinetic and equilibrium constants, residence and reaction times

We analyzed the kinetics data retrieved from literature for two prominent liver microsomal CYPs. As shown in the Table 19, the *K*_M_ (and *K*_i_) for diverse substrate(s) of CYPs 2C9 and 2E1 (determined by different workers, in various reaction systems) ranges between a few micromolar to several hundred or thousand micromolar ranges, which is an “unacceptable” spread in the value of constants.

**Table 19:** Kinetic constants for CYPs, as noted from literature

CYP2C9 Substrate	*K*_M_	*K*_i_	Ref.	CYP2E1 Substrate	*K*_M_	*K*_i_	Ref.
Diclofenac	5.1 ± 0.9 μM (Baculosomes)		(Kumar et al. 2006)	4-Nitrophenol	120–140 μM		(Hanioka et al. 2003)
	2.6 ± 0.3 μM (Supersomes)	1.6 μM			21 μM	42 ± 19 μM	(Tassaneeyakul et al. 1993)
	16 ± 2 μM, 30 ± 3 μM (Reconstituted)	9.3 μM			108 ± 18 μM		(Baranová et al. 2005)
	4.0 μM (HLM)	2.9 μM	(Walsky and Obach 2004; Kumar et al. 2006)		28 μM		(Chen et al. 1996)
	71 μM		(Ngui et al. 2000)		197 μM		(Duescher and Elfarra 1993)
	8.3		(Masimirembwa et al. 1999)		1.84 ± 0.09 mM		(Fairhead et al. 2005)
	9 μM		(Bort et al. 1999)		156 μM		(Van Vleet et al. 2001)
	3.44 ± 0.45 μM		(Konečný et al. 2007)		86 μM		(Patten and Koch 1995)
(S)-Flurbiprofen	16.2 ± 0.9 μM (Baculosomes)		(Kumar et al. 2006)	Chlorzoxazone	250-300 μM		(Hanioka et al. 2003)
	21.6 ± 0.8 μM (Supersomes)				69 μM	47 ± 10 μM	(Tassaneeyakul et al. 1993)
	120 ± 10 μM, 45 ± 2 μM (Reconstituted)				193 ± 28 μM		(Baranová et al. 2005)
	1.9 μM (HLM)		(Walsky and Obach 2004)		33 μM		(Chen et al. 1996)
	~33 μM		(Hutzler et al. 2003)		0.65 ± 0.08 mM		(Fairhead et al. 2005)
	1.9 ± 0.4 μM	4.7 μM	(Tracy et al. 1995)		50 μM		(Beckmann‐Knopp et al. 2000)
	~7 μM		(Youdim and Dodia 2010)		150 μM		(Howard et al. 2001)
	19.4 μM		(Tracy 2006)		660 ± 18 μM		(Shimada et al. 1999)
(S)-Warfarin	4.6 ± 0.3 μM (Baculosomes)		(Kumar et al. 2006)	Nitrosodimethylamine	212 μM		(Patten and Koch 1995)
	13 ± 2 μM (Supersomes)				36 μM		
	32 ± 2 μM, 52 ± 9 μM (Reconstituted)				66 μM		(Patten et al. 1992)
	3.7 μM (HLM)		(Hemeryck et al. 1999)		59 μM		
	4.1 ± 0.6 μM		(Rettie et al. 1992)		21.7 ± 1.5 μM		
	4.1 ± 0.9 μM		(Lang and Böcker 1995)		22.3 ± 0.6 μM		

Further, we analyzed the value of *K*_d_ (as determined by *in silico* binding and in vitro protein-solution binding) and compared it with experimental *K*_M_ (Table 20). We see little correlation between experimental *K*_d_ and experimental *K*_M_. (At times, a theoretical breach occurs when some experimental *K*_d_ values are larger than experimental *K*_M_ values, as seen in 3, 9, 10 & 12. Also, it is difficult to imagine a several folds higher experimental *K*_M_ in comparison to K_d_, as is seen in 2, 5, 8, 11.) Except for 9 & 12, there is little correlation between experimental *K*_d_ or experimental *K*_M_ with *in silicoK*_d_ (distal pocket docked). But, even in 9 & 12, we have a theoretical fallacy that experimental *K*_M_ is smaller than experimental *K*_d_. This cannot be disregarded merely as an inefficiency of docking programs. In the blind dockings, the *in silicoK*_d_ values are higher than the experimental *K*_d_. (Predicted solubility or log P values are not highly different from experimental findings.) There exists no correlation between residence time (as calculated from *in silicoK*_d_ or experimental *K*_d_ or experimental *K*_M_) with experimental conversion time. The magnitude of conversion time is higher than the residence times by 10^3^ (lowest limits as seen in 8 and 9) to 10^8^ (higher limits as seen in 3 and 12). These differences are not trivial or small and therefore, the “committed to catalysis” hypothesis must be rendered invalid. If we have to buy that proposal, we should also accept that the most inefficient or poor substrates have the highest binding efficiency to a given CYP! This cannot be supported with any *in silico* or experimental data anyway. (Further, it counteracts the very purpose for which the hypothesis was invented.) The overall conversion time is low (<10 seconds) for “smaller and leaner” molecules with relatively unobstructed reaction site and it is not low for tight binders (lower *K*_d_, whether *in silico* or experimental) or more reactive molecules/centres. This could show diffusion constraints in reaction kinetics.

**Table 20:** Comparison of in silico and in vitro reaction / binding parameters

S.No.	CYP	Substrate	Physical features (in silico)	ΔG (in silico)		*K*_d_ (in silico), (μM)		Res. time (in silico-calc) (μs)		Physical features (exp)	*K*_d_ (exp-ref)	*K*_M_ (exp-ref)	Res. time (exp-calc) (μs)		Conv. time (exp-ref) (s)
			Solubility, log p & log D	Distal pocket	Blind	Distal pocket	Blind	Distal pocket	Blind	Solubility, log p			From *K*_d_	From *K*_M_	
1	3A4	Testosterone	115 μM, 3.37 & 3.3	−6.61	−6.10	14	33	72	31	81 μM, 3.32	36 (Farooq and Roberts 2010)	100 (Yuan et al. 2002)	28	10	53 (Wang et al. 1997)
2		Erythromycin	625 μM, 2.60 & 1.55	−6.43	−4.48	19	508	53	2	2725 μM, 3.06	125 (Farooq and Roberts 2010)	78 (Wang et al. 1997)	8	13	545 (Wang et al. 1997)
3		Amitriptyline	16 μM, 4.81 & 2.8	−4.75	ND	348	ND	3	ND	35 μM, 4.92	178 (McLure et al. 2000)	67 (Eugster et al. 1993)	5.6	15	1091 (Venkatakrishnan et al. 2000)
4	2E1	Chlorzoxazone	17457 μM, 1.94 & 1.9	−5.49	−4.12	92	935	11	1.1	5898 μM, 1.6	ND	40 (Yuan et al. 2002)	ND	25	300 (Lee et al. 2006)
5		p-Nitrophenol	25879 μM, 1.61 & 1.28	−4.86	−5.67	267	68	3.7	15	83387 μM, 1.91	23 (Hartman et al. 2013)	197 (Duescher and Elfarra 1993)	44	5.1	375 (Duescher and Elfarra 1993)
6	2D6	Bufuralol	136 μM, 2.99 & 0.81	−6.13	−4.28	31	713	32	1.4	ND, 3.5	6.2 (Guengerich 2005)	10 (Yuan et al. 2002)	161	100	5.9 (Marcucci et al. 2002)
7	2C19	Omeprazole	1039 μM, 2.43 & 2.43	−6.51	−4.43	16	553	61	1.8	ND, 2.23	ND	3.7 (R) 8.2 (S) (Foti and Wahlstrom 2008)	ND	270 (R) 122 (S)	1.7 (R) 6.9 (S) (Foti and Wahlstrom 2008)
8	2C9	Flurbiprofen	102 μM, 3.94 & 1.38	−6.64	−5.73	13	61	76	16	33 μM, 4.16	0.13 (Hummel et al. 2008)	29 (Hutzler et al. 2001)	7692	35	4.8 (Hutzler et al. 2001)
9		Diclofenac	15 μM, 4.26 & 1.58	−6.39	−6.15	20	30	50	33	8 μM, 4.51	16 (Wester et al. 2003)	6 (Lewis et al. 1998)	63	167	0.7 (Liu et al. 2012)
10		Warfarin	153 μM, 2.74 & 1.34	−6.34	−5.72	22	62	46	16	55 μM, 2.7	8.2 (Takahashi et al. 1999)	5 (Yuan et al. 2002)	121	200	194 (Liu et al. 2012)
11	2A6	Coumarin	453 μM, 1.78 & 1.82	−6.33	−5.83	22	52	45	19	876 μM, 2.07	0.3 (Yano et al. 2005)	2.1 (Lewis and Dickins 2002)	3704	476	2000 (Li et al. 1997)
12	1A2	Ethoxyresorufin	ND, 2.28 & 2.42	−7.15	−5.96	5.5	42	181	24	ND, ND	5.3 (Lin et al. 2001)	1.7 (Lewis and Dickins 2002)	189	588	2609 (Eugster et al. 1993)

The in silico ΔG and *K*_d_ values are from our studies (except 3); *K*_d_ exp is from equilibrium dialysis or Soret differential spectral analysis- found in literature

*K*_M_ is any selected value reported for the enzyme-substrate combination in literature and conversion time is the time taken for one molecule of enzyme to convert one molecule of substrate to the specific product

The numbers in the large brackets in the last few columns are the pertinent references

## Discussion

### Establishing the need for this work

In the current work, we investigate the application of the “plastic crystal structure” (as available from pdb files deposited by other researchers) to afford insight into the purported “elastic dynamics” of CYP-substrate interactive mechanism. At the outset, we must state that exploring the dynamic interactions of a squishy “breathing” protein located in a semi-mobile phospholipid environment with a static protocol like molecular docking is not necessarily a very apt method. But then, one simple question continued to bother us- if the F and G loops move to accommodate all substrates of a given CYP within the heme distal site, why is it that only some substrates are reacted by the ‘high potential Compound I’ of a given CYP? Further, our zeal to undertake the current study was fueled by the curiosity generated upon noting the inadequacy of the erstwhile hypothesis. It is known that reaction dynamics of several CYPs do not show a classical Michaelis-Menten paradigm for initial kinetics under steady-state conditions (Atkins 2005). Furthermore, several aspects (as noted from literature) speak out against a mere heme-centred active-site hypothesis:

1. Quite interestingly, upon changing the reductant or by the introduction of Cyt. *b*_5_ / lipids (as per the erstwhile understanding, these components should have little influential scope to govern the substrate-CYP interactions), the *K*_M_ value changes significantly (Parashar et al. 2014). This was when *K*_M_ was calculated using non-linear regression with only the lower values of the substrates (that fit the Michaelis-Menten paradigm), giving a curve that apparently resembled a decent hyperbolic asymptote. Also, it is common knowledge to many CYP researchers that the overall stoichiometric yield varies with time and also by altering the components’ initial concentrations. Such observations cannot be explained if the kinetics is critically dependent on a tight enzyme-substrate complex formation (as the erstwhile hypothesis solicits).
2. Kinetic Isotope Effects (or KIEs) afford us key insight into the dynamics of substrate and enzyme (reactive) intermediate’s interactions. The presence of high intra-molecular KIEs (Miwa et al. 1980) in CYP-catalyzed reactions imply that there is little spatial constraints and the substrate is very free to approach the enzyme-generated catalytic intermediate. This finding means that the substrate is not tightly bound in a preferred orientation within the active site and is free to tumble around. (This intriguing result is particularly relevant for non-activated hydrocarbons, which are the classical reactions of high potential reactive intermediate of CYPs.) To account for this contradictory finding, some apologists of the erstwhile hypothesis have invoked upon a “committed to catalysis” idea (Lu 1998), which seeks that while the CYP is bound to the substrate to effect a change in redox potential, the substrate is yet free to rotate within the “active site”. The proposal flouts Occam’s razor because then, each CYP needs to have some kind of sensor(s) and processing intellect to effect this proposition. So, the erstwhile hypothesis does seek a case of mutually exclusive options (obligatorily bound and freely rotating substrate) at the same instant. [Let us remember that existence of intra- or inter- molecular KIEs in CYP reactions are not an indication that the reactions must occur within the heme distal pocket either.]
3. At times, the experimental *K*_i_ and *K*_d_ values of an inhibitor (Type I or Type II) for CYPs have been reported to be lower than the enzyme concentrations taken for assay (Locuson et al. 2004; Locuson et al. 2003; Collom et al. 2008). To a biochemist, inhibition of a biological process affords the most fundamental mechanistic and phenomenological insight regarding the crucial steps involved and therefore, this is a critical point that speaks against the erstwhile hypothesis.
4. Survey of substrate “preferences” of most mammalian P450s (for the hydroxylation reaction) do not give any clear cut ideas of active site topologies of a particular isoform. When a CYP can kinetically differentiate between an R and S enantiomer of a substrate (Rettie et al. 1992), it is highly unusual that most of the human liver microsomal hydroxylations are not enantioselective (i.e., do not give products with high enantiomeric excess). Also, in exceptional cases where enantioselective hydroxylations are observed, the reactions are not regioselective (Dayer et al. 1982). Hydroxylations of molecules at unactivated carbon atoms (in either aromatic or aliphatic substrates) seldom show any enantioselectivity. This is when oxygen insertion reactions by the same P450s for similar or much smaller substrates but with activated carbon or hetero atoms with relatively higher electron density at the reactive moiety (olefins giving epoxides, N- or S- atom containing substrates giving the oxides, substrates with a sterically unhindered benzylic carbon giving hydroxylated products etc.) showed varying levels of enantioselectivity, depending on the substrates. These observations do not quite go well with a “space filling - topography - reactive moiety” considerations for an active site process.
5. In most cases, the hydroxylation is always higher on the energetically favorable atom on the substrate (making CYP metabolic reactions highly predictable, (Jones et al. 2002; Korzekwa et al. 1990), and not the sterically favorable one. (There are exceptions to this trend. Yet, we deem this overwhelming pattern as a “clear tell” for the microsomal P450s’ reaction mechanism.)
6. Jones group’s work demonstrated that it is a diffusible radical species (t-butoxy) that best approximates CYP catalytic process in terms of KIEs (kinetic isotope effect) and LFEs (linear free energy relationships) when compared to a heme-centred Fe-pyr system (Manchester et al. 1997). This is a key mechanistic insight that cannot be ignored.

When considering the mechanistic scheme of CYPs, we deem spatio-temporal aspects to hold more critical importance. And when one solves a puzzle that liver microsomal CYPs are, the larger pieces have to be pieced in first. We considered a possibility that some of the observations (open-closed conformers, substrates-bound crystals and Type II spectra etc.) could be coincidental aspects, which may be inconsequential in the bigger picture. If we take the erstwhile “high affinity binding at heme pocket hypothesis” to be binding, then the endeavor of determining crystal structures would be relegated to lesser significance because dynamic aspects alone would determine reactivity. Under the purview of the erstwhile hypothesis, crystal structures of diverse CYPs do not show a discernible structure-function correlation. All CYPs’ structures must be “tinkered” by modeling/simulation to explain their reactivities, without really explaining their substrate preferences. In the light of the arguments above, considering our recent findings (as cited in the last part of the introduction) and the results presented in this study, we could indulge in an idea that the trigger of a molecule binding to the heme pocket need not be the sole sponsors that afford selectivity/specificity in CYP mediated redox reactions. With this supposition, we justify the evolutionary mandate of CYPs and the inherent crystal structure of a protein also gets its due significance. We also think that it is quite probable that a hydrophobic protein could be crystallized out in a number of ways, under varying conditions, in the presence or absence of some organic molecules. Whether these structures have functional relevance is to be decided by experimental/functional verification. Literature also shows that in most cases, a given microsomal CYP’s substrate is unable to induce the production of the same CYP (some substrates of CYP3A4 are notable exceptions). This showed that there was no biological/evolutionary link for a given drug molecule’s topology to the expression of the CYP that metabolized the same drug molecule. [Further, it is opportune to add a disclaimer that the example of P450BM3, a reductase-P450 hybrid, which gives very high rates and enantioselective hydroxylations (Farwell et al. 2015), may not be very appropriate for the liver microsomal CYP systems. In these systems, the distal pocket’s and the access channel’s amino acids’ role in substrate positioning/reactivity has been well-explored and documented (Ravichandran et al. 1993; Li and Poulos 1997). Here, the reduction of heme could be an intramolecular process, with facile electron tunneling and proton relay. In microsomal CYPs, the electron transfer is currently believed to be inter-protein, and that, over apparent distances of >15 Angstroms. This seems a rather unlikely proposal]. Therefore, we had several reasons to believe that our findings on the phenomenology involved in peroxidase system were very relevant herein, within microsomal CYPs. Therefore, the prima-facie case for thinking beyond the currently available paradigm is established.

### Critical dissection of the erstwhile hypothesis

The prevailing explanation for CYP catalysis (which seeks the localizing of the substrate at the distal heme pocket) solicits that-

(i.) the protein be highly flexible (high level of induced fit) and this flexibility can be exercised in several modalities (given the diverse topographies of its substrates), and
(ii.) each substrate should have a specific locus (or multiple loci) to bind on the protein surface or crypts therein. Further, this substrate-enzyme ‘binding event’ should serve as a molecular trigger for four obligatory outcomes -

- alteration of redox potential of the heme centre,
- “commitment of substrate to catalysis”- that is, the substrate should not dissociate through the subsequent steps of the catalytic cycle and must remain ‘bound and localized’ within the distal pocket,
- presentation of the substrate within suitable bonding distance at the heme centre, for oxygen rebound &
- a proton shuttle must deliver protons at the heme distal site for substrate hydroxylation.

This must be orchestrated in a precise sequence of events, as follows- 1. First, substrate should bind to the distal site of CYP and it should be concomitantly followed by CPR binding at the protein’s periphery. Then, a molecule of oxygen should diffuse into the distal pocket of the erstwhile complex, thereby giving yet another ternary or quaternary complex. All these fastidious requirements mock Occam’s razor and simple notions of probability. It is difficult to imagine how a single protein could/should evolve the “molecular intelligence” for the metabolism of diverse xenobiotics through a highly complex process, sans chemical or biological logic. From data given in this work (particularly, Tables 2, 3, and 4), one would challenge the very premise of the requisites sought by the erstwhile paradigm. This is because the crystal structure shows that a CYP like 2E1 does not have any channels leading into the distal pocket. Other CYPs have some channels but most of them have too small dimensions (the substrate molecular dimension is at least twice the constrictions of the channels within the protein) afford any probability for a direct diffusion of the substrate molecule to “the locus of catalysis” (the distal heme centre). Further, some substrates are too large to be even accommodated at the active site itself, even if it were to open up in a reasonable way.

But apologists of the erstwhile hypothesis could argue that the classical model of P450_Cam_ also shows no cavity, and yet, it is very well known that camphor does show a Type I binding in the distal heme pocket. Therefore, if the soluble protein of P450_cam_ is “flexible” enough to allow movement of camphor into the distal pocket, the membrane proteins like microsomal CYPs can also be envisaged to be flexible. Though membrane proteins are seen as ‘squishy’ proteins, they are also known to bind ligands with high specificity, thereby indicating a high level of surface plasticity. Very importantly, the hydrophobic phospholipid microenvironment can be imagined to be a constrained and low-energy microcosm, which is only semi-fluidic. The distal pocket may be large in many CYPs but access to it is definitely constrained. To imagine that small amounts of diverse substrates could repeatedly find the probability to - (i) bind outside and get transported to the inside or (ii) squeeze their way in or (iii) the enzyme would welcome the molecules by opening and closing up through some undefined logic - is seeking repeated sequence of events of low probability (with respect to molecular dynamics). Even if some of these large molecules gathered access to the heme floor, we envisage that it is disadvantageous for a direct oxygen transfer to any hindered locus within framework of a rigid substrate, within the constrained heme distal pocket. We speculated that it may not be mere coincidence that microsomal CYPs’ substrate selectivity or docking orientations do not correlate with reactivity or yield.

For the enzyme catalyzed reaction (where E is enzyme, S is substrate and P is the product)-

$$ \mathrm{E} + \mathrm{S}\ \leftrightarrow\ \mathrm{E}\mathrm{S} - - \mathrm{E}\mathrm{P}\ \to\ \mathrm{E} + \mathrm{P} $$

let us assume that k_1_ and k_−1_ are the forward reaction rate constant to form ES complex and the backward breakdown rate constant of ES, respectively; and k_2_ is the forward breakdown rate constant of EP complex. Then,

$$ {\mathrm{k}}_1 = \left(\mathrm{d}\left[\mathrm{E}\mathrm{S}\right]\ /\ \mathrm{d}\mathrm{t}\right)\ .\ \left(1/\ \left[\mathrm{E}\right]\left[\mathrm{S}\right]\right) $$

Theoretically, for nM levels of CYPs and μM levels of substrate (the usual working ranges for in vitro studies), we can have only < nM ranges of [ES] formed in > milliseconds (which is the generally accepted “breathing time” of proteins, the time that would be required for F/G loops/helices to move around). So, the k_1_ for this reaction alone can be lower than 10^7^ M^−1^ s^−1^ or be up to the theoretical maximum of 10^9^ M^−1^ s^−1^. Experimentally, nM levels of CYPs give product formation with pseudo first order rates equalling 1 per second (for efficiently coupled baculosome systems in vitro). For such systems, the overall reaction cycle’s second order rate constant achieves diffusion limitations for this single step alone. So, the diffusional constraints and probabilistic barricades involved in an ordered (sequential) reaction of multimolecular collisions/complexations of at least five components (nM levels of CYP & CPR and micromolar levels of oxygen, substrate & NADPH) are unaccounted for. Quite simply, under the purview of the erstwhile hypothesis, collision/complexation frequencies of bulky hydrophobic proteins and drug molecules cannot achieve such super-concerted orchestration of events (that exceed diffusion limitations) at the phospholipid interface. This ‘kinetic’ argument is the most compelling quantitative logic against the erstwhile hypothesis.

### A new hypothesis, its stimulus and crux

**Fig. 2:**
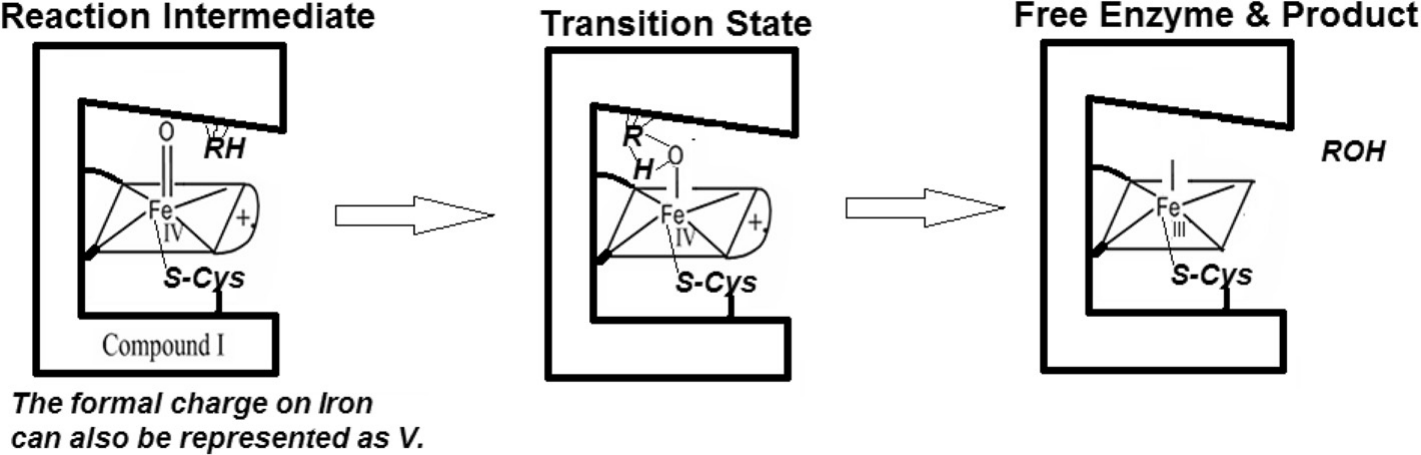
The erstwhile mechanism of binding of substrate at a distinct locus (distant from heme centre) and oxygen rebound at heme centre: The erstwhile mechanism seeks (i) binding of the substrate to the heme distal pocket, (ii) the generation of a localized two-electron deficient reactive intermediate at the heme centre, (iii) transposition/translocation of the substrate within bonding distance in the transition state and (iv) dissociation of the product and outward diffusion of the same owing to lower binding affinity (after the completion of reaction)

Recently, we have argued that the efficient Type II binding of a ligand at the heme distal pocket is seen only at high concentrations of the enzyme (into micromolar ranges) and at very high ligand pressures (Parashar et al. 2014). The same logic would be held viable for Type I bindings too. In physiological conditions, it is highly unlikely that such high concentrations of enzymes and xenobiotic substrates ever occur, for the “P450_cam_ type logic” to be of any functional significance. Further, in our lab studies, we couldn’t note any significant spectral change (a hypsochromic shift of Soret spectra or the emergence of the high spin signature band at 650 nm) for some CYPs that we checked (CYP2C9-Diclofenac and CYP2E1-Chlorzoxazone; at micromolar level of the enzyme and a ligand pressure of 1:100). Also, it is difficult to imagine that diverse molecules of varying topography and electrostatics could bind efficiently to the very same loci in the distal pocket or near the channel to be transported to the distal site. A simple pictorial understanding of the erstwhile paradigm is shown in Fig. 2. The erstwhile hypothesis requires that in the transition state, the heme-Fe-O species is within bonding distance of both C and H atoms of the substrate. In the extensive dockings we carried out and from several crystal structures of CYPs available till date, one cannot find any evidence for this requisite. On the other hand, if approach or binding of substrate to a protein surface or crypt served as a molecular trigger [as seen in the well-known and explicable example of some lipases where a flap opens up upon the presence of a hydrophobic substrate (Grochulski et al. 1993)], then the different loci for diverse substrate binding should be mobile and engineered to swing around and position the bound substrate at heme centre. Quite simply, this is asking for too much “intelligence” from a simple protein molecule. We indulged even this proposition but could not find any evidence to this effect (Tables 2 and 3). Further, the data from sections 8 through 10 of results presented above give very convincing arguments against the erstwhile hypothesis.

We propose the following probabilistic phenomenological hypothesis, which not only explains most of the experimental observations and *in silico* findings, but also justifies Occam’s razor’s criteria. (This is, albeit, at the aesthetic expense of tolerating a “radical” hypothesis.) The essential aspects of the ‘radical’ hypothesis, which is already introduced in our earlier works, (Gideon et al. 2012; Manoj et al. 2010a; Manoj et al. 2010b; Parashar et al. 2014) are as follows- CPR produces diffusible species that serve as one-electron equivalents (like superoxide), which bind with CYP and get stabilized. These catalytic species (whether bound or free) thereafter convert the substrates. In this hypothesis, the heme centre can be reduced or activated in three different ways-

(i.) One-electron equivalents generated by CPR can be relayed to the heme centre through diffusible species, via the proximal thiolate (and a molecule of oxygen could subsequently bind to the distal heme centre). A survey of the prominent microsomal CYPs shows that all these enzymes have a readily solvent accessible proximal site (Additional file 1A, Figure A1P), which makes this process very facile.
(ii.) The presence of a suitable substrate could reduce the heme centre on its own merit (and a molecule of oxygen could subsequently bind to the distal heme centre).
(iii.) Superoxide or hydroxyl radicals generated in situ could bind to the distal heme centre by diffusion through the distal channel.

**Fig. 3:**
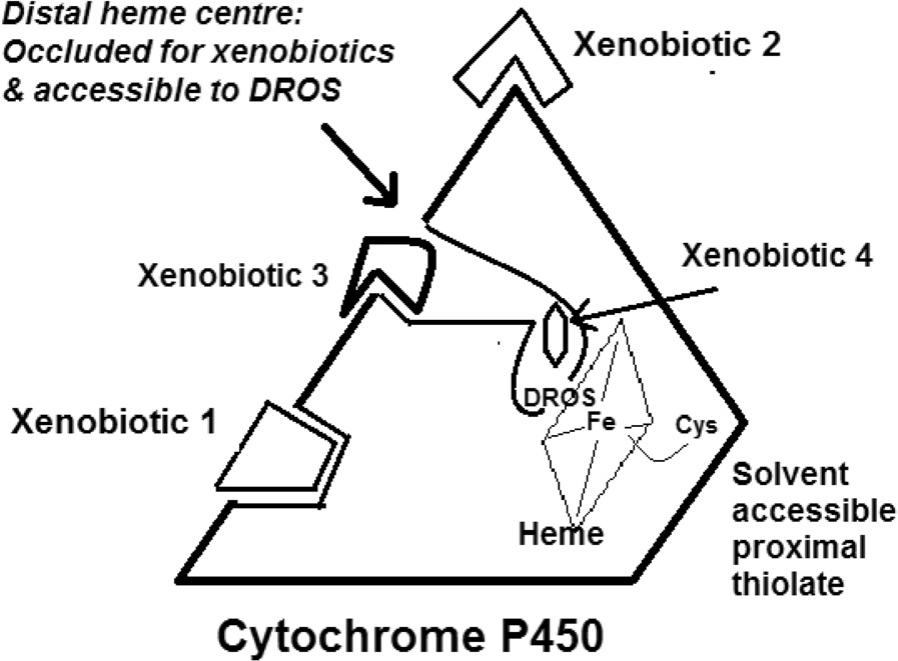
Interactions of various xenobiotics and DROS with microsomal CYPs: There are distinct binding sites for the two substrates (DROS and xenobiotics) and these loci are not brought together by an induced fit, for a direct bond-formation (between the two substrates) in the transition state. There exists an uncertainty regarding the exact locus where the two substrates finally interact/react, as a probabilistic fate governs the outcome. The xenobiotic substrate 4 could go through the oxidation at/near the heme centre and such reactions would be favored at high enzyme:ligand concentrations/ratios. Other substrates would have better probability of being reacted by the given CYP away from the heme centre. This may be owing to better binding per se (as in substrate 1) or/and because of a more probable presentation to the reactive species as it emerges out from the distal heme pocket (as in substrate 3). The reactivity of a molecule like two would be dependent on several factors

The outcomes of all the three steps above would be the same. Thus, if we indulged a hypothesis that the hydrophobic distal pocket of CYP hemes could serve as “a residence/stabilization zone” for reactive species (like hydroxyl or superoxide radicals), then the functional lifetime of the same increases significantly. This supposition is justified by a pioneering paper published in the late 1970s (Blumenthal and Kassner 1979). While probing the binding of azide to a model hemoprotein, they had concluded that enhanced polarity of the heme distal pocket confers oxygen binding (and auto-oxidation of heme) whereas the anion binding and its stabilization is favored in the hydrophobic heme pockets. Therefore, if the CYP is not present in the vicinity to stabilize the diffusible reactive species, the one-electron equivalents diffuse out into the aqueous phase and are lost to water formation. If CYPs are present and if the substrate is bound on the surface (or even the heme distal pocket or any other crypts therein) of the CYP and available nearby, the probability of reaction goes up very highly. The rate enhancement is neither through a one site “lock & key” mechanism (alone) nor through an “induced fit”. The enzyme enhances rates by stabilizing the diffusible reactive intermediate and by enhancing the probability of the reactive intermediate to find the substrate in/around its vicinity. Therefore, there exists a definite “uncertainty” with respect to the exact locus of interaction of the substrate and reactive intermediate. To the best of our knowledge, such a way of enzyme functioning is not yet advocated. Therefore, we coin our proposal as ‘*murburn*’ hypothesis (“mured burning”- connoting a mild and unrestrained burning in a confined microenvironment) and such macromolecules as *murzymes* (**m**ediating **u**nrestrained **r**edox catalysis). The representation in Fig. 3 captures the essential scheme for interaction of various xenobiotic substrates with CYPs. In comparison with the erstwhile hypothesis (shown in Fig. 2), the new hypothesis does not seek a high affinity binding or translocation into the deep-seated heme pocket for a direct heme Fe-O to drug molecule bond formation step. Nanomolar concentrations of diffusible radicals could be envisaged to have effective diffusion radius of several Angstroms (even up to nano-scales), given the hydrophobic pocket of CYPs. The reaction mediated in such a scenario can be nonspecific or regiospecific and even be enantioselective. The case must be noted that most CYPs hydroxylate diverse drugs at the most energetically favorable loci within the substrate molecule (and not the most spatially open centre). This specificity can also be achieved in suitable chemical controls in enzyme reactions that involve diffusible species ((Manoj 2006) and our unpublished work). Also, if the hydroxyl moiety incorporation was completed in the heme distal pocket at the heme centre, then we cannot explain how a bevy of substrates get activated by additives (Hutzler et al. 2001; Kenworthy et al. 1999) or why some substrates are not metabolized by some CYPs. Multiple molecules binding at the heme centre in a dynamic state would be a low probability event (though one gets crystal structures of these types of complexes from highly concentrated systems). We have already explained these findings for heme-peroxidases (Andrew et al. 2011; Gade et al. 2012; Parashar and Manoj 2012) and the same explanations could hold well here too.

### Application of ‘murburn’ hypothesis to explain kinetics and mechanism

If we continue with the discussion on the Michaelis-Menten supposition (from the earlier section above)-

1 $$ {\mathrm{K}}_{\mathrm{M}} = \left({\mathrm{k}}_{-1} + {\mathrm{k}}_2\right)\ /\ \left({\mathrm{k}}_1\right) $$

As per laws of equilibrium-

2 $$ {\mathrm{K}}_{\mathrm{d}} = \left({\mathrm{k}}_{-1}\right)/\left({\mathrm{k}}_1\right) $$

It is clear that-

3 $$ {\mathrm{K}}_{\mathrm{M}} = {\mathrm{K}}_{\mathrm{d}} + \left({\mathrm{k}}_2/{\mathrm{k}}_1\right) $$

Therefore,

4 $$ {\mathrm{K}}_{\mathrm{M}} \sim {\mathrm{K}}_{\mathrm{d}},\ \mathrm{when}\ {\mathrm{k}}_{-1}\kern0.5em > > {\mathrm{k}}_2 $$

and/or

5 $$ {\mathrm{K}}_{\mathrm{M}} \sim {\mathrm{K}}_{\mathrm{d}},\ \mathrm{when}\ \left({\mathrm{k}}_2/{\mathrm{k}}_1\right) = 0 $$

Now, the erstwhile hypothesis assumes that once the substrate is hydroxylated, it loses efficiency for the enzyme. Therefore, the forward breakdown of EP may be comparable or efficient with respect to the backward breakdown of ES. In this case, the contribution of k_2_ cannot be neglected. Therefore, we might have a higher value of *K*_M_ than that of *K*_d_. But under any circumstances, the value of K_M_ cannot be lower than that of *K*_d_. If one were to accept the ideas advocated by Guengerich group, most microsomal P450-substrate forward binding is very fast and only diffusion limited (k_1_ ~ 10^9^ M^−1^ s^−1^). If that were true, it is highly unlikely that the physical value of k_2_ can approach k_1_ and therefore, supposition (e) tends to be the applicable scenario (as is true for many enzymes in normal environments). Under such scenarios, the data in Table 20 cannot be explained by the erstwhile hypothesis. The new hypothesis offers the following reasons for the same-

1. *Experimental K*_*M*_: It is a reflection of multiple events: (a) binding of substrate with enzyme, (b) production and binding of diffusible radicals with heme centre, (c) reaction of diffusible radicals with substrate & (d) competition of half-reacted substrate (intermediates) with other reactive species within milieu. If we imagine such a scenario, we can explain the poor correlation of *K*_d_ and *K*_M_. With the new hypothesis, we can also account for the changes in *K*_M_ values under various setups of the same CYP and substrate combination.
2. *Conversion time*: It is an index of the overall processes listed in 1 above. It is not an index of “committed to catalysis” or binding of substrate to heme pocket at all.

[Besides the above, there could be systemic errors in the protocols employed in P450 research with- a. Experimental K_d_ determination by Soret differential spectrum: The Type I binding could be mistaken for heme reduction, both of which are associated with a similar Soret change. (Only the high spin marker at 650 nm is conclusive of Type I binding associated spin shifts for FeIII heme thiolates.) Further, the binding of hundreds of micromolar substrates to micromolar enzyme is not a good measure of the reaction environment (wherein, nM levels of CYPs have very little probability to bind to micromolar levels of substrates). b. Experimental *K*_d_ determination by equilibrium dialysis: Non specific bindings on to microsomal and other membranes would give higher values. c. *In silicoK*_d_ determination by heme pocket centered docking: Binding of a small organic molecule with a hydrophobic pocket gives similar ΔG values for a bevy of substrates and non-substrates with CYPs. This binding cannot be taken as an index of substrate’s affinity to the heme pocket. In many cases, actual access to the cavity may not be available on the kinetically relevant time scale.]

Our study implies that binding and reaction of the substrate at the heme-centre [as exemplified and sought by oxygen rebound from Compound I, (Groves 1985)] may not be an obligatory requirement for many CYPs-substrates combinations. (However, the study does not rule out the possibility either. Particularly, at high concentrations of enzymes (more than micromolar levels) and substrates (hundreds of micromolar levels), heme pocket binding of small molecules would be a relatively high probability event (Parashar et al. 2014). We would also like to add a disclaimer here that the hydrogen atom abstraction mechanism is not being challenged.) One wonders how the constrained and hydrophobic distal sites of CYPs afford an efficient proton shuttle in the phospholipid microenvironment. The pK_a_ of the substrate’s alkyl/aromatic hydrogens are way higher to be of any relevance (and that of the corresponding product’s hydroxyl protons are several units higher than reaction pH). The physiological/reaction pH affords protons at a meagre concentration of only ~40 nM. An analysis of the active site and cavities/channels of microsomal CYPs shows clearly that this requirement sought by the erstwhile paradigm is stretching Occam’s razor criteria (Tables 2 and 3). Based on our works till date, the reaction scheme of the CYP + CPR system could be minimally represented now as-

6 $$ 2{\mathrm{O}}_2 + \mathrm{N}\mathrm{A}\mathrm{D}\left(\mathrm{P}\right)\mathrm{H}\ \to\ {{2\mathrm{O}}_2}^{*-} + \mathrm{N}\mathrm{A}\mathrm{D}{\left(\mathrm{P}\right)}^{+} + {\mathrm{H}}^{+}\left(\mathrm{P}\mathrm{rimarily},\ \mathrm{C}\mathrm{P}\mathrm{R}'\mathrm{s}\ \mathrm{role}\right) $$

7 $$ \mathrm{R}\mathrm{X} + {{\mathrm{O}}_2}^{*-} + {\mathrm{H}}^{+}\to\ \mathrm{R}\mathrm{O}\mathrm{H} + {\mathrm{O}\mathrm{X}}^{*}\left(\mathrm{Primarily},\ \mathrm{C}\mathrm{Y}\mathrm{P}\hbox{'}\mathrm{s}\ /\ \mathrm{milieu}'\mathrm{s}\ \mathrm{roles}\right) $$

8 $$ {{\mathrm{O}}_2}^{*-}/\ {\mathrm{O}\mathrm{X}}^{*} + {{\mathrm{O}}_2}^{*-}/{\mathrm{O}\mathrm{X}}^{*}/\mathrm{R}\mathrm{H}\ /\ {\mathrm{O}\mathrm{H}}^{-}/\ \mathrm{N}\mathrm{A}\mathrm{D}\left(\mathrm{P}\right)\mathrm{H}\ /\ {\mathrm{H}}_2{\mathrm{O}}_2/\ \mathrm{Enzymes}\ \to \left(\mathrm{Diverse}\ \mathrm{fates}\right) $$

9 $$ \mathrm{R}\mathrm{X} + {\mathrm{O}}_2 + \mathrm{N}\mathrm{A}\mathrm{D}\left(\mathrm{P}\right)\mathrm{H}\ \to\ \mathrm{R}\mathrm{O}\mathrm{H} + \mathrm{N}\mathrm{A}\mathrm{D}{\left(\mathrm{P}\right)}^{+} + {\mathrm{O}\mathrm{X}}^{-}\ \left(\mathrm{Overall}\right) $$

The first three equations depict the essential initiation process and the multiple competitions at later stages. Now, we can understand why experimentally determined *K*_M_ values show wide variations across reaction setups. The interaction of reactive species with the substrate is not captured or limited to its (substrate’s) binding of CYP at a particular site, but is more about its presentation in the relatively complex reaction scheme. The overall equation (9) shows the macroscopic thermodynamic drive at the phospholipid interface. That is- the uncharged hydrophobic molecules react to give ionic and hydrophilic species. This eventuality, in turn, serves as driving force for inundation and washing away by water molecules. As seen from equation (9), the system does not require extraneous protons. Till date, there exists no evidence for the consumption of a solvent proton at the heme centre. There is only proof for the incorporation of extraneous hydrogen atom into the substrate and this step need not occur at the heme centre in CYPs. CPR’s activity generates protons and radicals in the microenvironment. CYPs stabilize radicals generated and the event of oxygen and/or hydrogen atom insertion into the substrate could occur even outside the heme distal pocket. The rapid reactions of radical species would serve to pull the redox equilibrium to the right, and this is achieved by substrate oxidation (more efficient) or depletion of ROS (less efficient). The process is essentially constitutive and the coinage of *murburn* hypothesis signifies the radical process involving oxygen in the vicinity of heme (or other such redox) enzymes. The relevance of such a process in liver cells (and within cellular membrane interfaces in general) and its mandate in evolution needs to be probed further. Most importantly, the hypothesis makes a lot of kinetic sense. At an instance, nM levels of CYP/CPR could stabilize suprananomolar levels of highly mobile ROS/radicals within milieu (and this process is not ordered). The reaction of these intermediates with micromolar levels of drug molecules can explain the overall kinetics.

The new hypothesis is not countered by the fact that mutations of key amino acids in the heme distal pocket could deleteriously or positively affect activity (Butler et al. 2013; Hamdane et al. 2008; de Montellano 2015). This effect need not be brought about by substrate-binding alone, but it can also be owing to altered ROS dynamics (Gideon et al. 2012). If we could think of “out of the active site” solutions to explain the phenomenology of CYP reaction chemistry, there is ample scope for the *murburn* concept to explain most aspects of CYP reactivity. Very importantly, the new hypothesis justifies several key mechanistic findings and proposals from Hollenberg’s and Newcomb’s groups (Lin et al. 2012; Zhang et al. 2011; Zhang et al. 2013; Sheng et al. 2009; Newcomb et al. 2003a; Newcomb et al. 2003b), regarding-

(i.) mechanism-based inactivations: It was observed that loss of activity resulted owing to the covalent modifications of CYPs’ surface amino acid residues, by substrate molecules like Bergamottin, Clopidogrel, ethynylphenanthrene etc. and
(ii.) presence of multiple electrophilic reactive species in the CYP reaction milieu.

**Fig. 4:**
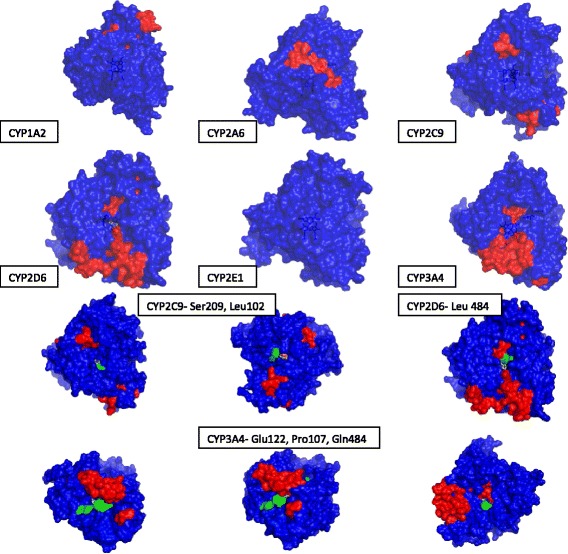
Visualization of channels leading to the distal pocket in the major CYPs (from the distal surface). The first two rows have the distal view with the heme falling on the plane of the paper and the images have been rendered 20 % transparent, to show the position of the deep seated heme (*in deep blue*). The highly continuous hydrophobic helices have been marked red. Further, an amino acid lining the access channel (if any) is also marked. The lower rows show the structures of the major CYPs. The amino acids marked with green are salient ones that mark the entry to the channel(s) leading to the distal pocket, as seen from different angles. In these images, the heme can be visualized through the channel, as yellow stick frames

The new hypothesis could justify the KIEs and LFEs obtained in several P450 reactions. A substrate bound on a surface crypt or even freely tumbling around near the enzyme could be subjected to catalysis by a diffusible reactive intermediate. The propensity and differentiated reactivity of very low amounts of this agent’s interaction with various moieties of a substrate could explain the extrinsic and intrinsic KIEs (Manchester et al. 1997) and provides room for multiple reactant species (Newcomb et al. 2003b; Coon 2003), which address concerns like- “the Fe-Oxo species does not have the required potential to activate some substrates”. Further, the new concept affords a greater scope for explaining the activation/inhibition of substrate catalysis by diverse additives. The new hypothesis can also explain how constrained loci of the substrate molecules could get hydroxylated when more open carbon atoms are left untouched by the highly reactive intermediate. Depiction of the overall topographies and distal surfaces of the major CYPs are shown in Figure A1Q of Additional file 1A. CYP3A4, with three channels and a large heme distal pocket is the most proficient of all CYPs (followed by CYP2D6 and CYP2C9) (as seen in Fig. 4). Coupled with its highly hydrophobic surface (to which diverse xenobiotic substrates can transiently bind) adjacent to the channel openings, the probability of the reactive intermediate meeting a substrate molecule in the vicinity of the distal pocket goes up significantly. (The large volume of CYP3A4’s distal pocket is not enough to explain its versatility because CYP2C9 also has a comparable hydrophobic voluminous pocket. CYP2D6 (with a relatively straight and wide channel) could allow small molecules’ enantioselective oxidations within the distal pocket, as is seen for benzylic hydroxylation of bufuralol. CYP2E1, without a channel that leads to the distal pocket, is a special case. It can be seen that this system has excess accumulation of ROS species and obligatorily requires Cyt. *b*_5_. (This scenario is akin to P450_cam_ needing putidaredoxin for effective activity. This may be because the probability for the ROS to bind at the heme centre via distal side goes down significantly.) Perhaps, the proximal side could also be involved in some reaction modalities. A specific antibody knocks out a given CYP’s activity because the antibody would bind to the surface region where the substrate would preferably bind. A strongly Type II binding substrate (which shows similar *in silico* and X-ray structures; like the large azoles) could potentially bind effectively to the heme centre with F & G loop opening at high enzyme-inhibitor concentration. Otherwise, they would be efficiently metabolized by CYPs because the probability of heme access of a large molecule would be lower when compared to their metabolism outside (Andrew et al. 2011; Manoj 2006; Manoj and Hager 2008). This is the reason why a molecule like Itraconazole is metabolized by CYP3A4. The pseudohyperbolic asymptotes (hitherto taken as Michaelis-Menten curves) are an index of the reactive intermediate’s interaction with the substrate. Depending on the reaction microenvironment (partitioning effects) and redox status therein, the curves can be affected significantly. This is why lipids, CPR/Cyt. *b*_5_ amounts etc. can alter the values of the pseudo *K*_M_ obtained. The very low *K*_i_ values obtained at times are an index of the radical formation and stabilization at low concentrations of the enzyme/additive. Larger molecules needn’t get into the distal pocket the through narrow or non-existent channels. Earlier, it was difficult to answer the question: Why is it that a given highly reactive intermediate of a promiscuous CYP (which could accommodate a given or several small molecule(s) in its active site) not observed to experimentally catalyze the given small molecule substrate? Now, it is understood that binding at an alternate and more topologically preferred location would be needed to enhance the probability of the substrate meeting the reactive diffusible intermediate. Protons are not needed at the heme centre with the new hypothesis. Hydroxylations caused by a diffusible radical species tend to be non-enantioselective and non-regiospecific. Both scenarios can change if the radical catches a bound substrate in a particular enantiotopic presentation, while it emerges from the heme centre or if the substrate is small enough and could gather significant access into the distal channel. The kinetic preference of a particular substrate enantiomer (without affording enantioselectivity of the product) is perfectly agreeable with the new hypothesis. Reactivity of the molecules themselves and presentations within or outside the heme distal pockets could be reasons for differences in activity within a given class of drug molecules. Most importantly, drug-drug interactions and genetic predisposition to drugs can be better explained by the new hypothesis. Also, since the new perception does not solicit a tight binding, products could be further oxidized without the necessity of being bound within the heme distal pocket.

### Projections to ratify the new hypothesis

1. A simple way to positively test the *murburn* hypothesis is to look for reactivity of several CYPs towards a non-substrate with the introduction of a substrate (of suitable redox potential) and vice-versa (and noting enhancement/inhibition if any). That is- a non-substrate could get converted if a suitable substrate is added into milieu. The activity towards a substrate could go up or get lowered if a non-substrate is introduced into milieu. The newly proposed hypothesis predicts a two step, one-electron route (whereas the erstwhile hypothesis is supposed to proceed via a single step, two-electron route) and workers should be able to ratify our prediction upon suitable selection of the CYP/substrate/modulator and tailoring of reaction environment. 2. Another way to test for the new hypothesis (or negate the erstwhile hypothesis) is- Small drug molecules that do not serve as “naturally” functional substrates could be presented to CYPs (for example- Chlorzoxazone to CYP2C9 and Flurbiprofen to CYP2E1) and the proteins allowed to crystallize. If we obtain crystals of the protein with the “non-substrate” drug molecule within the hydrophobic pocket, it must mean that this CYP structure (and the processes that led the substrate docking at the hydrophobic distal pocket) has little functional significance. 3. Enantiomeric excess, yield/specificity and product formation rates could be studied for a variety of molecules (with both activated and non-activated carbon as reaction centres; keeping the functionality of the reactive moiety constant) by- (a) increasing size of the molecule by substitutions away from the reaction centre (to probe the general diffusion constraints) & (b) introducing substitutions adjacent to the reactive centre (to see if reactivity occurs at the heme floor)- to give insights on the uncertainties involved. 4. The *murburn* hypothesis seeks obligatory role of diffusible radicals and ROS in the reaction scheme. Therefore, we should observe- (i) a temporal change in reaction stoichiometry/coupling (dynamics of ROS and competitions thereof) by varying the reaction components’ compositions/concentrations. (ii) Suitable one-electron scavenging small molecules, ions or enzymes should possess the ability to modulate/inhibit CYP/CPR reactions. (iii) It should be possible to experimentally simulate CYP reactions without CPR complexations (with ROS species alone or with non-specific redox partners and substrates). (iv) It should be possible to approach the reaction paradigm in CYP chemistry with suitable chemical controls. Also, chemical (non-enzymatic) controls should give enhanced specificity/rates upon reaction system modification (along the lines shown in Manoj 2006) for the hydroxylation reaction. 5. Modulations of reaction outcome/efficiency may be noted to various levels by changing the concentrations of CYPs/CPR and other small molecule redox-active additives (by virtue of affecting the competing reactions). 6. Most importantly- the erstwhile theory solicits that substrate binding to CYP is obligatorily required for the latter to receive electrons from CPR, leading to activation of molecular oxygen at heme centre. This supposition could be tested with simple reaction controls lacking the substrate (in diverse CYP-substrate setups) and checking for ROS in milieu.

## Conclusions

We have already demonstrated earlier that catalysis mediated by enzyme sponsored diffusible species can be very specific (regarding the preference of substrates, location of action within the substrate etc.) and projected the importance of the same (Manoj 2006). In those reactions also, the reactivity was afforded at the most energetically favored locus, quite akin to CYPs. So, it is seen that the CPO model is quite relevant to CYPs, in terms of some aspects of the overall phenomenology. Now, ROS in CYP reactions need not be seen as a result of an inefficient enzyme or inefficient substrate (or their combination, together with “badly chosen” or “unoptimized” reaction parameters) alone. Earlier, the binding of substrate molecules within the active site was supposed to account for and/or prevent such eventualities. This is now seen to be a redundant concept. The liver microsomes have no way of getting to know what substrates they would meet. So, the evolutionary agenda is addressed by the combination of CYP-CPR-NADPH-diradical oxygen (in lipid microenvironment). Herein, we have provided meta-analyses, arguments and extensive ‘*in silico*’ findings based on currently available docking programs (which use scoring functions for spatial complementarities, electrostatics/molecular force fields) to support the deductions that-

(1)Well-defined spatio-temporal constraints are not very evident in several CYP reactions. This finding is highly unlike a phenomenon occurring at occluded centres such as the heme distal site. Therefore, the heme distal pocket may be relatively passive in ‘interactions’ with several xenobiotic substrates. (2) The newly proposed *murburn* hypothesis explains key experimental and theoretical aspects of CYP reaction system.

## Additional file

Additional file 1:
**Supplementary information.** (PDF 6122 kb)
