# Supplementary material for: Functioning of drug-metabolizing microsomal cytochrome P450s: In silico probing of proteins suggests that the distal heme ‘active site’ pocket plays a relatively ‘passive role’ in some enzyme-substrate interactions
Source: In Silico Pharmacol. 2016 Feb 19;4:2. doi: 10.1186/s40203-016-0016-7 (PMC4760962; doi:10.1186/s40203-016-0016-7)
Supplement: Additional file 1: — Supplementary information. (PDF 6122 kb) [file 40203_2016_16_MOESM1_ESM.pdf]

Additional File A1

Figure A1A – 1: Big Substrates

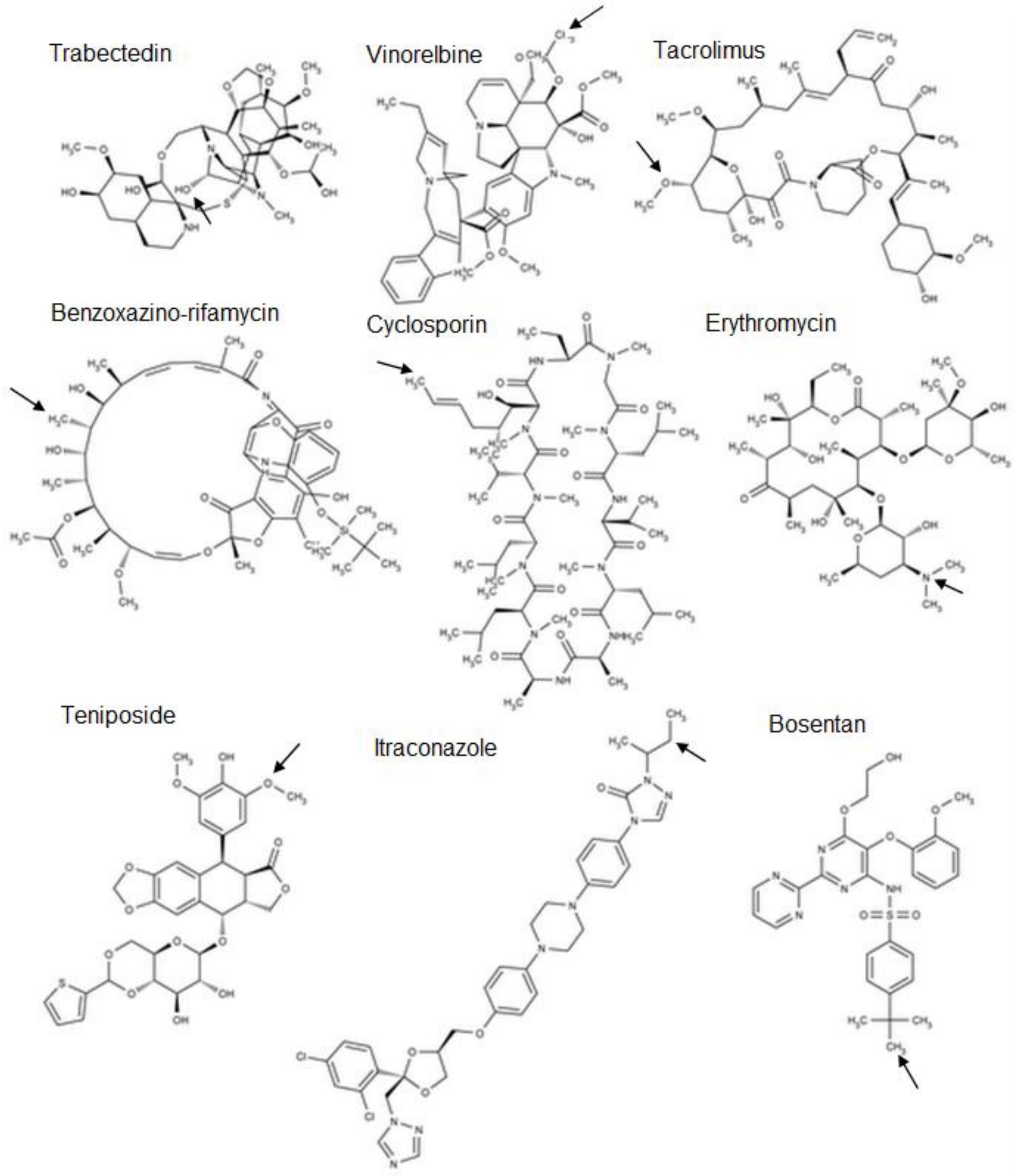

Figure A1A – 2: Big Substrates

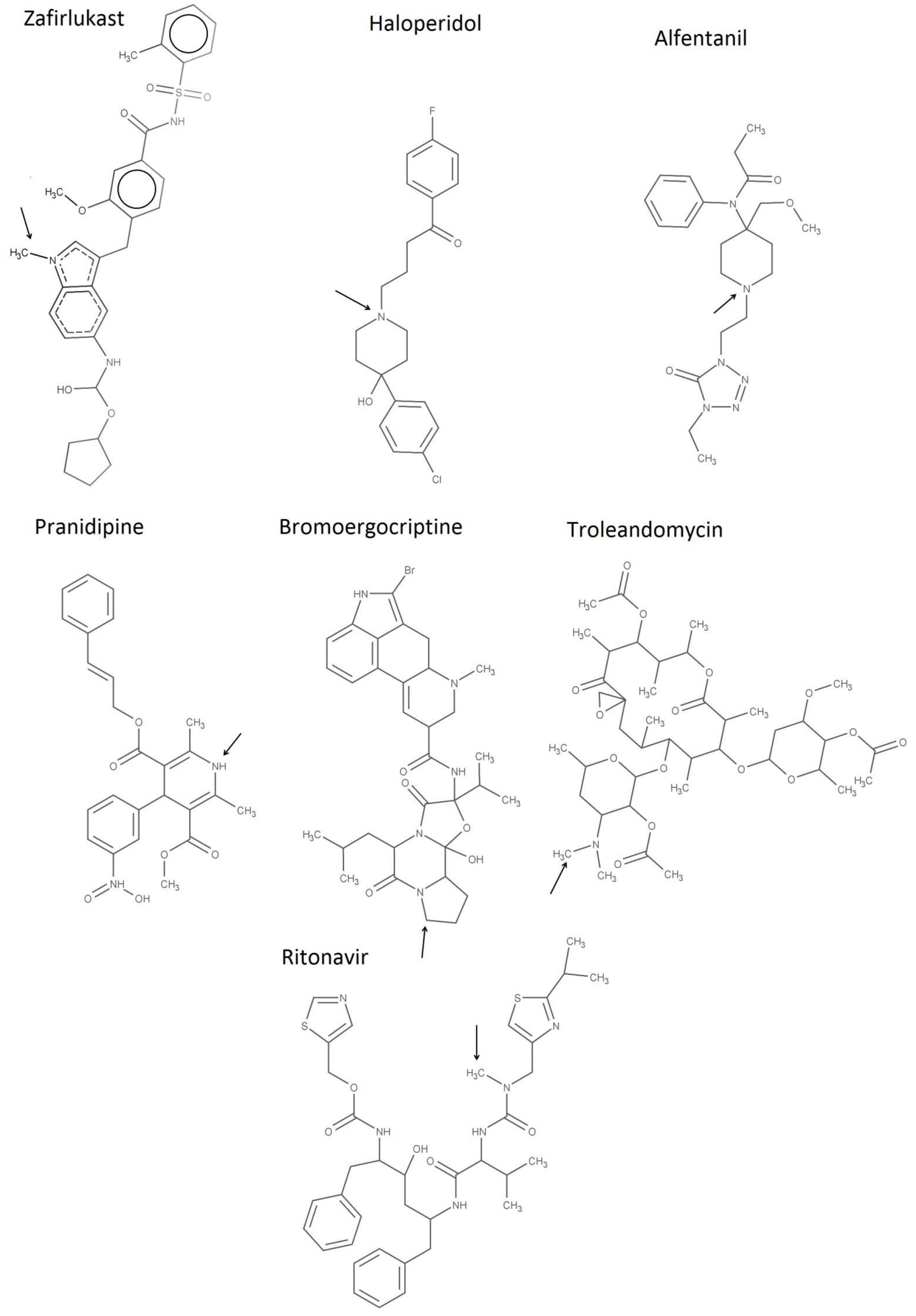

Figure A1B: Probing of binding of high affinity substrates or ligands to enzymes/proteins

1. FAB – Hapten

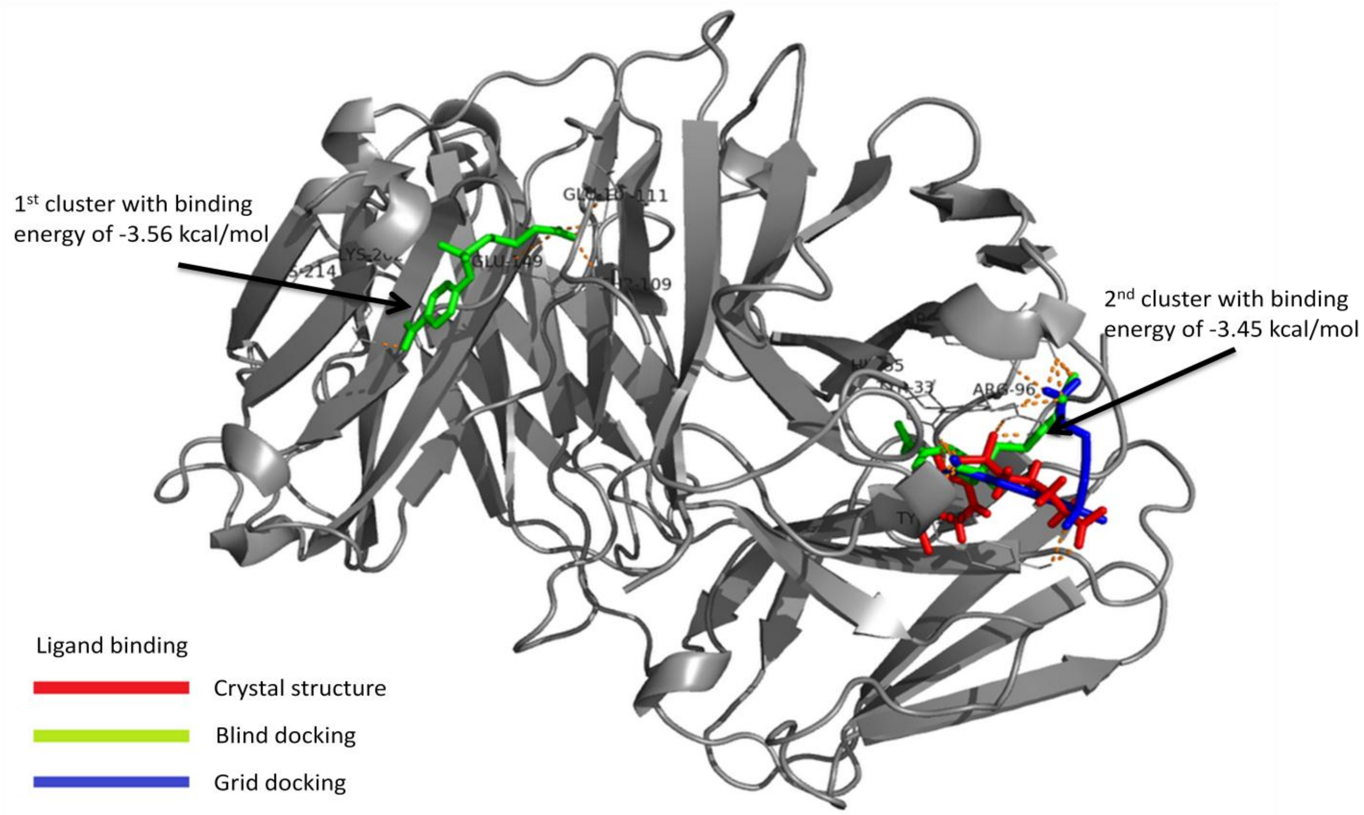

2. **Estrogen receptor – Hydroxytamoxifen (same as Figure 1 of the manuscript)**

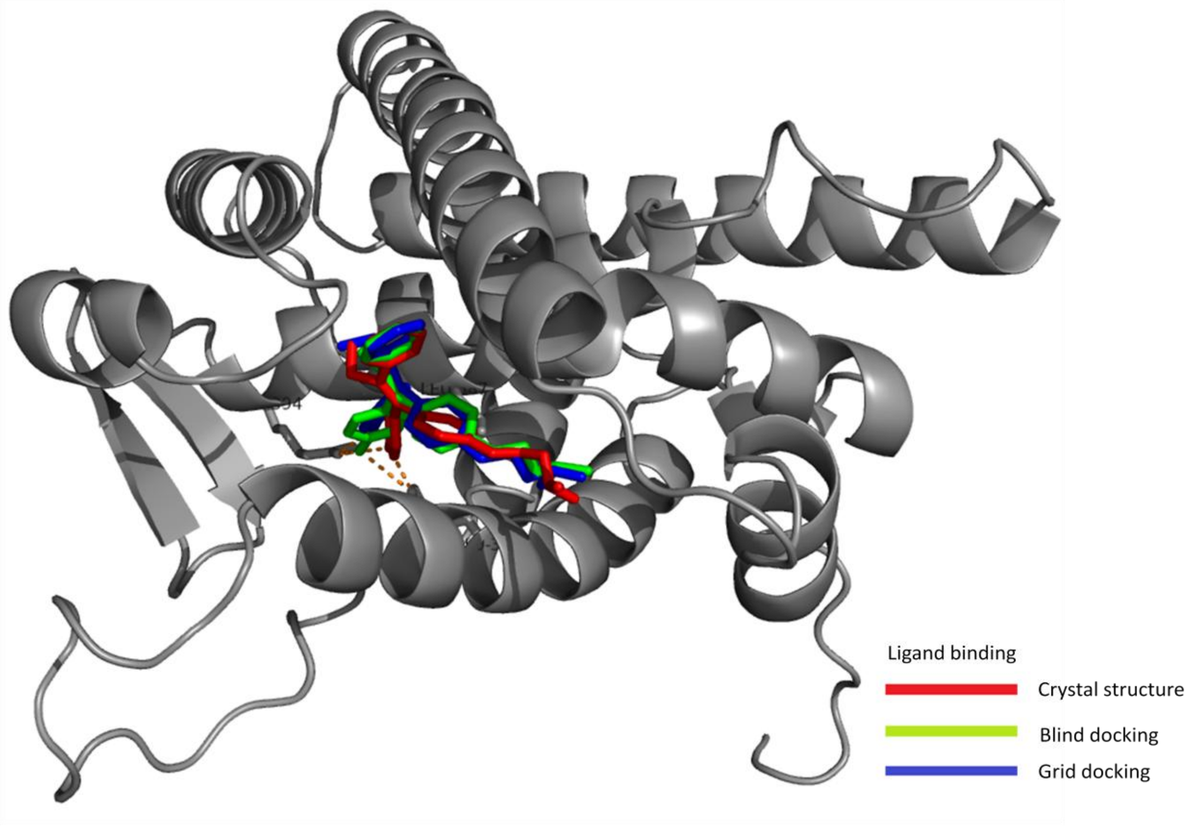

3. CBH – SNP

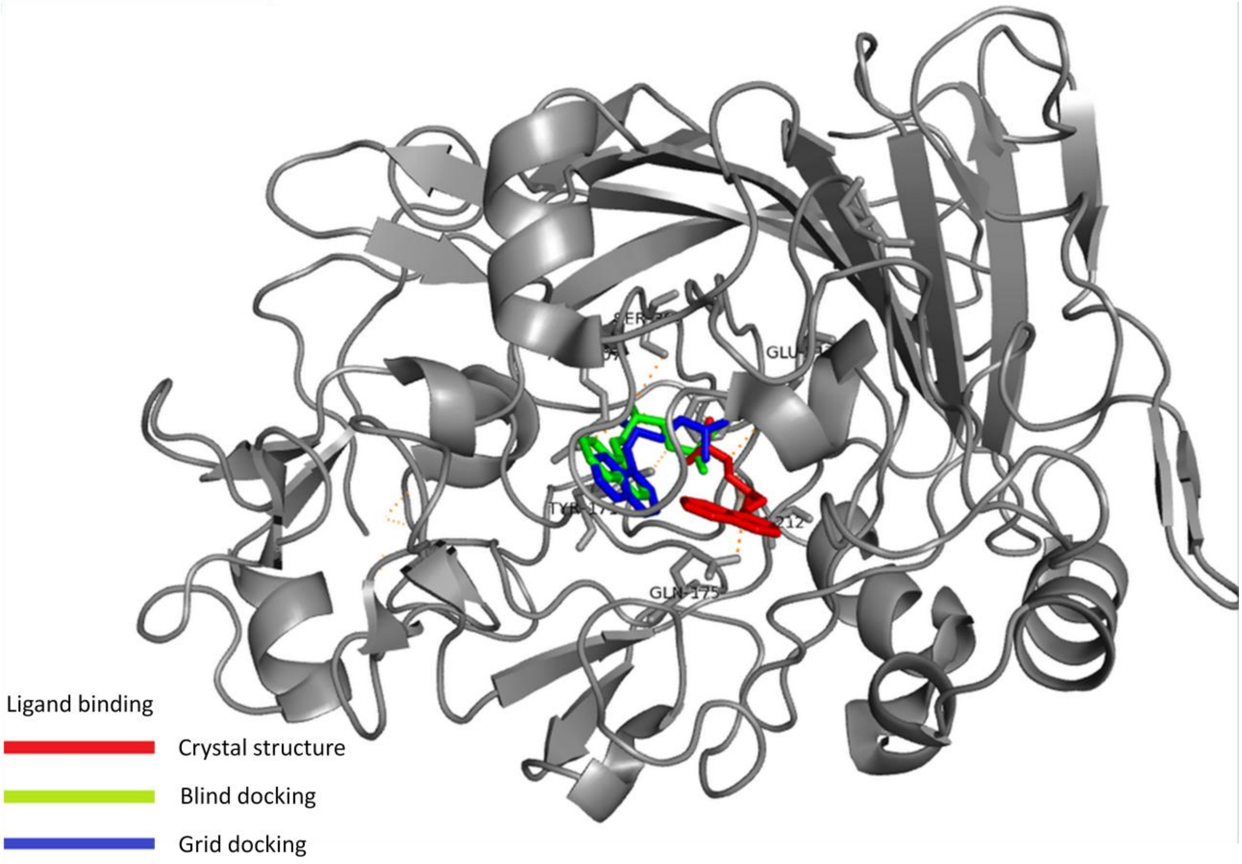
GLN-175
Ligand binding
Crystal structure
Blind docking
Grid docking

4. Avidin – Biotin

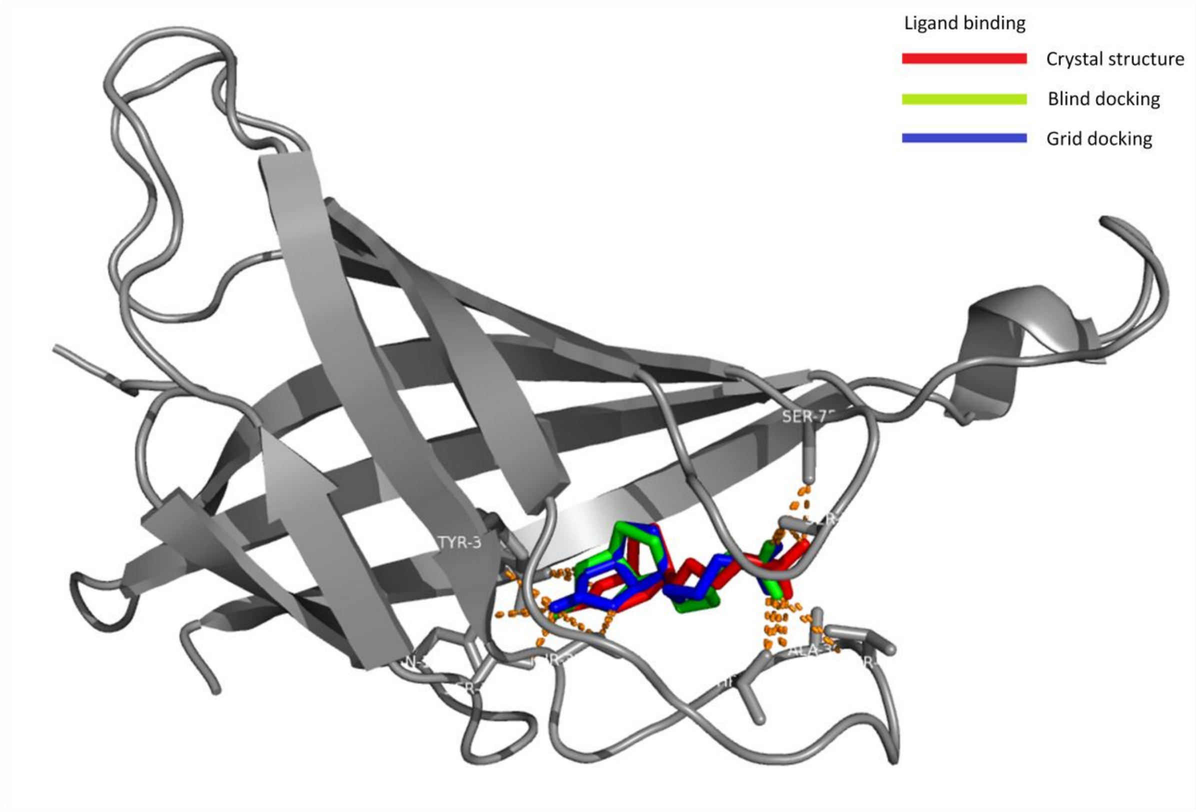

5. Glucokinase-Glucose

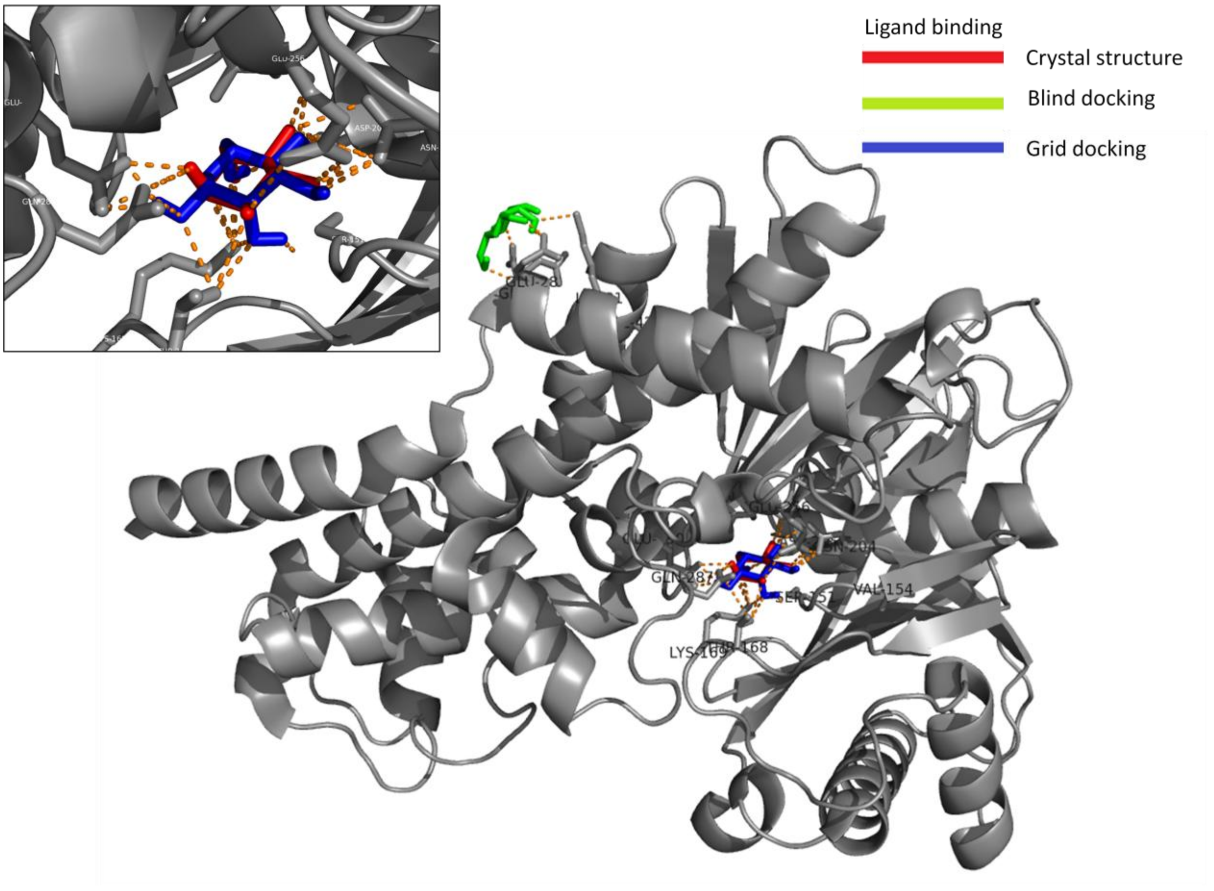

6. P450Cam-Camphor

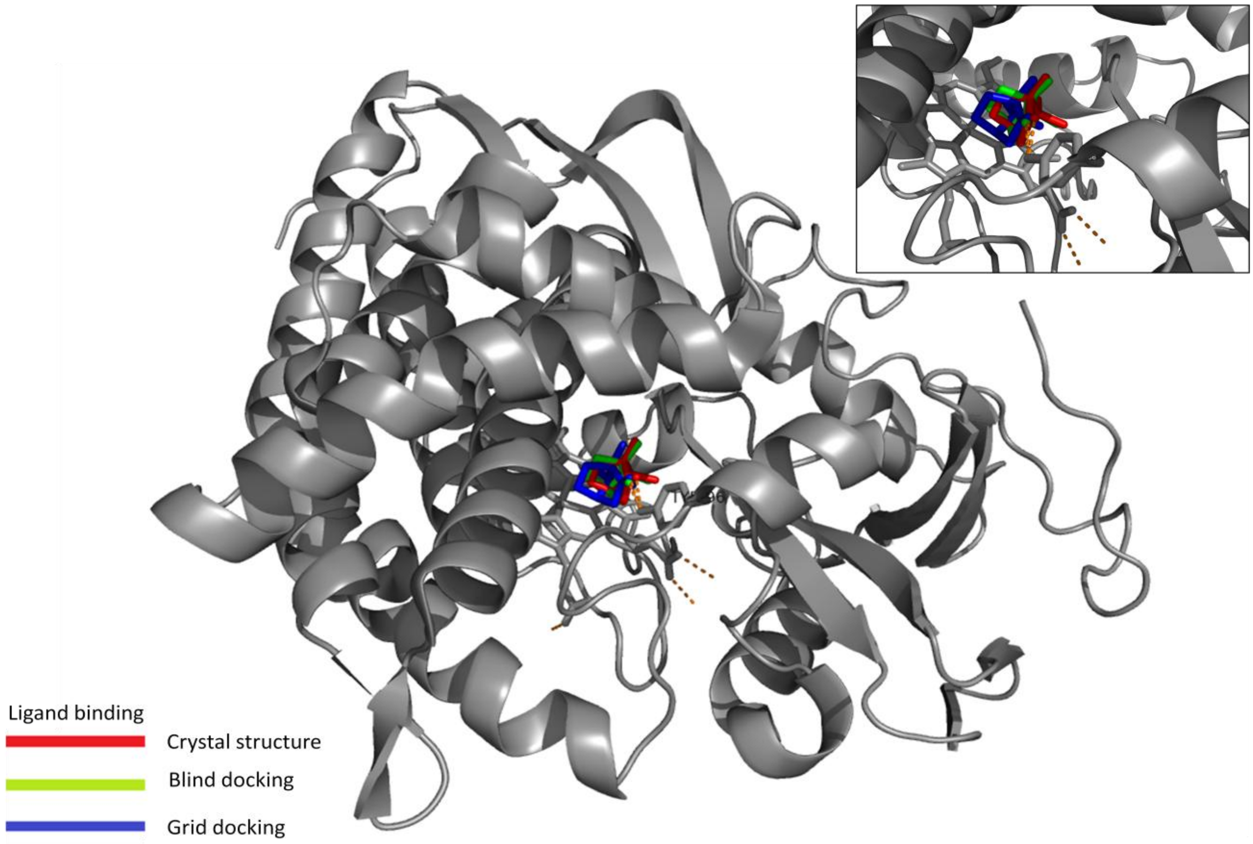

7. CYP2C9 – Flurbiprophen

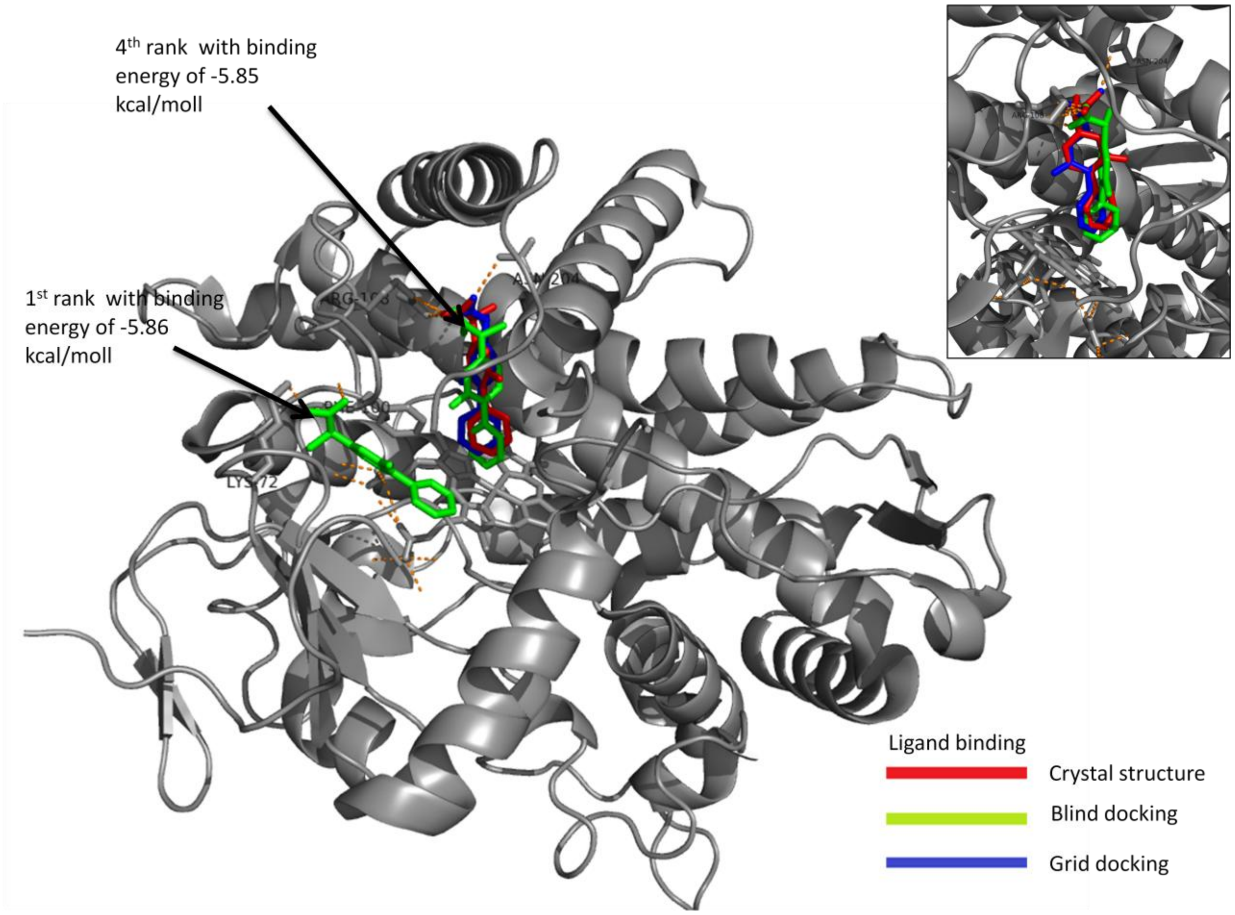

8. CYP3A4 - Erythromycin

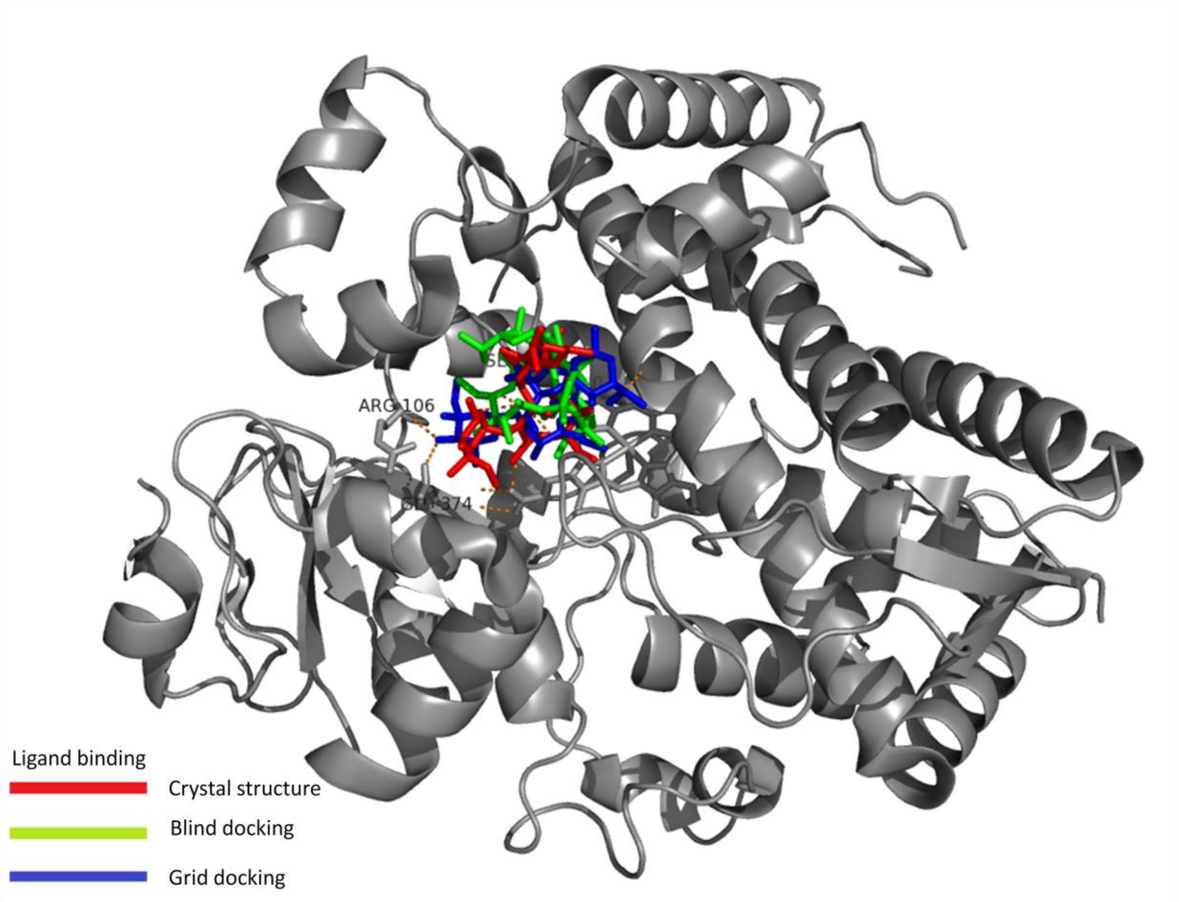

Figure A1C: Reaction schema of marker substrates for various CYPs.

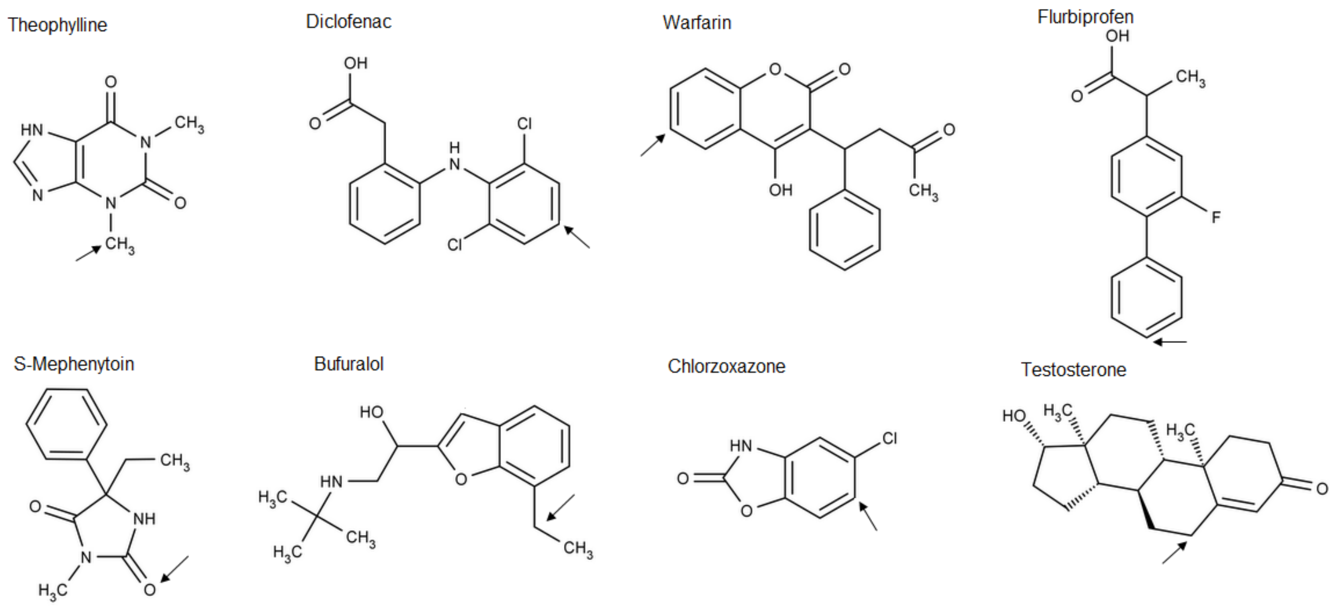

Figure A1D: Sartans

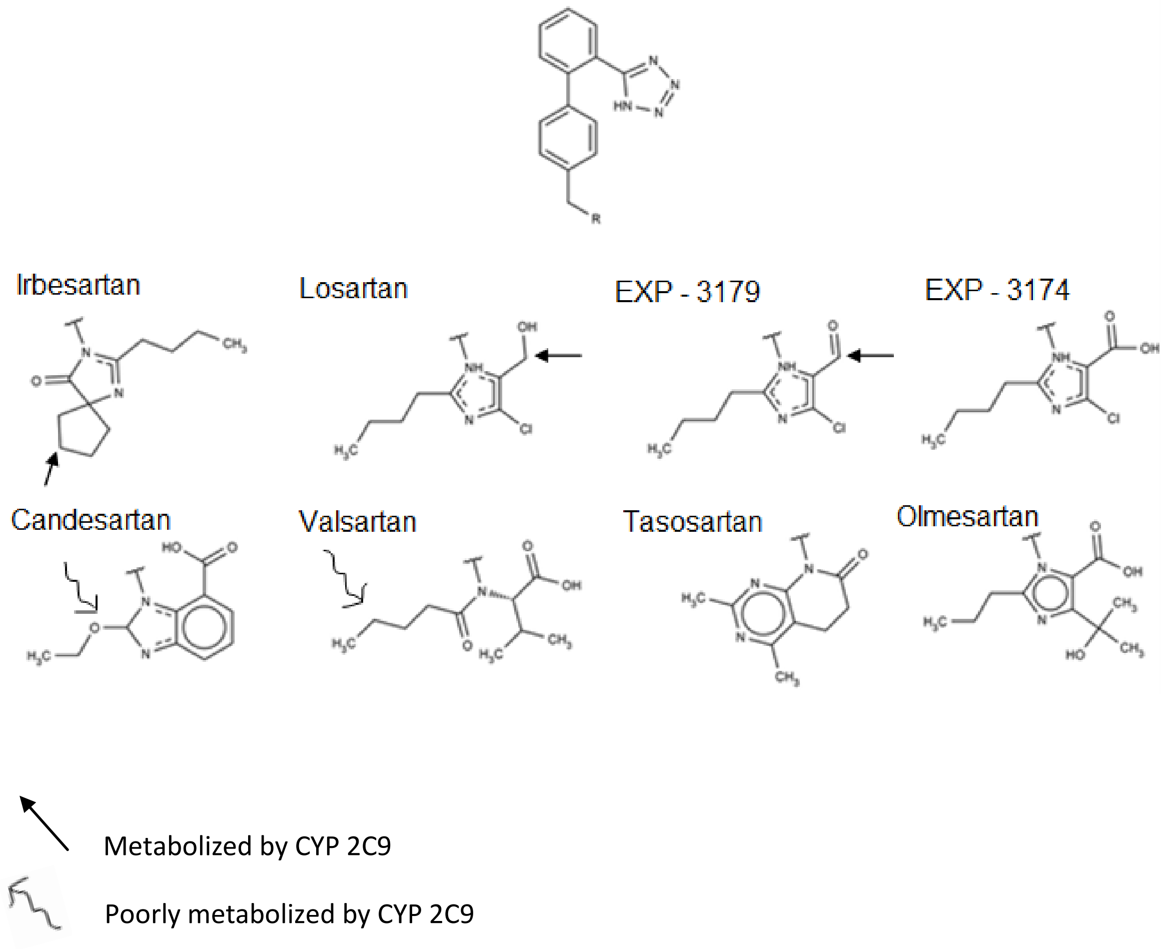

Figure A1E: Heme distal site centred binding of Sartans to 1R9O -CYP2C9

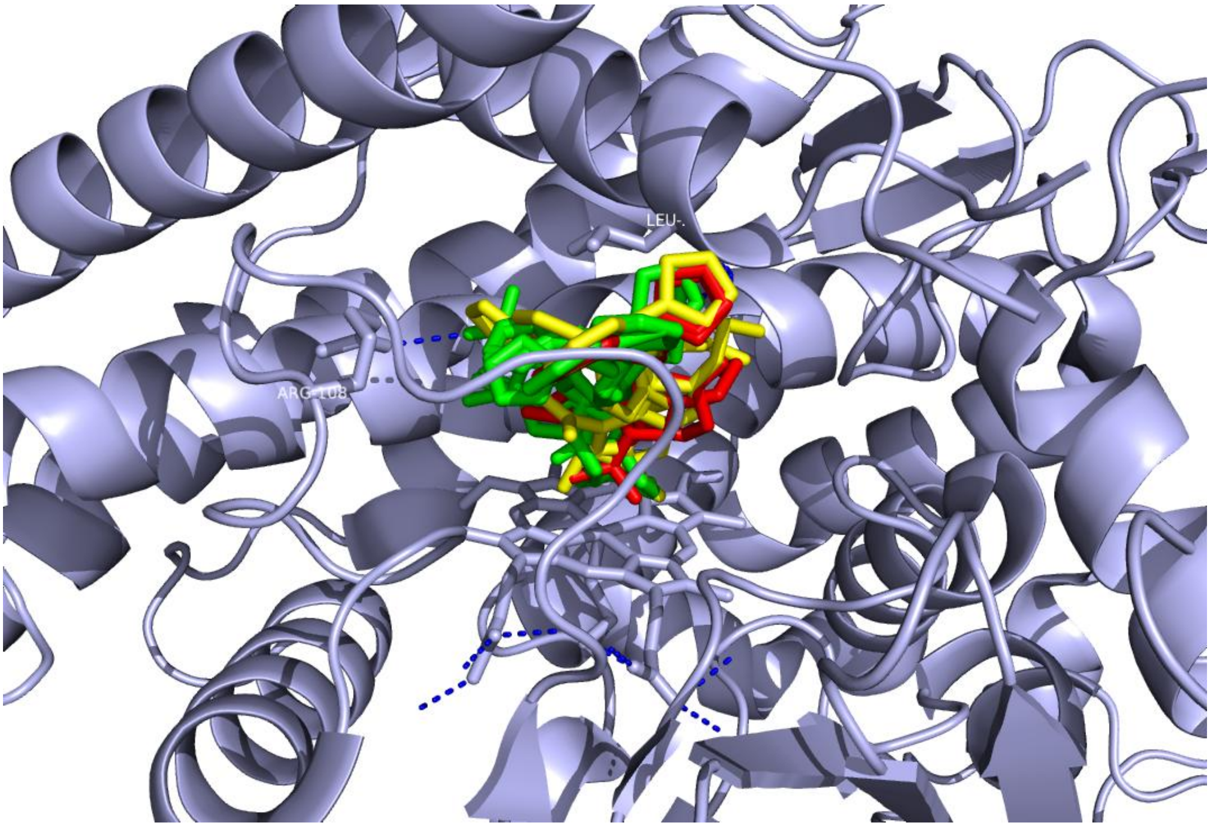

→ Substrates (green)

→ Poor Substrates (yellow)

→ Non Substrates (red)

Blind docked Sartans

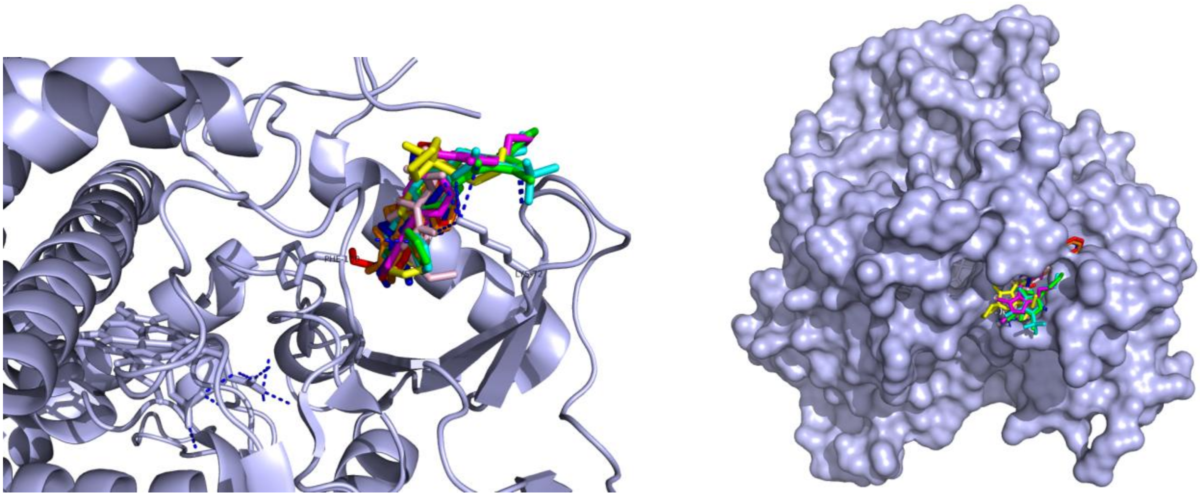

Candesartan - Red, EXP 3174 - Green, EXP 3179 - Blue, Irbesartan - Yellow, Losartan - Pink, Olmesartan - Cyan, Tasosartan - Orange, Valsartan - light pink.

Figure A1F: Statins

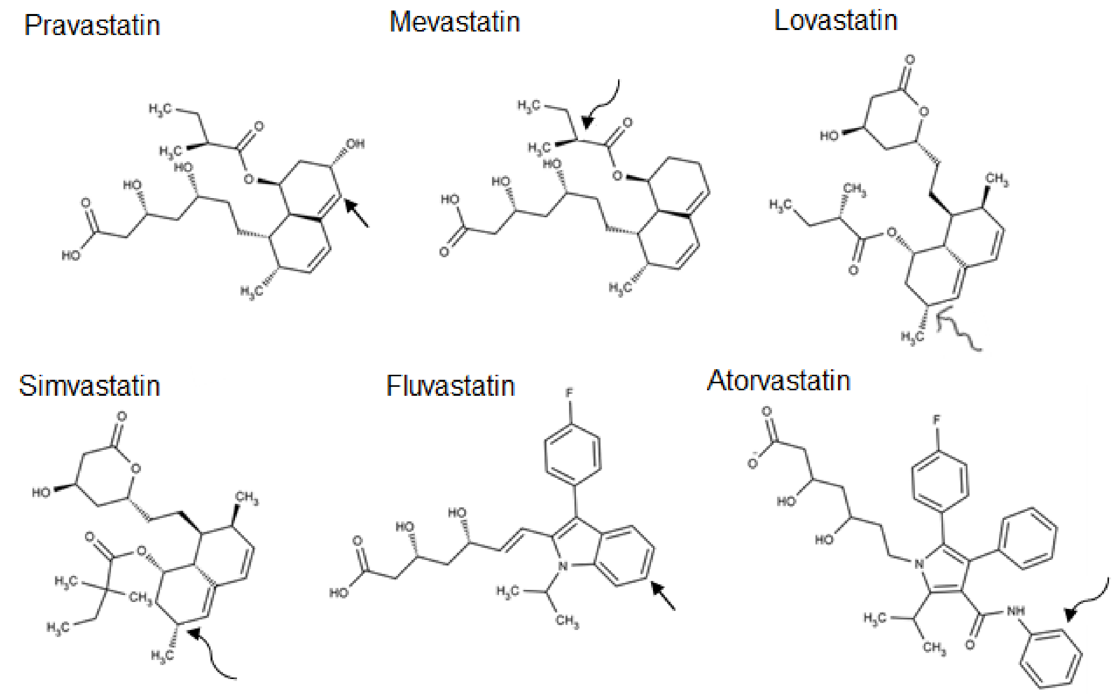

Metabolized by CYP 2C9

Poorly metabolized by CYP 2C9

Metabolized by CYP 3A4

Figure A1G: Binding of Statins to CYP2C9 (1R9O)

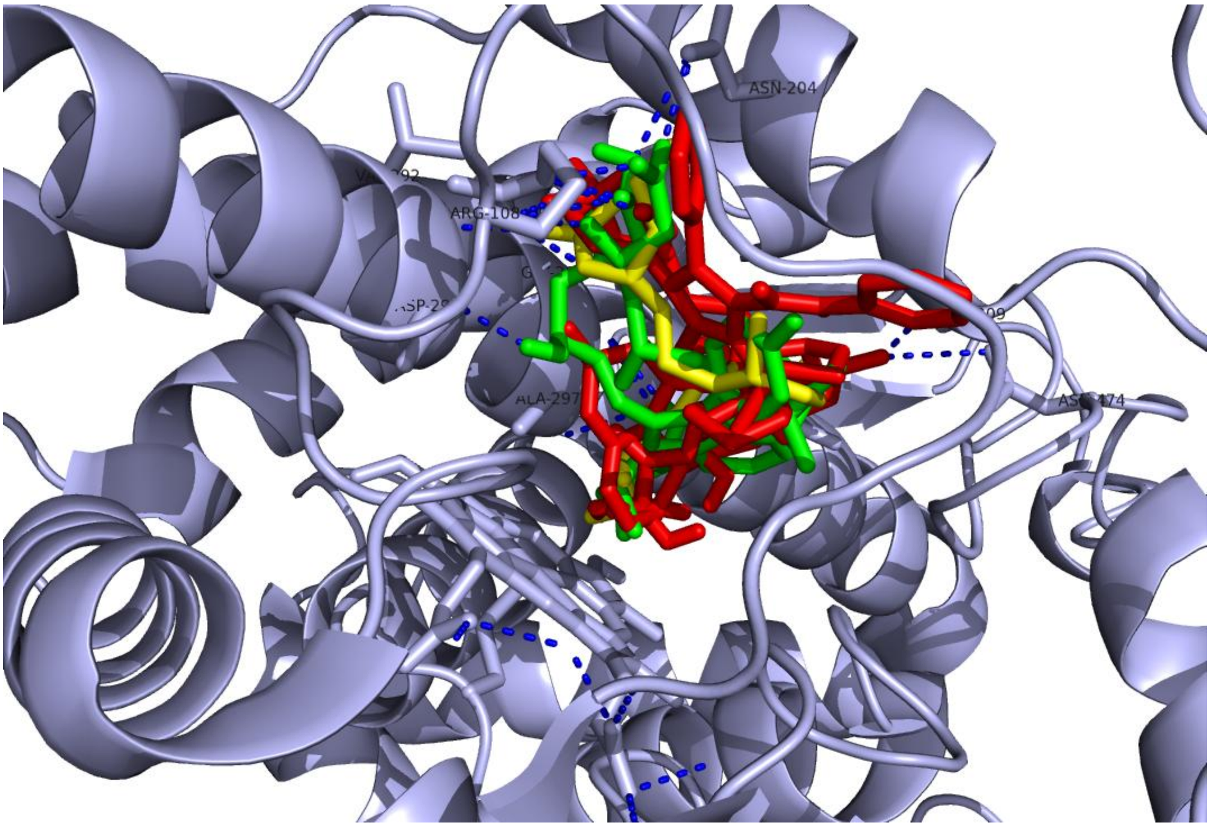

Green → Substrates

Yellow → Poor Substrates

Red → Non Substrates

Figure A1H: Blind docking results of Statins to CYP2C9 (1R9O)

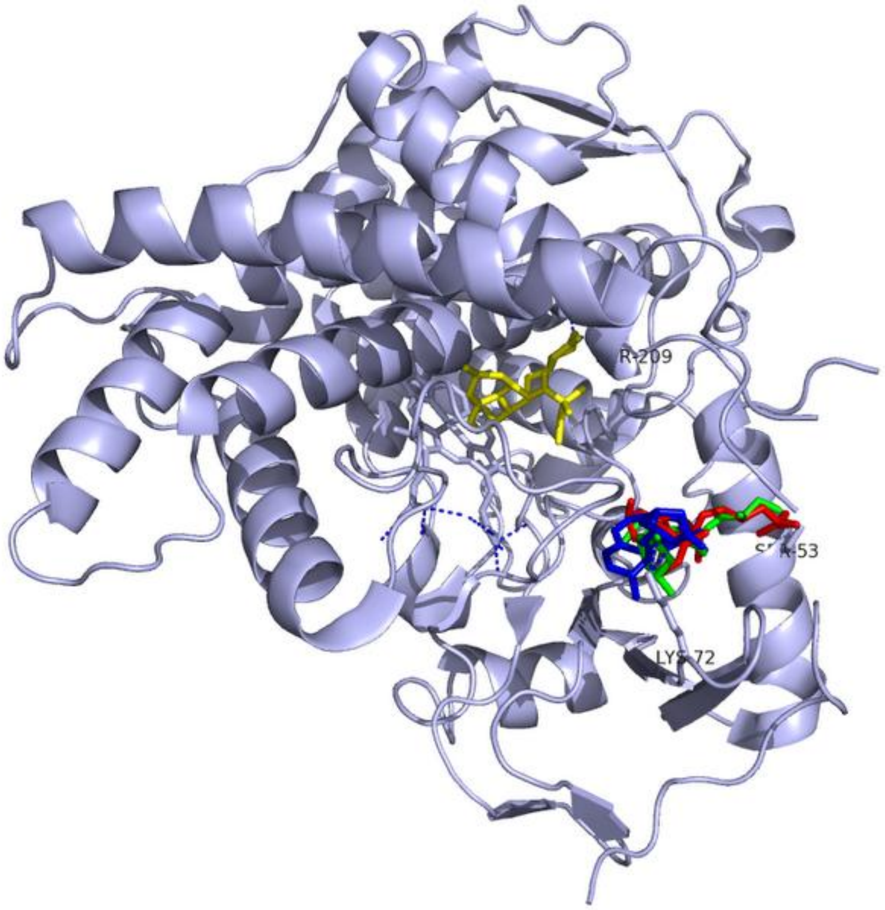

Pravastatin – red, Lovastatin – green, Mevastatin – blue, Simvastatin – Yellow

Figure A1I: Triptans

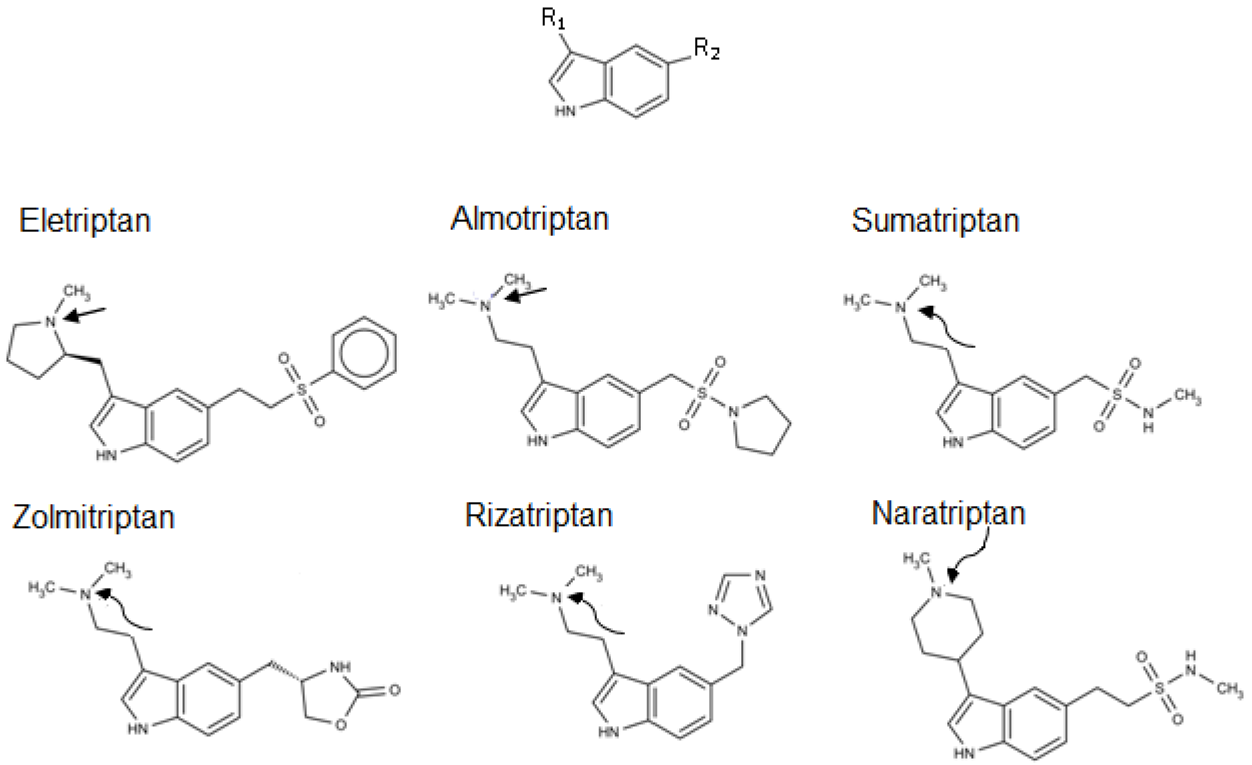

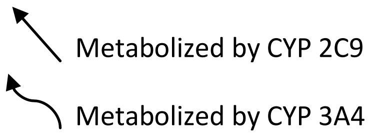

Figure A1J: Binding of Omeprazole to CYP2C9 and CYP2C19

1. CYP2C9-Omeprazole

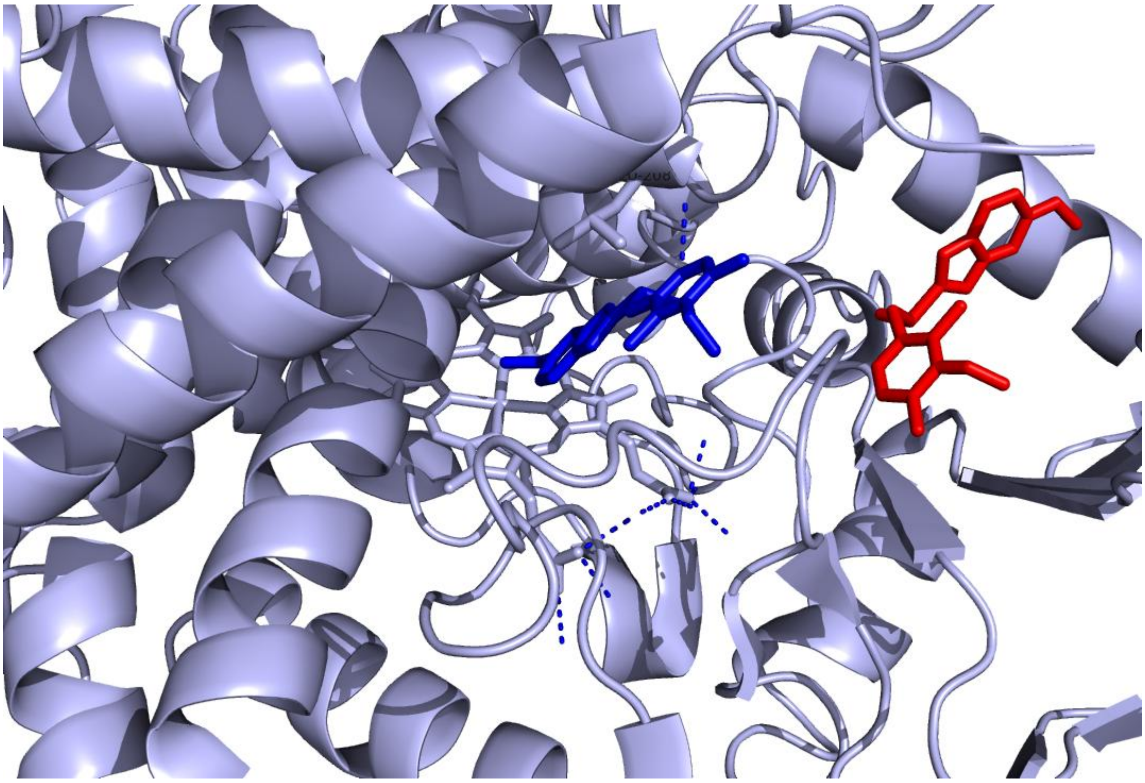

2. CYP2C19-Omeprazole

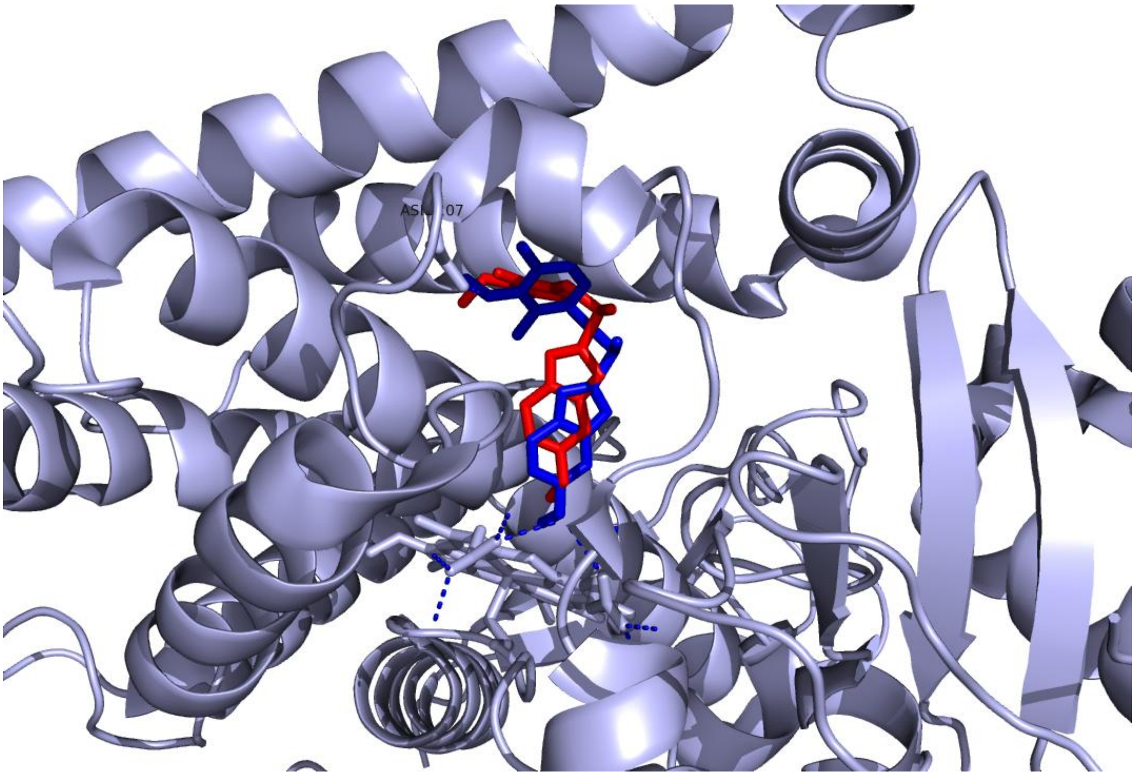

Heme distal site centred docking   Blind docking

Figure A1K: Binding of oxyresorufins to 1A2 and 3A4

1. CYP1A2 – Blind docking

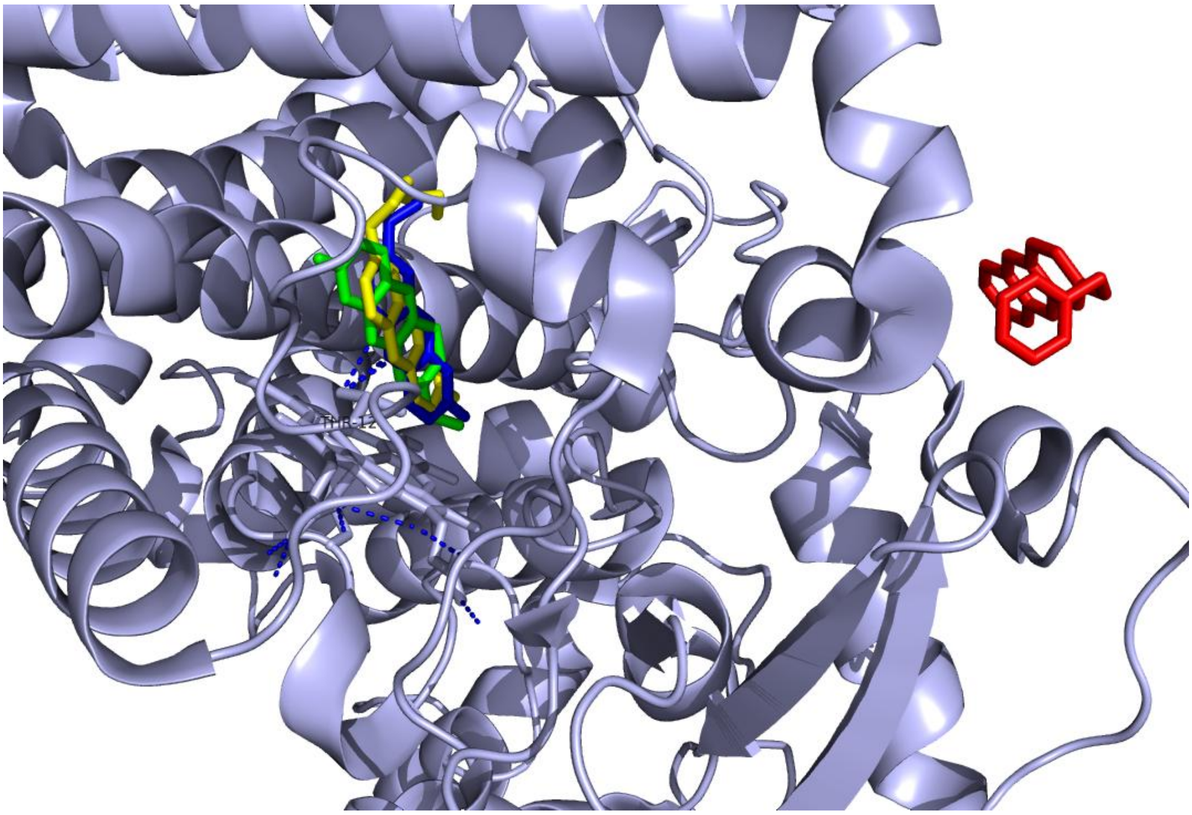

2. CYP1A2 - Heme distal site centred docking

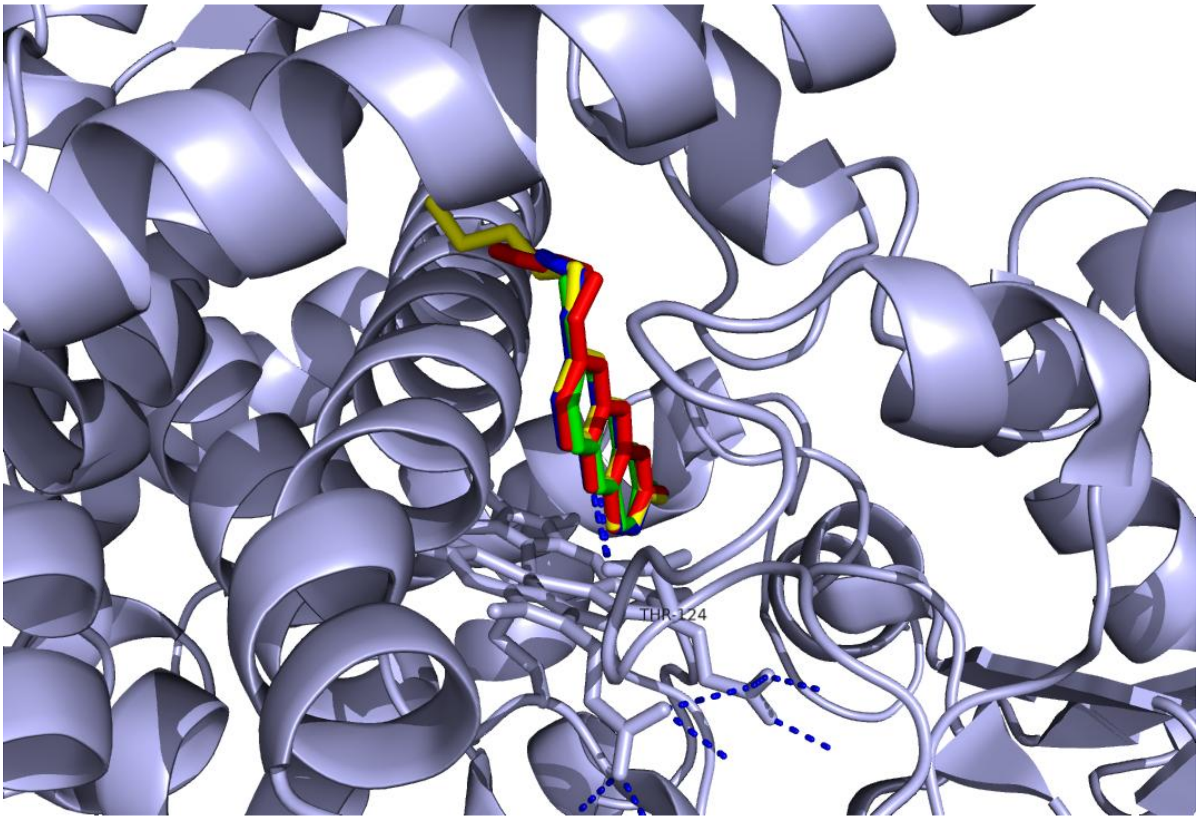

BOR MOR EOR POR

3. CYP3A4 – Blind docking

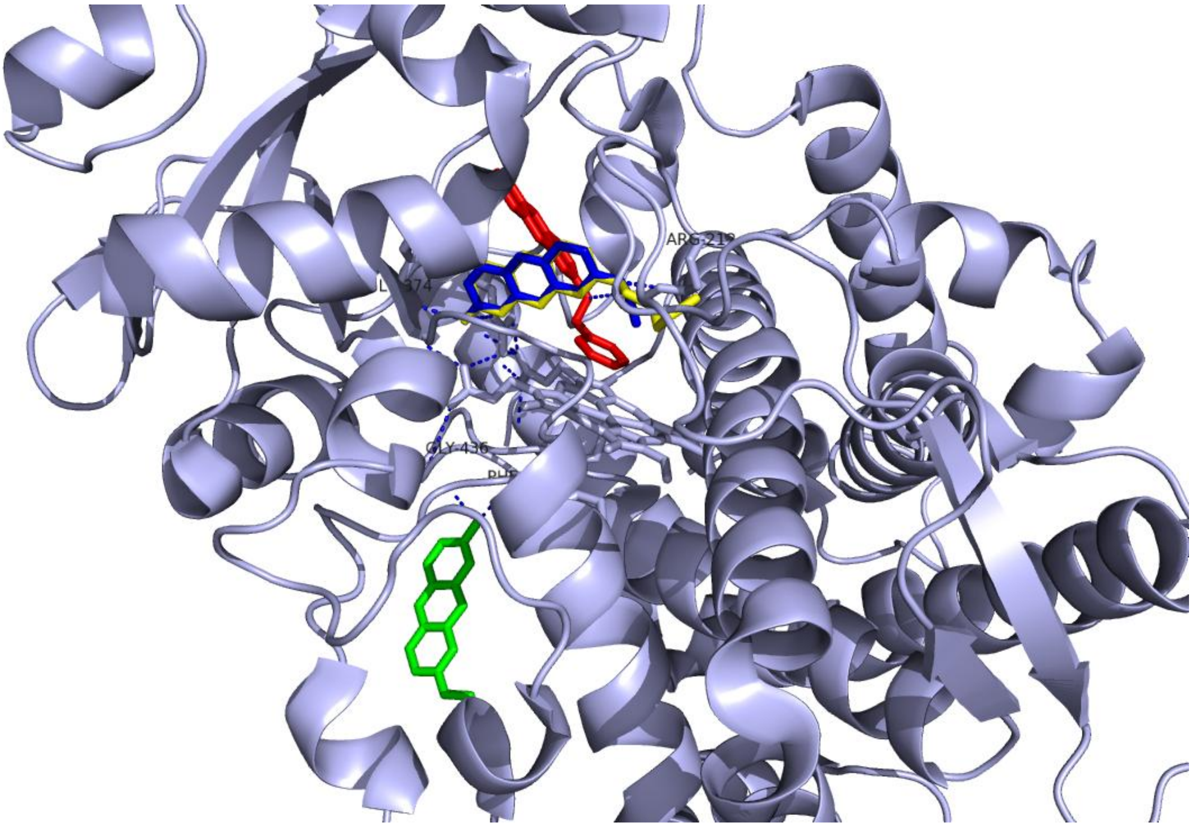

4. CYP3A4 - Heme distal site centred docking

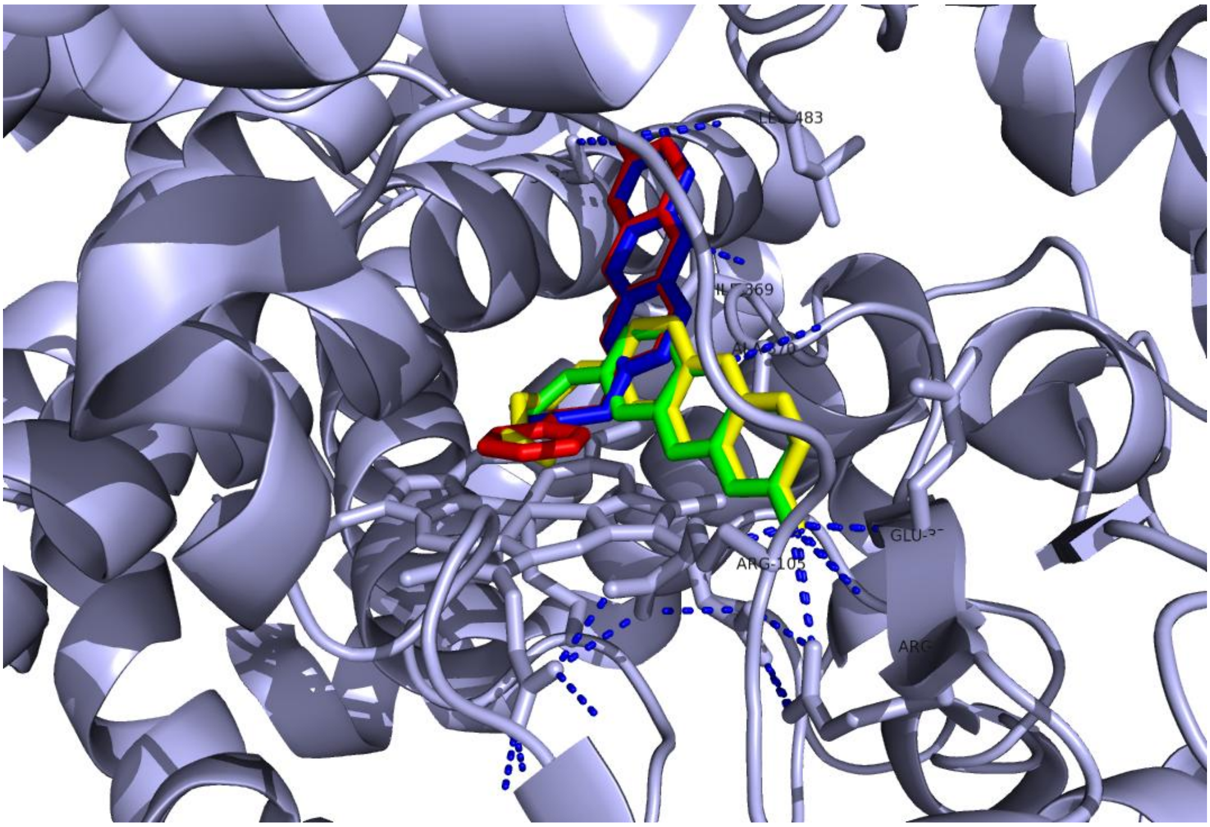

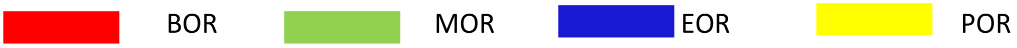

Figure A1L: Binding of coumarin and testosterone with CYPs- 2A6 and 3A4.

1. CYP2A6 wild type with Coumarin and Testosterone

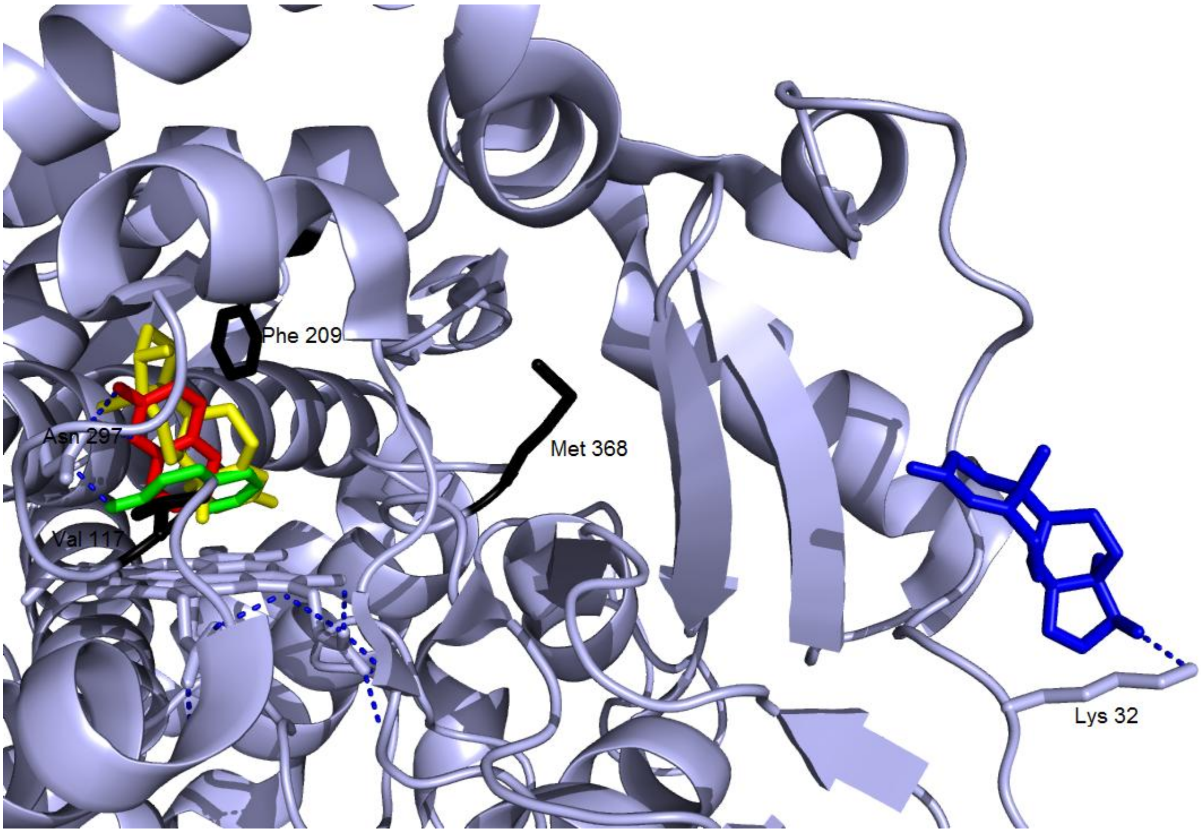

2. CYP2A6 mutant with Coumarin and Testosterone

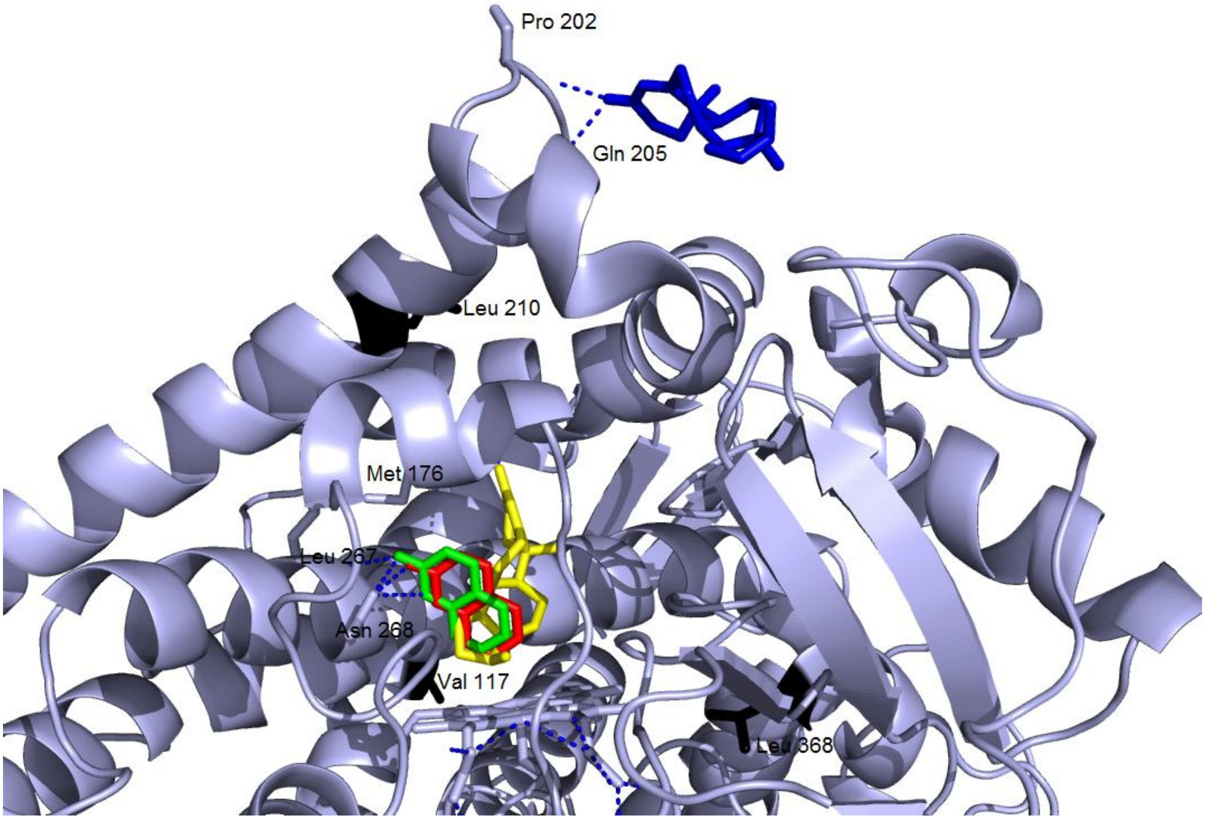

Cou Blind

Cou Centred

Test Blind

Test Centred

3. CYP3A4 wild type with Coumarin and Testosterone

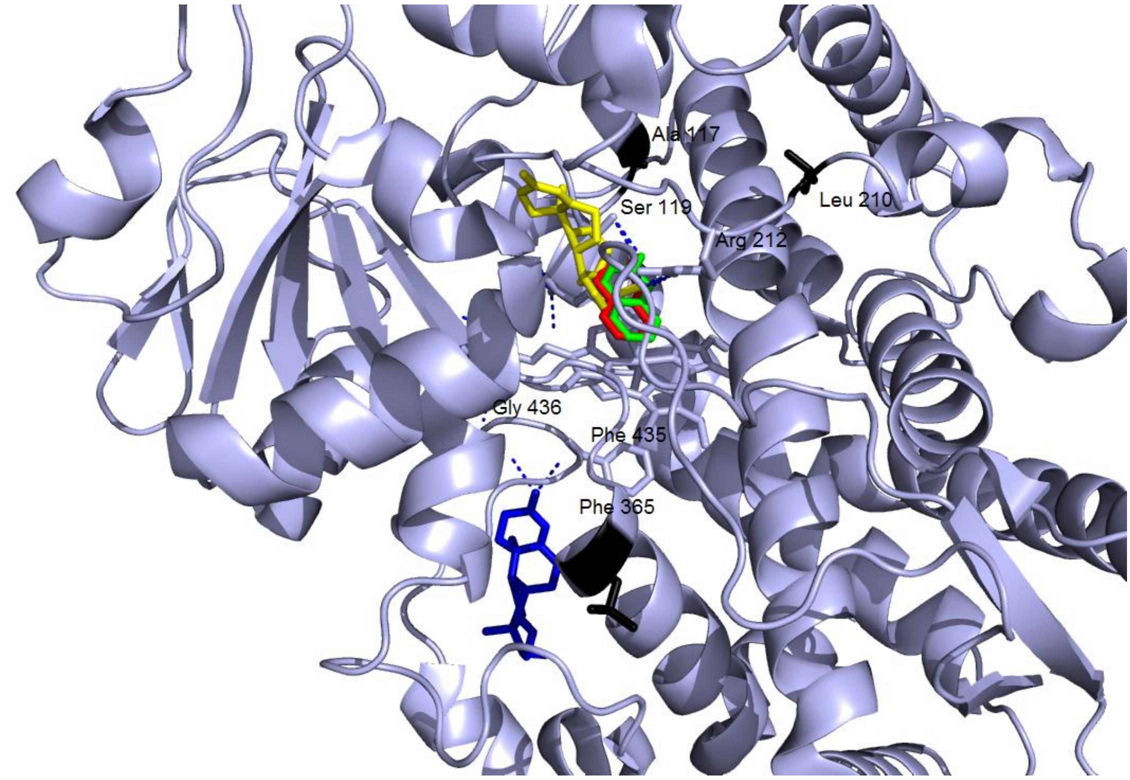

4. CYP3A4 mutant with Coumarin and Testosterone

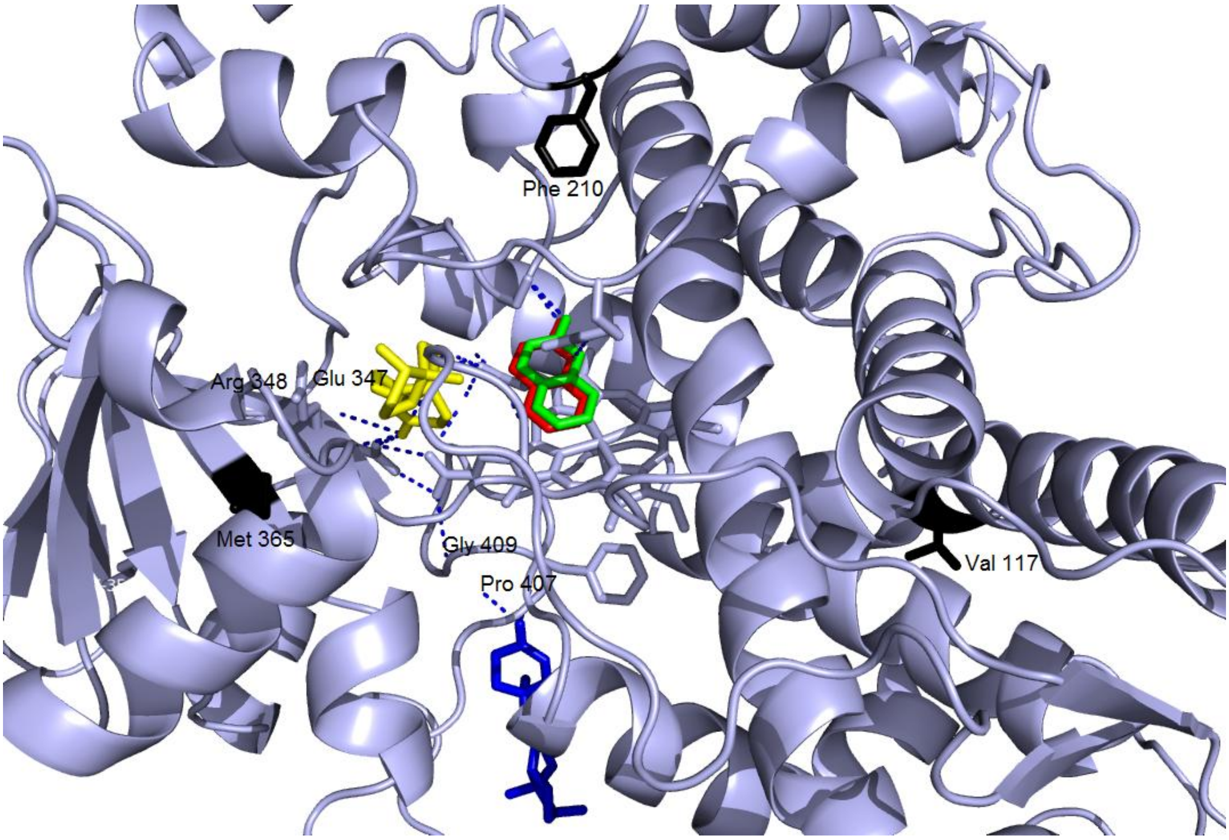

Cou Blind   Cou Centred   Test Blind   Test Centred

5. CYP2A6- Key mutated amino acids

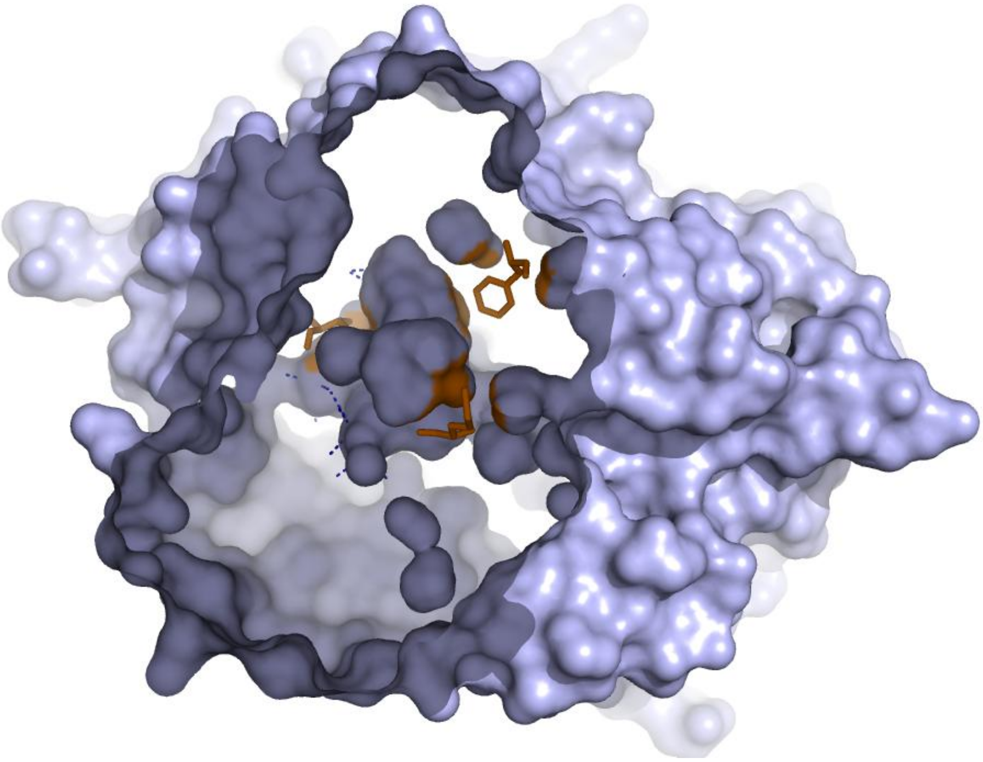

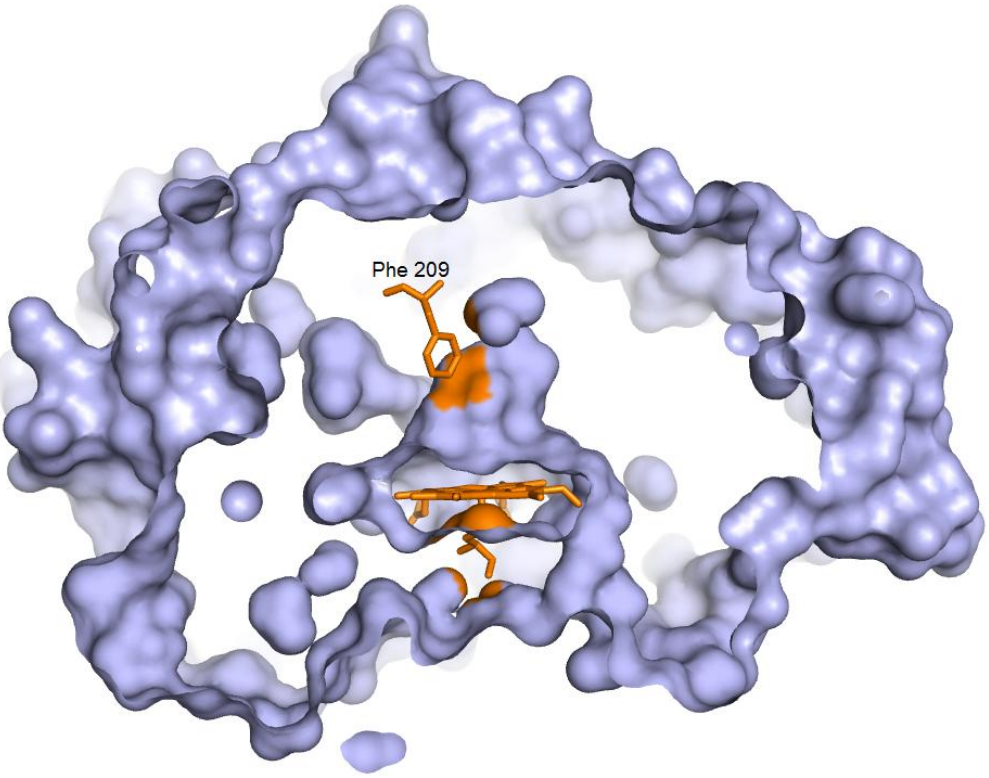

6. 3A4 key mutated amino acids

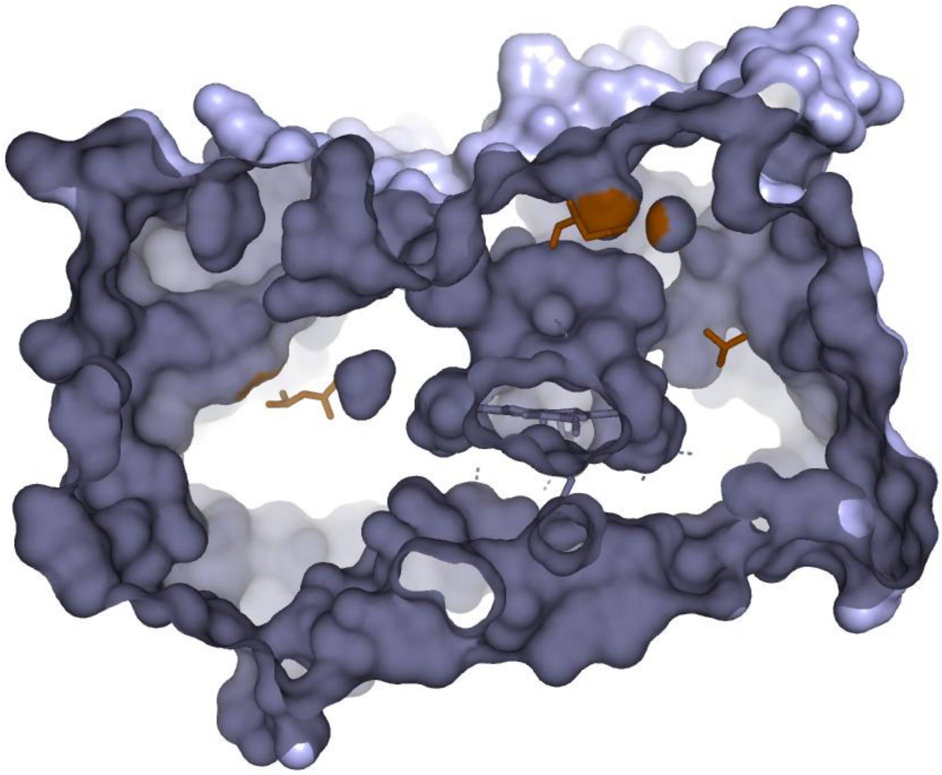

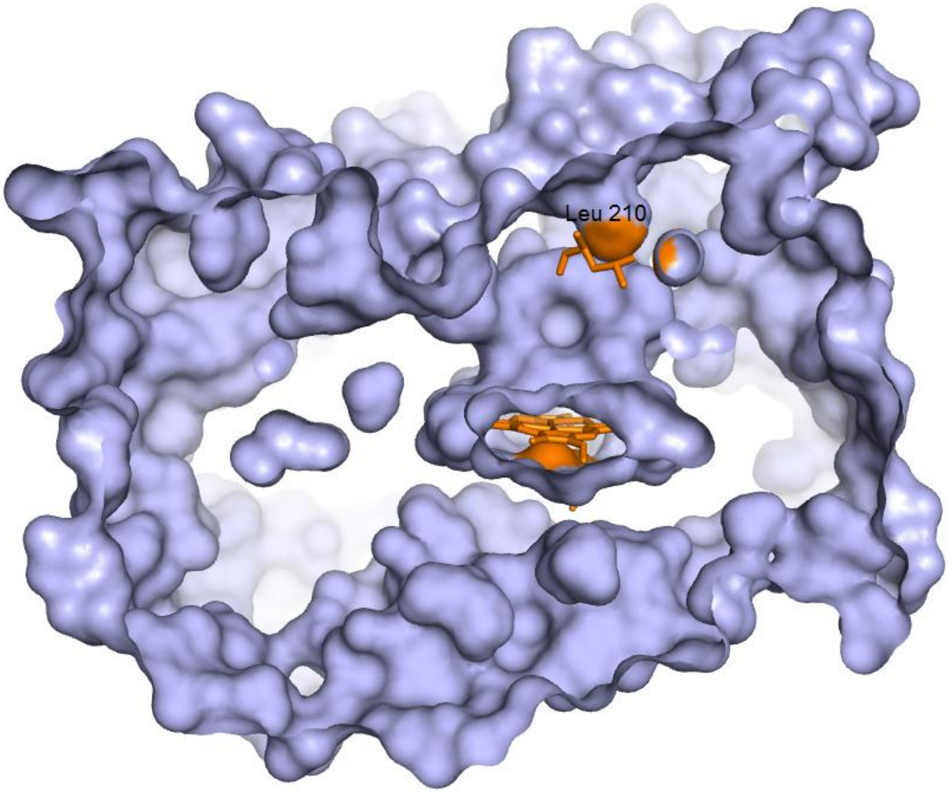

Figure A1M: Structures of molecules and schema of reactions probed via *in silico* approaches for understanding drug interactions

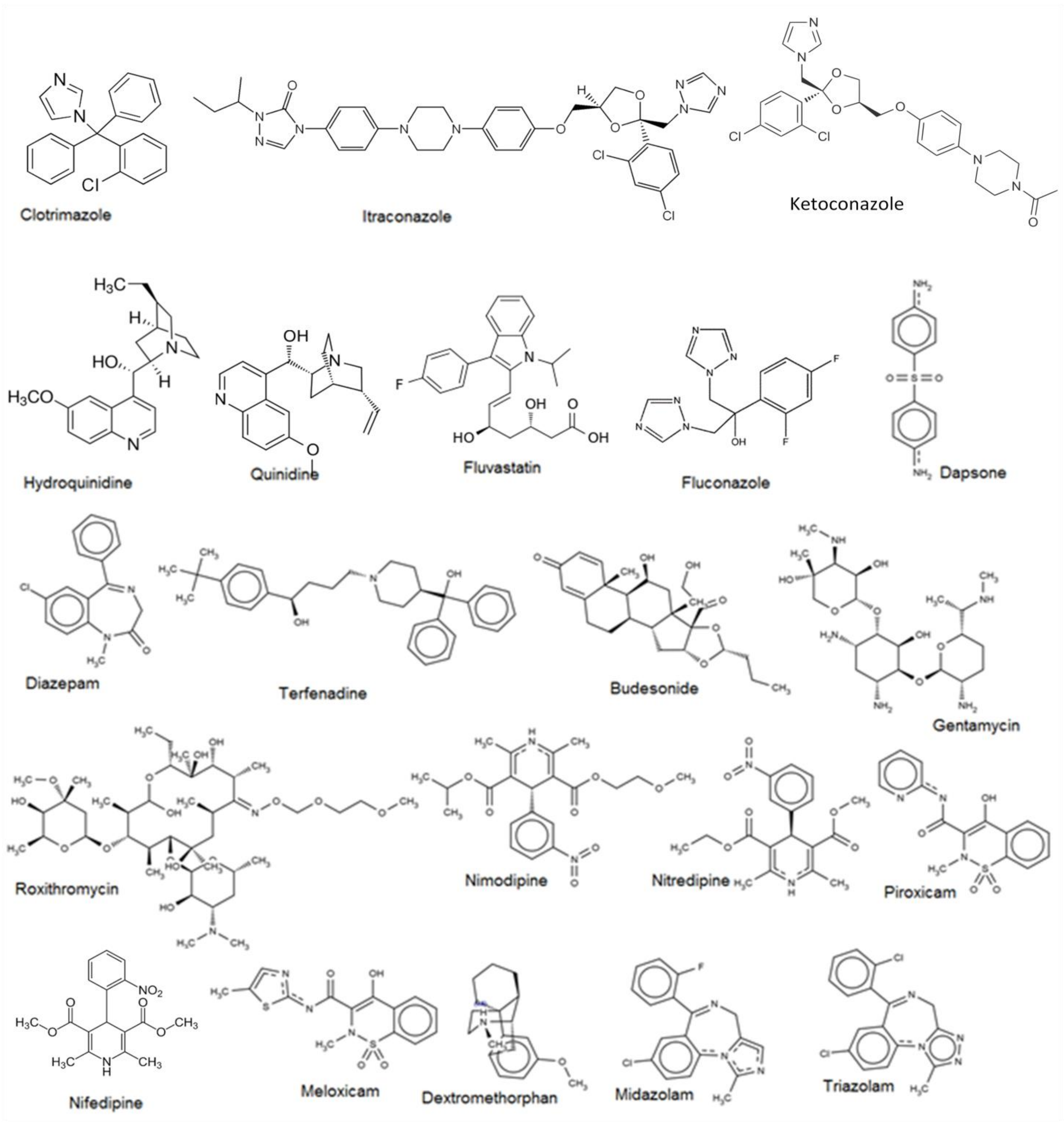

Figure A1N: Conserved binding of some modulators and substrates outside heme distal pocket in CYPs.

1. Conserved binding locus in substrates' blind dockings with CYP2C9

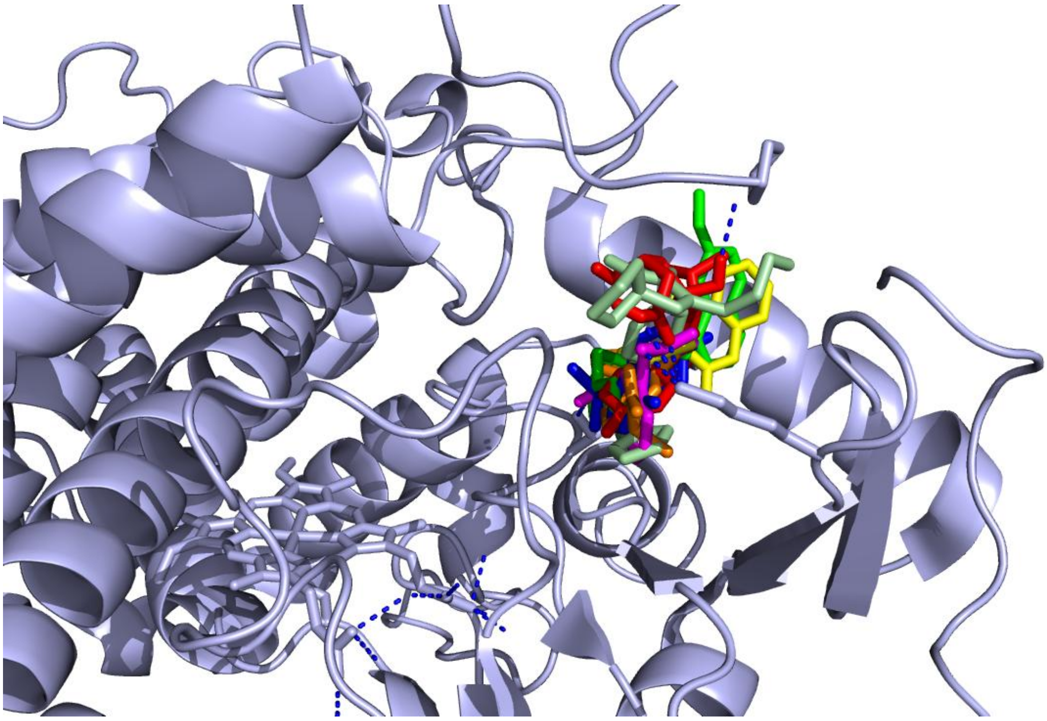

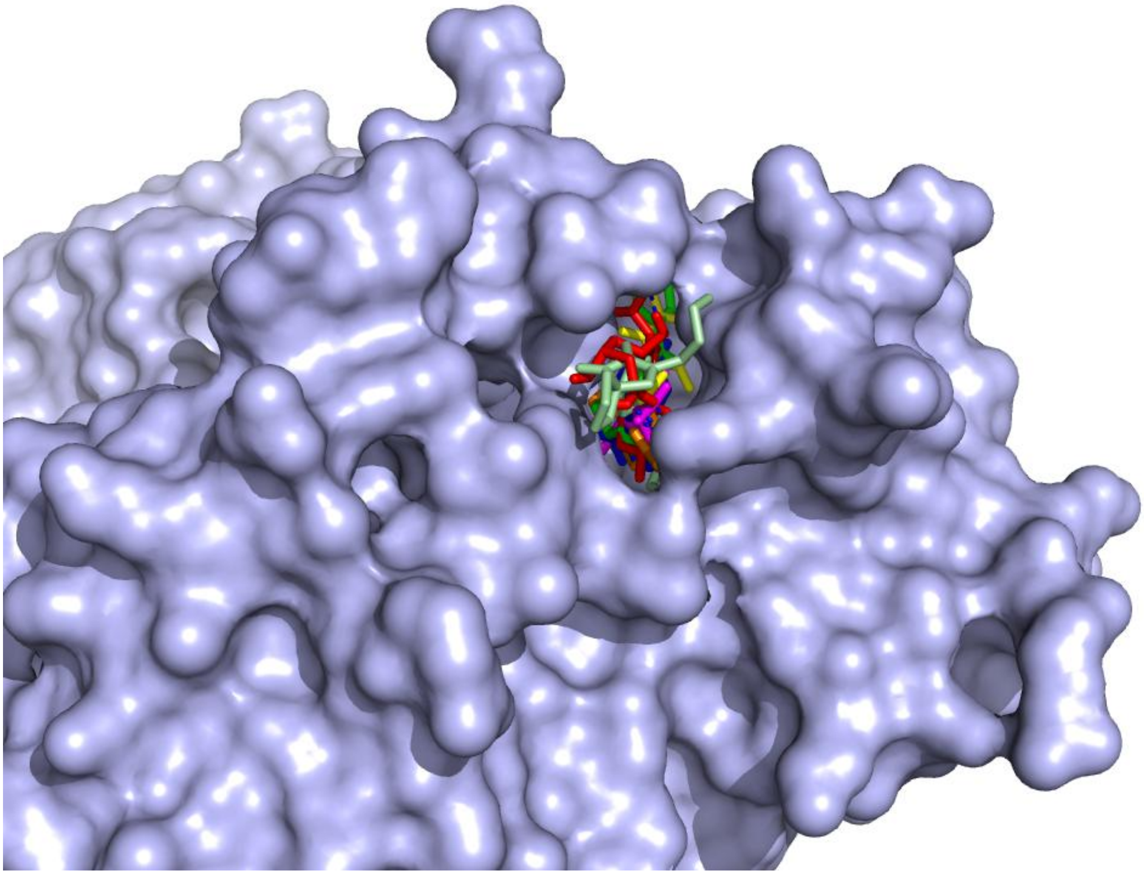

Diclofenac – Pink, Flurbiprofen – Orange, Quinidine – Green, Dapsone – Blue, Fluvastatin – Red, Irbesartan – Light green, Warfarin – Yellow

2. Conserved binding loci in substrates' blind dockings of CYP3A4

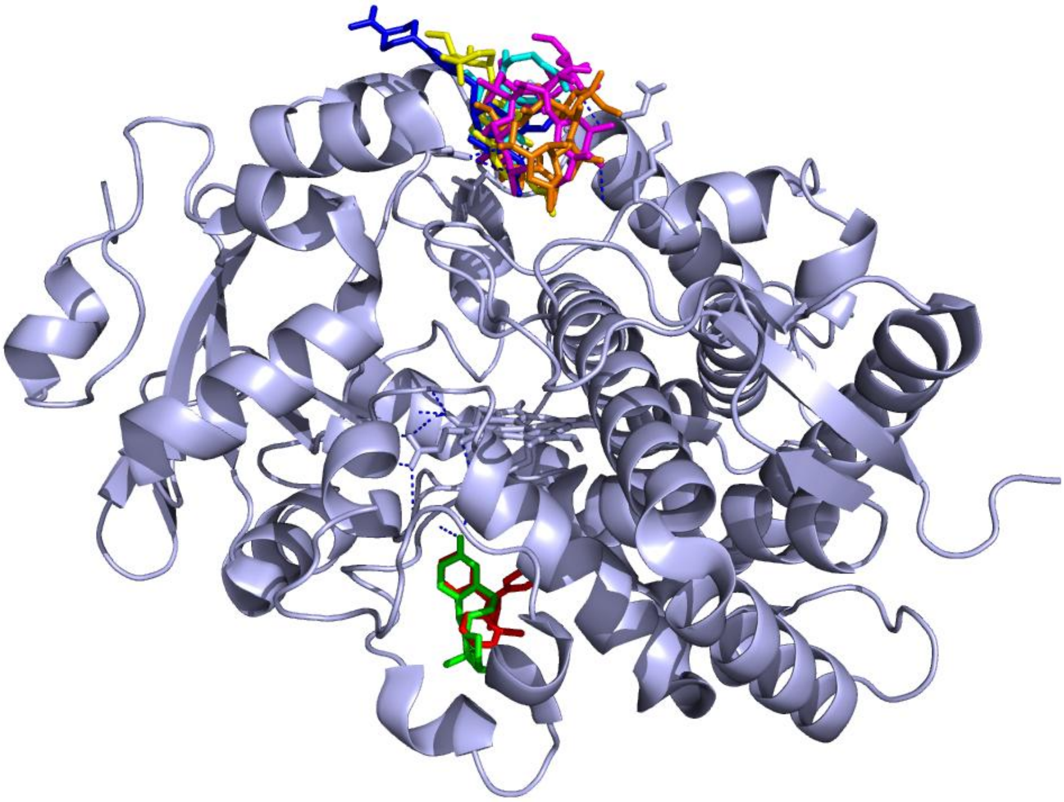

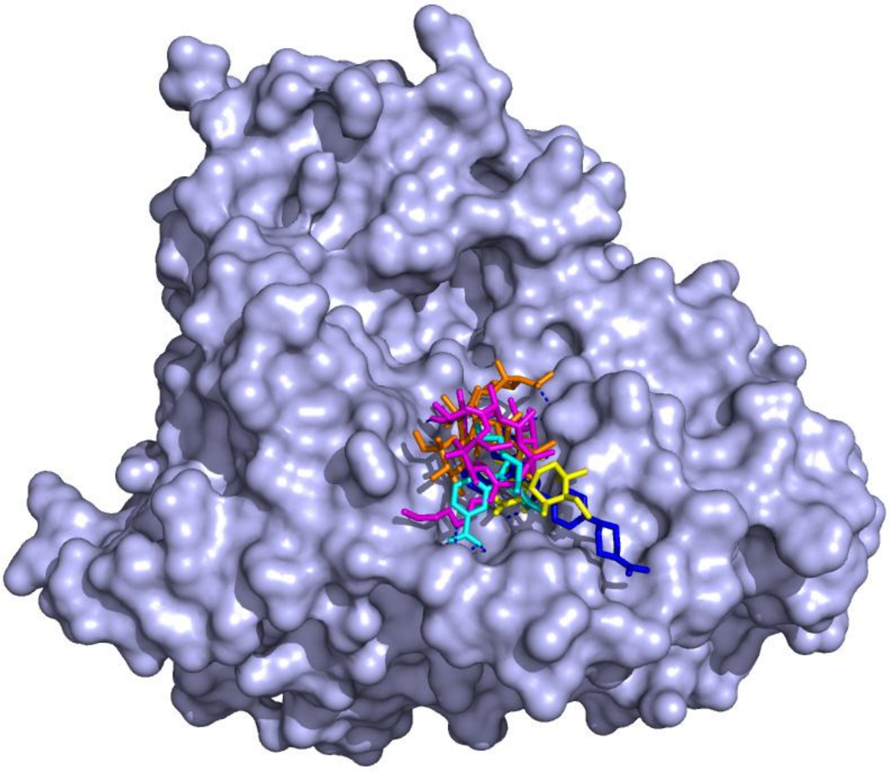

Clotrimazole – Red, Testosterone – Green, Ketoconozole – Blue, Gentamicin – Yellow, Roxithromycin – Pink, Nimodepine – Cyan, Erythromycin - Orange

Figure A1O: Binding of Azoles to CYPs (particularly 3A4)

1. The blind and centred docking of clotrimazole to CYP3A4

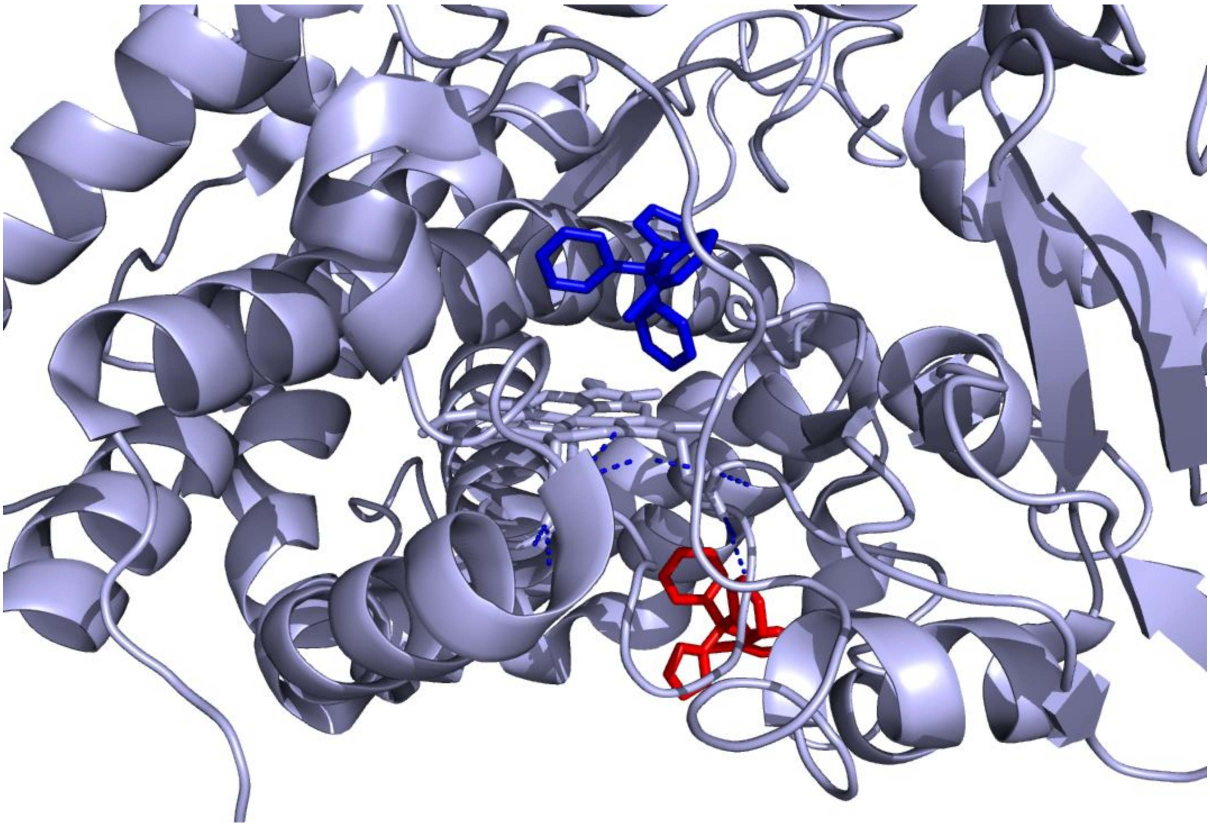

Heme distal site centred docking | Blind docking

2a. The distal cavity of CYP3A4 and clotrimazole

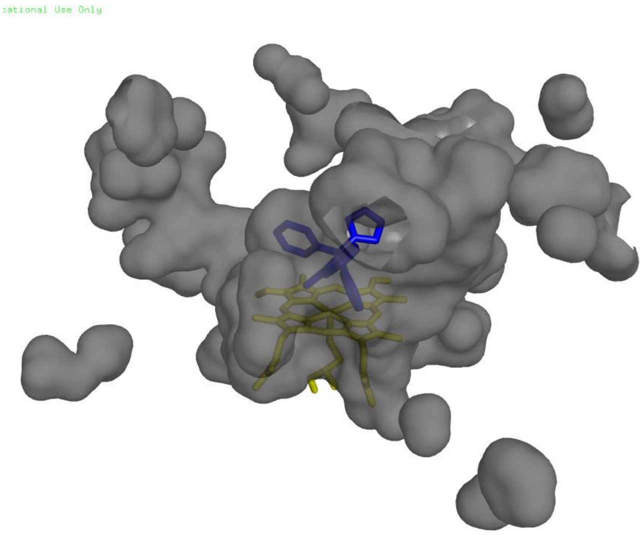

2b. Surface view showing blind-docked clotrimazole

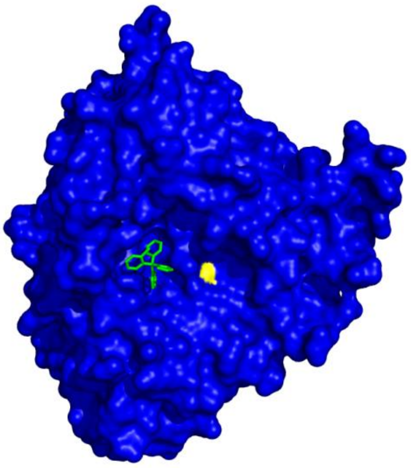

The distal cavity is visualized, along with the major aperture. The dimensions of the centre-docked clotrimazole molecule can be compared with respect to the entrance of the channel. The image on the right shows CYP3A4 seen from the proximal side. (The axial ligand cysteine residue is shown in yellow.)

3. Blind docking of various large "azoles" to CYP3A4

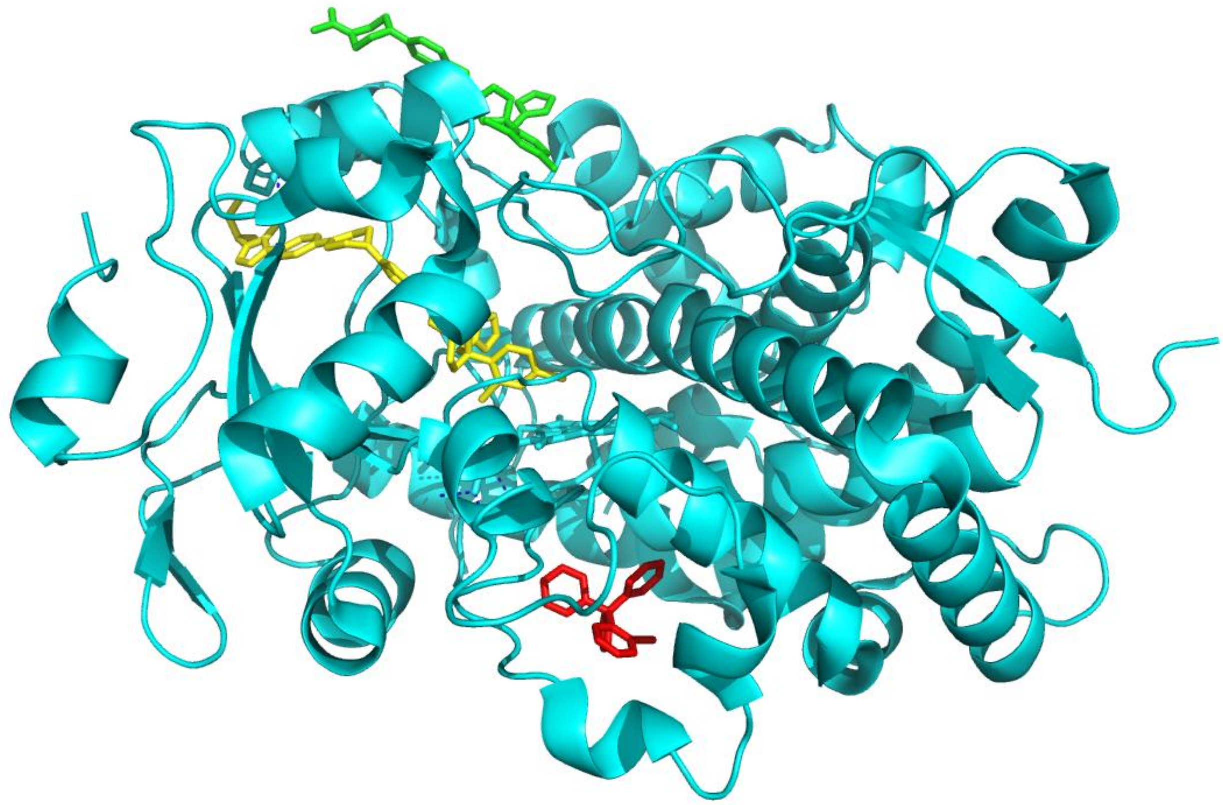

Clotrimazole – Red, Ketoconazole – Green, Itraconazole – Yellow

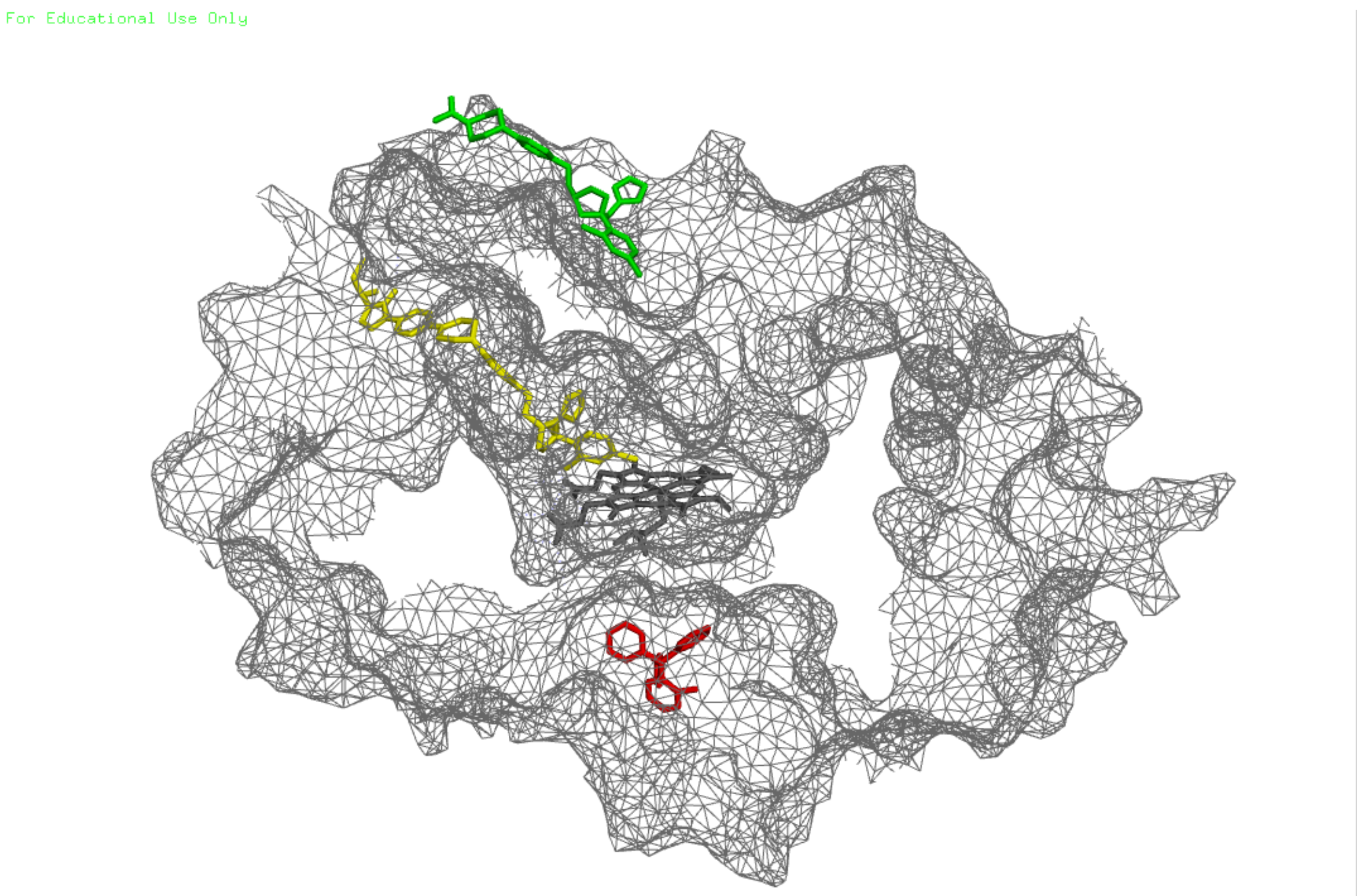

4. Blind docking of various large "azoles" to CYP1A2

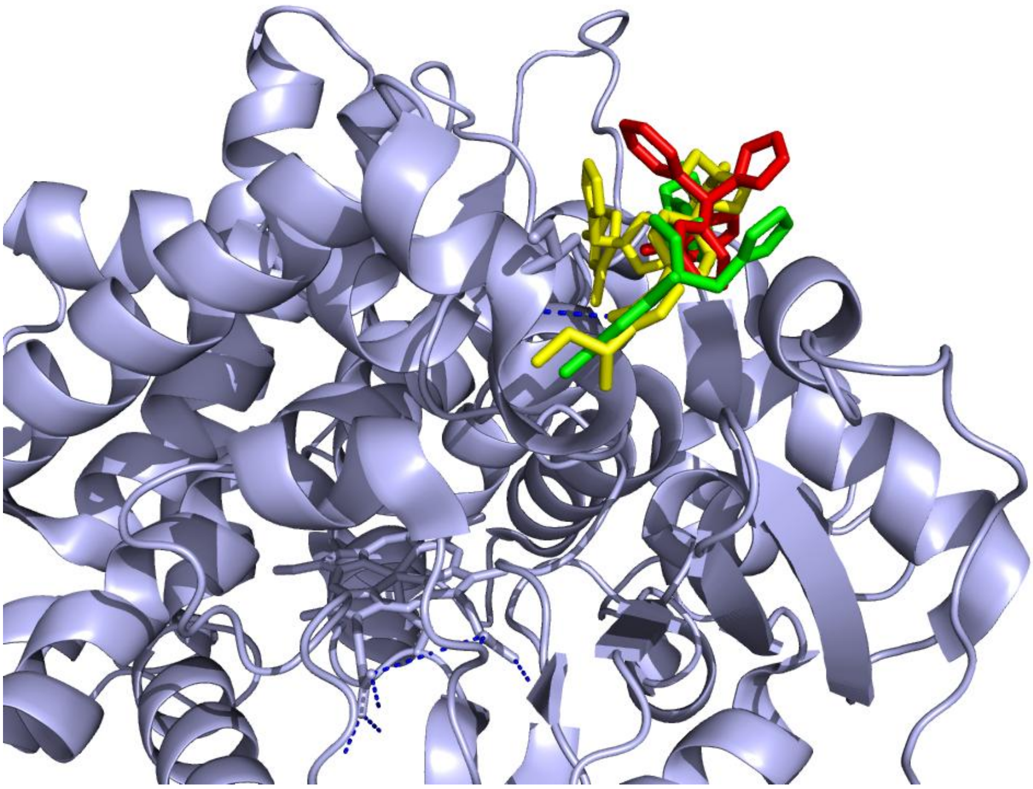

Clotrimazole – Red, Ketoconazole – Green, Itraconazole – Yellow

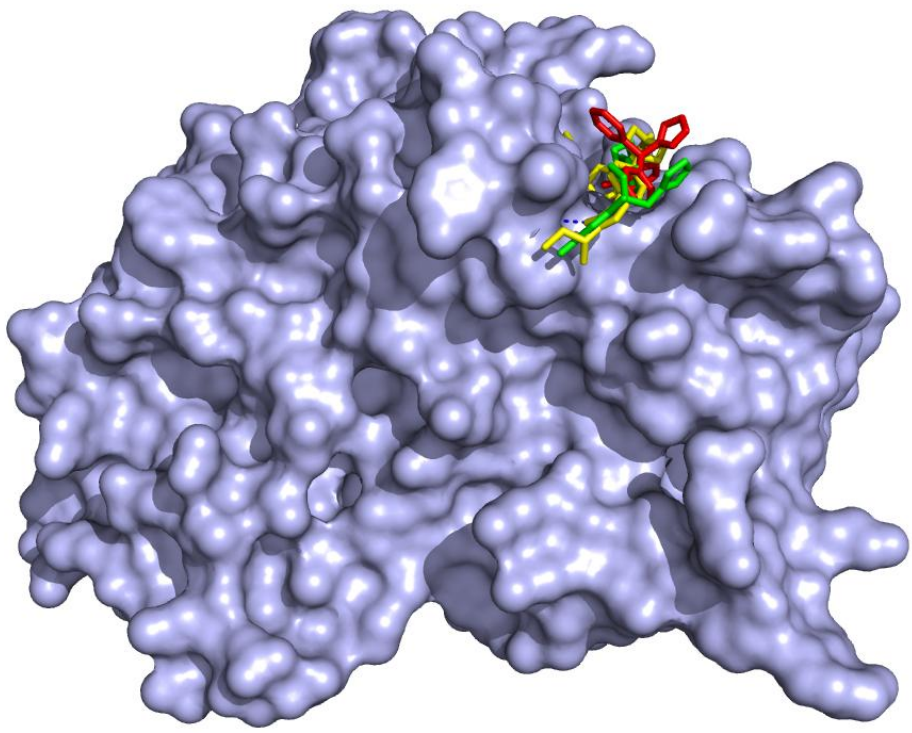

Figure A1P: Proximal surfaces of microsomal CYPs with the solvent-accessible thiolate marked yellow.

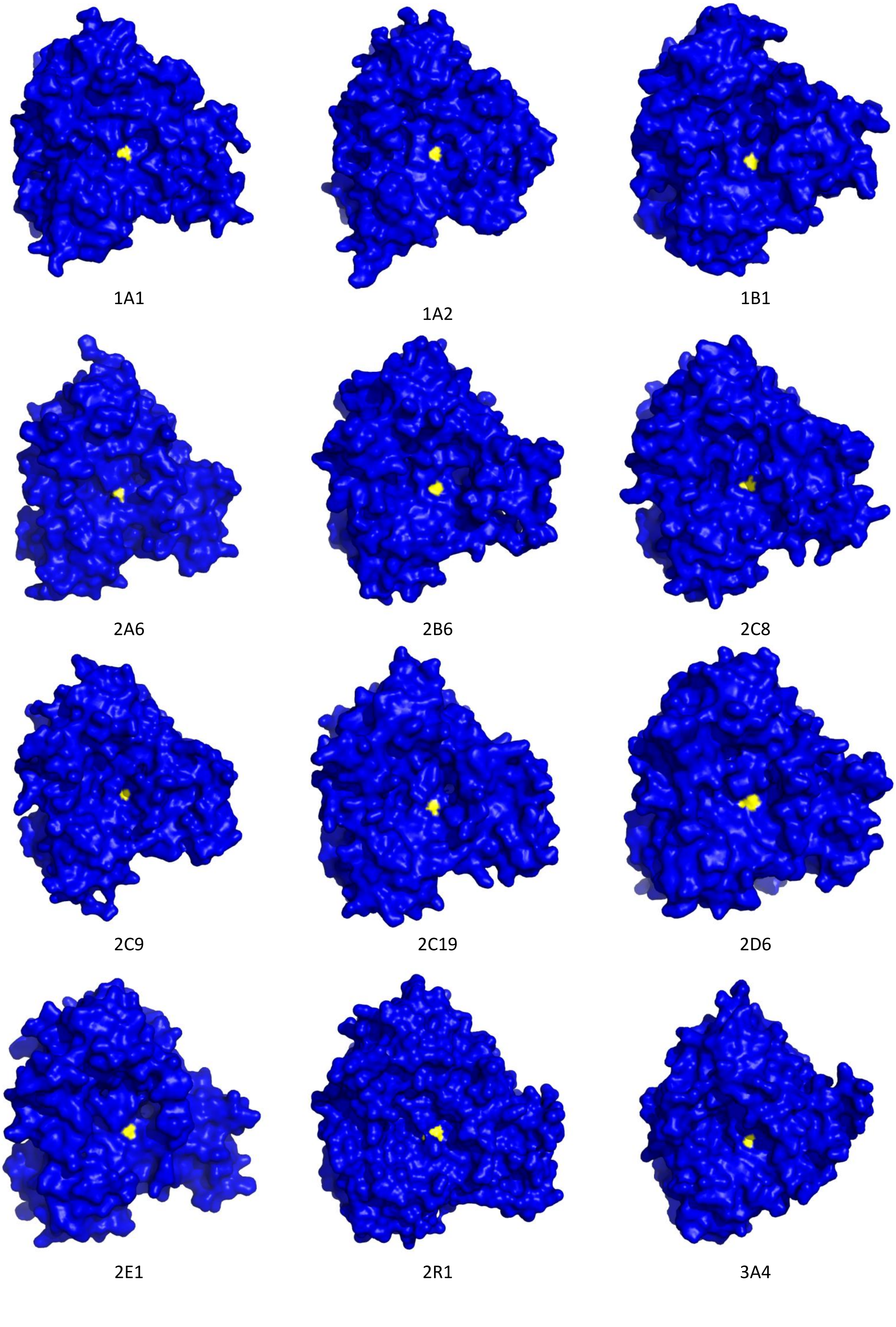

Figure A1Q: Various microsomal CYPs' topological survey. Thiolate – Yellow, TMS/hydrophobic helices-Red, Tunnel opening at the proximal side – Green, Tunnel opening at the distal side – Pink, Rest of the protein – Blue.

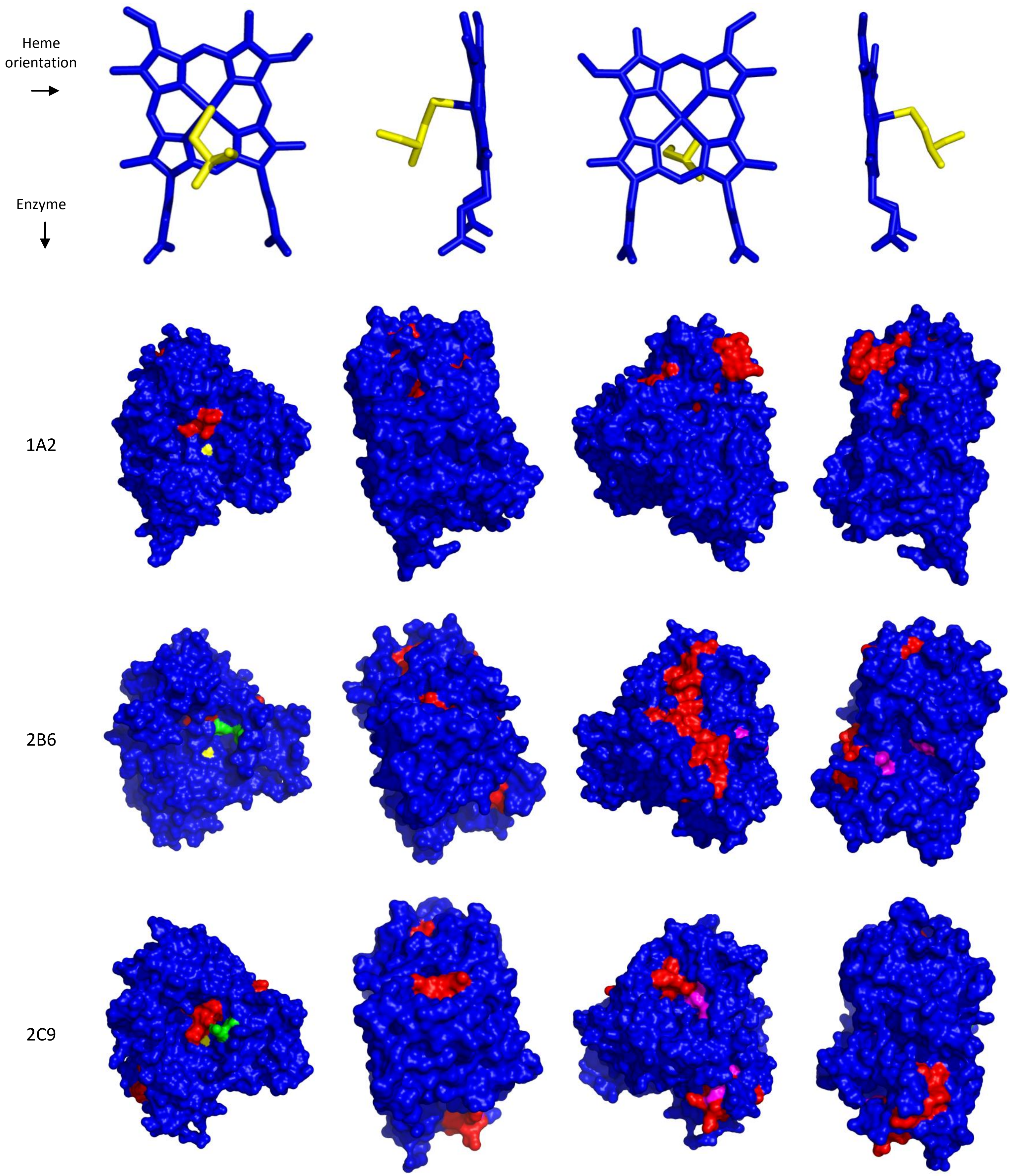

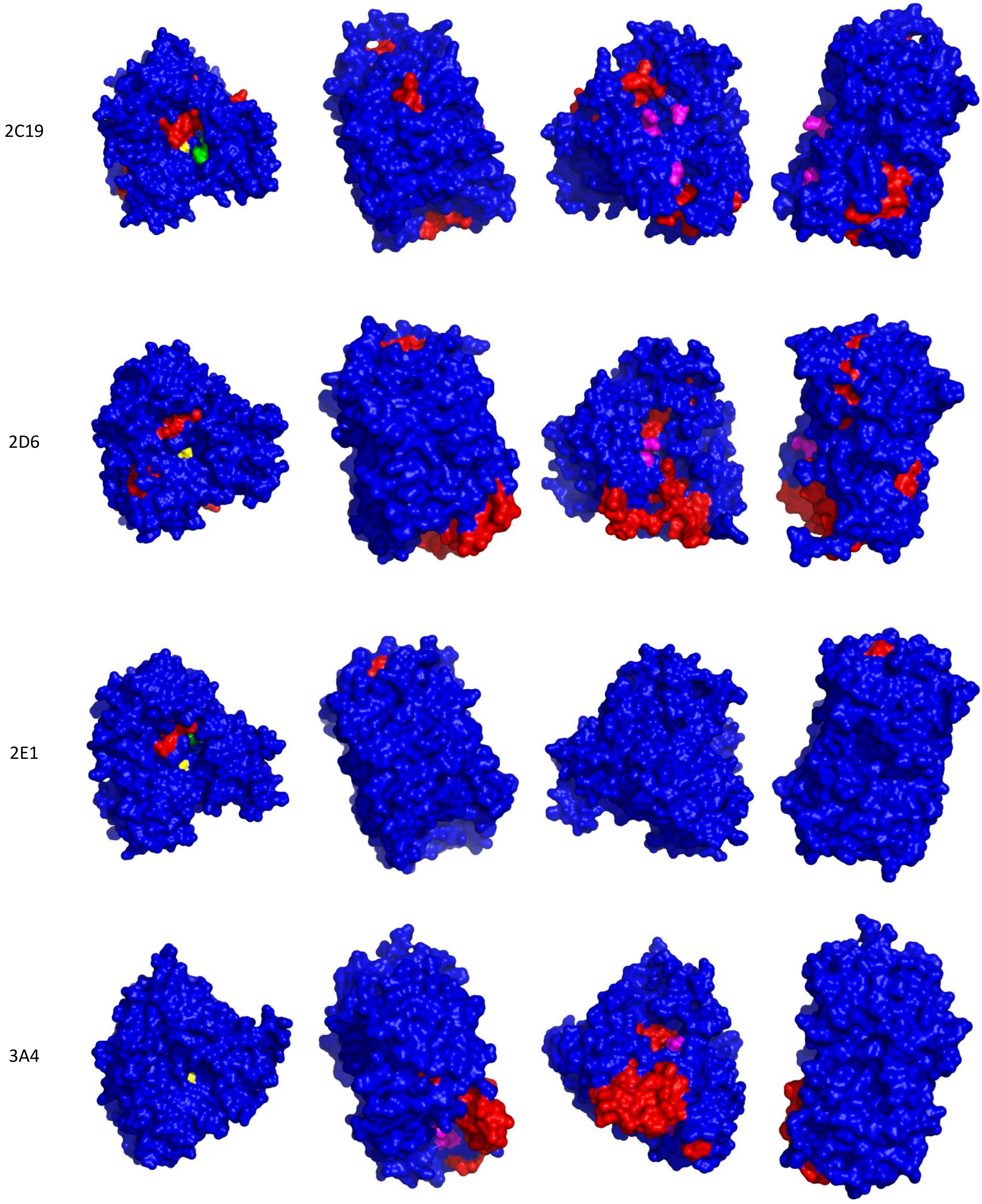
2C19
2D6
2E1
3A4
